# Rate Coefficients and Branching Ratios for the Reaction of the OH Radical with Formic Acid under Low-Temperature Combustion Conditions and the Fate of the HOCO Product

**DOI:** 10.1021/acs.jpca.5c01814

**Published:** 2025-07-02

**Authors:** Mark A. Blitz, Poppy Guy, Robin Shannon, Paul W. Seakins

**Affiliations:** † 4468 University of Leeds, Leeds LS2 9JT, United Kingdom; ‡ National Centre for Atmospheric Science (NCAS), University of Leeds, Leeds LS2 9JT, United Kingdom

## Abstract

Formic acid (FA, HC­(O)­OH) is of interest as an e-fuel and hydrogen carrier, as well as a significant component of atmospheric acidification. Reaction with the OH radical is a major removal process for FA. We have measured the overall rate coefficient, *k*_1_, for the reaction of OH and FA over the temperature range of 300–850 K using laser flash photolysis to generate OH and monitoring the disappearance of OH under pseudo-first-order conditions using laser-induced fluorescence. The rate coefficient can be parameterized as *k*_1_(*T*) = 9.8 × 10^–15^ × (*T*/298 K)^5.1^ × exp­(−14200/R*T*) cm^3^ molecule^–1^ s^–1^ where we estimate that the uncertainty increases from ∼ ±20% in the range 300–600 K to ±50% above 600 K. We have also determined the branching ratio to H_2_O + HCO_2_ (abstraction at the O–H site) by observing the H atoms produced by the fast decomposition of HCO_2_. H atom yields drop from ∼0.9 at 300 K to ∼0.5 at 600 K. The kinetics and branching ratios of *k*_1_ are in good agreement with theoretical calculations. Above 600 K, the observed H atom yield increases, and we show that this is due to the decomposition of the HOCO product. The implications of these studies on FA and HOCO in the combustion and pyrolysis of FA are considered.

## Graphical abstract

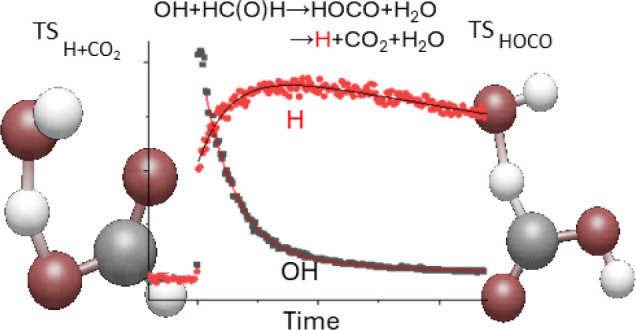

## 1 Introduction

Formic acid (FA, HC­(O)­OH, methanoic acid) is an important atmospheric constituent associated with aerosol growth and acidification. Significant uncertainties exist in the budget of organic acids;^1,2^ reaction with the OH radical is the main gas-phase removal process. There is also considerable interest in the role of FA as a hydrogen carrier^3^ and an e-fuel,^4^ possibly blended with other fuels.^5^ FA is produced in the low-temperature combustion of oxygenated fuels^6,7^ or during biomass combustion, with wildfires being a significant atmospheric source.^8,9^

The reaction of OH is a significant removal process for FA in both atmospheric and low-temperature combustion chemistry.^10^

R1a $$\mathrm{OH}+\mathrm{HC}\left(O\right)\mathrm{OH}\to H_{2}O+{\mathrm{HCO}}_{2},\Delta_{r}H_{298K}=-28.5{\mathrm{kJ mol}}^{-1}$$

R1b $$\mathrm{OH}+\mathrm{HC}\left(O\right)\mathrm{OH}\to H_{2}O+\mathrm{HOCO},\Delta_{r}H_{298K}=-85{.\mathrm{1 kJ mol}}^{\mathrm{‐1}}$$

Direct rate coefficient measurements of *k*_1_ are limited to the range of 300–500 K, and *k*_1_ exhibits virtually no temperature dependence over this range, with IUPAC^11^ recommending a value of *k*_1_ = $\left(4.5_{-1.3}^{+1.9}\right)$ × 10^–13^ cm^3^ molecule^–1^ s^–1^. The measured value of *k*_1_, isotopic studies, and calculations suggest a mechanism involving initial complex formation with a submerged, but entropically constrained, barrier to products. Previous measurements of *k*_1_, using a variety of techniques, are in good agreement.^12–15^

OH can abstract from either the O–H (*k*_1a_) or C–H bonds (*k*_1b_) forming HCO_2_ and HOCO, respectively. HCO_2_ has a relatively low barrier to dissociation (16.5 kJ mol^–1^)^16^ with essentially instant production of H + CO_2_ at temperatures of 300 K and above.

R2 $${\mathrm{HCO}}_{2}\to {H+\mathrm{CO}}_{2}$$

HOCO is stable ( $k_{3}^{\infty }$ < 10 s^–1^) at temperatures of 600 K and below.^17^ Dissociation leads to either OH + CO ([Disp-formula ueq4]) or H + CO_2_ ([Disp-formula ueq5]), with OH + CO expected to be the dominant product.^17^

R3a HOCO→OH+CO

R3b $$\mathrm{HOCO}\to {H+\mathrm{CO}}_{2}$$

Modeling studies by Marshall and Glarborg,^18^ based on the calculations of Anglada,^19^ illustrate the importance of knowing the branching ratio for R1 under low-temperature combustion conditions. Channel [Disp-formula ueq1], abstraction at the acidic site and effectively forming H + CO_2_ (via the rapid [Disp-formula ueq3] reaction), has a large positive sensitivity for formic acid combustion, as it leads to chain branching via [Disp-formula ueq6].

R4 $${H+O}_{2}\to \mathrm{OH}+O$$

Conversely, channel [Disp-formula ueq2] has a significant negative sensitivity as the HOCO formed reacts with O_2_ ([Disp-formula ueq7]), generating the relatively inert HO_2_ radical.

R5 $${\mathrm{HOCO}+O}_{2}\to {\mathrm{HO}}_{2}+{\mathrm{CO}}_{2}$$

In the atmosphere, HO_2_ and CO_2_ will be the effective products of both channels, as H atoms from [Disp-formula ueq1] will rapidly combine with O_2_ to form HO_2_.

Room temperature measurements suggest that [Disp-formula ueq1] dominates; Wine et al.^12^ observed a significant H atom yield, presumed to be from [Disp-formula ueq3], and Singleton et al.^14^ observed a kinetic isotope effect when O–H was selectively deuterated, but virtually no change in *k*_1_ with deuteration of the C–H bond. No measurements exist regarding the branching ratio as a function of temperature, despite the importance of this reaction.

A number of *ab initio* calculations have been carried out on R1, examining the overall kinetics, branching ratios, and the role of water complexation. Galano et al.,^20^ Anglada,^19^ and Sun and Saeys^21^ predict overall rate coefficients in good agreement with experimental data around room temperature, and they find that abstraction at the acidic site ([Disp-formula ueq1]) dominates. Although the barrier to abstraction at the acidic site is higher, tunneling is more facile at this site. The fraction of reaction at the acidic site is calculated to decrease with temperature, with the two channels becoming equal at temperatures of ∼450–570 K. In contrast, Elm et al.^22^ calculate that abstraction at the C–H site dominates at room temperature, although the calculated branching ratio is sensitive to the *ab initio* method and the tunneling correction used. The theoretical calculations of Mendes et al.^23^ also predict that abstraction at the C–H site, forming HOCO, dominates at room temperature, and, as with other studies, this channel increases with temperature. Several studies have examined the impact of water complexation^24–26^ which can enhance rates and alter branching ratios, but this is not the focus of our study.

Combustion models involving formic acid use a wide range of rate coefficients and branching ratios for R1. Wako et al.,^27^ Sarathy et al.,^4^ Lavadera and Konnov,^28^ and Marshall and Glarborg^18^ use values for [Disp-formula ueq1] and [Disp-formula ueq2] based on the calculations of Anglada. However, the standard Aramco model and other models (e.g., Fischer et al.^29^ for dimethyl ether oxidation) use values from Marinov^30^ which predict that abstraction at the acid site is a minor component at room temperature but increases with temperature and dominates (∼70%) at 900 K.

Yin et al.^31^ carried out a combined experimental (jet-stirred reactor), computational, and modeling study on formic acid pyrolysis and oxidation. Their experimental results suggested faster pyrolysis than that predicted by the Marshall and Glarborg model and indicated that an addition-elimination reaction ([Disp-formula ueq8]), generating H atoms, could account for the observed enhancement in the rate.

R1c $$\mathrm{OH}+\mathrm{HC}\left(O\right)\mathrm{OH}\to H+{\left(\mathrm{HO}\right)}_{2}\mathrm{CO}$$

Their calculated barrier of 22.6 kJ mol^–1^ means that the reaction is significant only at high temperatures.

Clearly, there is a need for direct experimental determinations of the kinetics and branching ratio of reaction R1 at temperatures relevant to low-temperature combustion. In this study, we present results on the overall rate coefficient, *k*_1_, from 300 to 850 K, determined by pulsed laser photolysis of a range of OH precursors, with detection of OH via laser-induced fluorescence (LIF) and direct detection of the H atom product (again via LIF) from [Disp-formula ueq1], followed by [Disp-formula ueq3] from 300 to 600 K. At higher temperatures, there is evidence of an additional source of H atoms that we attribute to HOCO decomposition, in contrast to expectations. H atom yields from HOCO are explored at higher temperatures via the reaction of Cl atoms with FA and acidic photolysis at 266 nm, both of which are clean sources of HOCO with no HCO_2_ formation.

## 2 Experimental and Theoretical Methodology

### 2.1 Apparatus

Reactions were studied using the pulsed laser photolysis technique, with the removal of OH and production of H both monitored via laser-induced fluorescence (LIF). Two coupled, multi-port reaction cells were used (see Figure S1 for a schematic and Section S1 for further details), similar in operation to those described in previous studies.^32,33^ The reaction gas mixture (OH precursor, HC­(O)­OH, and bath gas) could be flowed in either direction through the two cells. The temperature (measured using Type K thermocouples) and pressure (measured using capacitance manometers) in each cell were monitored independently. Each cell was surrounded by a ceramic oven, allowing temperatures of up to 850 K to be achieved.

The photolysis laser (excimer laser at 193 or 248 nm, or Nd:YAG laser at either 213, 266, or 355 nm) was passed directly through both reaction cells. In reactor 1, OH probe radiation at ∼308 nm (Nd:YAG laser at 532 nm pumping a Sirah dye laser with DCM special dye, giving output at 616 nm, which was then doubled in a BBO crystal) was introduced perpendicularly to the photolysis beam. Resonant OH fluorescence was detected by a gated channel photomultiplier (CPM, C943 Proxivision) mounted perpendicular to the plane of the photolysis and probe lasers. The signal from the CPM was monitored on a multiple-channel oscilloscope (LeCroy) and passed to a PC for analysis. A LabView program controlled the time delay between the photolysis and probe lasers to build up OH time profiles similar to those shown as the black squares in Figure 1.

**Figure 1:**
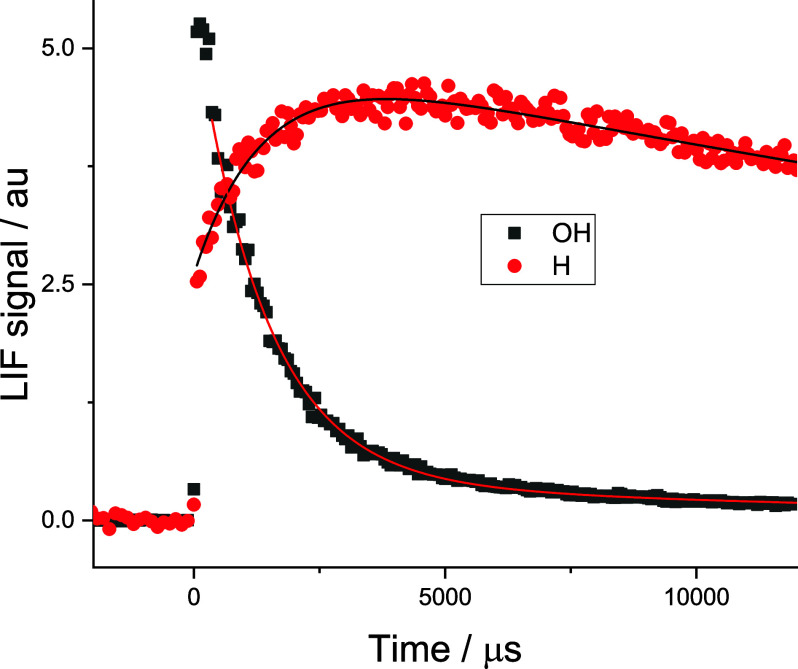
Typical OH (black ■) and H (red ●) time profiles following the photo-oxidation of HC­(O)­OH at 213 nm. [HC­(O)­OH] = 3.2 × 10^15^ molecules cm^–3^ at 373 K. The simultaneous fit to the H growth and OH loss is (726 ± 35) s^–1^. The total pressure is 53 Torr (Ar).

In reactor 2, Lyman α radiation (121.56 nm) was generated by frequency tripling light at 364.7 nm (Nd:YAG laser at 532 nm pumping a Sirah dye laser with a Pyridine 1 and 2 dye mix, giving output at 729.4 nm, which was then doubled in a BBO crystal) in a Kr/Ar mix (∼1:2.5 at a total pressure of ∼ 1000 Torr) housed in a glass cell. The 364.7 nm light was gently focused (15 cm focal length lens) into the tripling cell. The MgF_2_ window of the tripling cell coupled the Lyman α radiation directly into reactor 2. As with reactor 1, resonant fluorescence was detected perpendicular to the plane of the photolysis and probe lasers with a second CPM (C911, Proxivision). A typical H atom time trace is shown as the red circles in Figure 1, with prompt H atom production from HC­(O)­OH photolysis and the growth in H atoms from Reaction [Disp-formula ueq1] followed by the essentially instant decomposition of HCO_2_ ([Disp-formula ueq3]). A second photomultiplier tube (solar blind, Thorn EMI) was mounted opposite the tripling cell with the same path length from the observation region (where photolysis and probe lasers cross). This second PMT allows us to normalize the H atom fluorescence for fluctuations in the efficiency of the tripling cell. The H atom fluorescence detection system is similar to that used in our previous work,^34,35^ but the shorter pulse width of the Nd:YAG-pumped system provides more efficient conversion than the previous excimer-pumped system. Formic acid also has a large and well-characterized absorption cross-section at 121.56 nm ((1.32 ± 0.07) × 10^–17^ cm^–2^,^36^ which was used to determine [HC­(O)­OH] via the Beer–Lambert law.

### 2.2 Determination of OH + HC­(O)­OH Overall Rate Coefficients

A major issue with formic acid is its propensity to dimerize (see S2 of SI). The equilibrium constant for dimerization is well characterized (see Singleton et al.^14^ and references therein), and therefore, conditions under which dimerization will occur can be avoided. Our ability to directly observe HC­(O)­OH absorption helps confirm that experiments are being carried out under conditions where HC­(O)­OH only exists as the monomer. Figure S2 in SI shows excellent agreement between the predicted and observed VUV absorptions up until a [HC­(O)­OH] ∼2–3 × 10^15^ molecules cm^–3^, where dimerization occurs and absorption changes. Dimerization becomes increasingly less important above room temperature.

Reaction 1 was studied under pseudo-first-order conditions with [OH] ≪ [HC­(O)­OH]. Under these conditions, the rate of loss of OH is given by

E1 $$\frac{-d\left[\mathrm{OH}\right]}{dt}=k_{1}^{’}\left[\mathrm{OH}\right]$$

where $k_{1}^{’}$ = *k*_1_[HC­(O)­OH] + *k*_d_ and *k*_d_ represents other loss processes such as diffusion and reaction with the precursor (if used) that can be approximated to first- or pseudo-first-order processes. The equation integrates to

E2 $${[\mathrm{OH}]}_{t}={[\mathrm{OH}]}_{0}e^{-k_{1}^{’}t}$$

As the OH LIF signal, *S*_OH_, is proportional to [OH], eq [Disp-formula ueq10] becomes

E3 $${[S]}_{\mathrm{OH},t}={[S]}_{\mathrm{OH},0}e^{-k_{1}^{’}t}$$

$k_{1}^{’}$ can be extracted from a nonlinear least-squares fit to the *S*_OH_ trace (see inset Figure 2), and the bimolecular rate coefficient *k*_1_ is obtained by varying the concentration of formic acid and plotting the resultant values of $k_{1\;}^{’}vs\;\left[\mathrm{HC}\left(O\right)\mathrm{OH}\right]$ , as shown in Figure 2.

**Figure 2:**
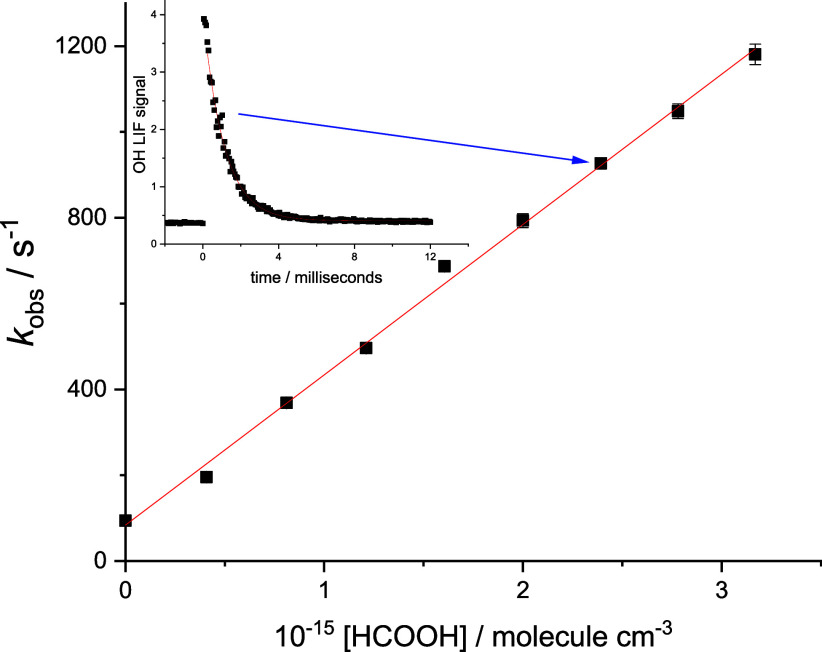
Typical bimolecular plot at 298 K, 50 Torr Ar, with the slope equal to *k*_1_ = (3.50 ± 0.14) × 10^–13^ cm^3^ molecule^1^ s^–1^, where the error is statistical at the 2σ level, and intercept (84 ± 13) s^–1^ which is typical for diffusion under these conditions. The blue line indicates the point obtained from the fit (906 ± 13 s^–1^) from the trace shown in the inset.

### 2.3 Determination of H Atom Yields

Fluorescence is proportional to the concentration of the species, either OH or H, and therefore, in order to determine the branching ratio for Reaction 1, we need to be able to quantitatively relate the OH and H signals. This is achieved via the use of a calibration reaction;^37^ in this case, the reaction of OH with excess H_2_ (Reaction [Disp-formula ueq12]) is as follows:

R6 $${\mathrm{OH}+H}_{2}\to H_{2}O+H$$

Figure 3 shows an example of the method used. In this case, formic acid photolysis at 213 nm was used as the OH source, but at this wavelength, H atoms are also produced during photolysis; hence, the prompt signal is at *t* = 0. The first trace recorded (black square, ■) was with just formic acid present. Following prompt photolytic production, the H atom signal grows in with a rate coefficient characteristic of R1. At longer times, H atoms are removed by diffusional loss and chemical loss. Fitting the formic acid-only trace for growth and loss allows determination of the H atom signal from R1. Then, under identical photolysis conditions (as seen by the identical prompt H signal in the red circle (●) trace), a large excess of H_2_ is added to titrate OH to H via Reaction [Disp-formula ueq12]. Both traces are fitted simultaneously to allow the extraction of the branching ratio of [Disp-formula ueq1]. Further details can be found in Section S3 of SI.

**Figure 3:**
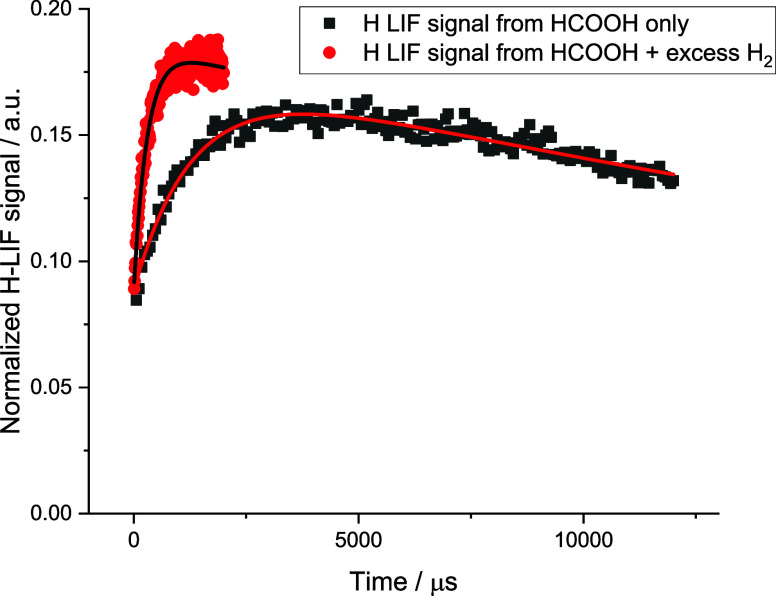
Typical traces to determine absolute H atom yields from Reaction 1. These experiments were performed at 373 K with a total pressure of ∼ 50 Torr of Ar. (black ■): [HC­(O)­OH] = 3.13 × 10^15^ molecules cm^–3^ and (red ●): [H_2_] = 7.38 × 10^16^ molecules cm^–3^ and [HC­(O)­OH] = 3.23 × 10^15^ molecules cm^–3^.

### 2.4 Calculations

The OH + formic acid reaction and the subsequent decomposition of the HOCO product were investigated theoretically using electronic structure theory calculations coupled with statistical rate theory. To calculate accurate stationary point energies for the transition states and wells, we have used a variation of the ANL family of methods.^10^ Specifically, we have used a form described in our previous publication^38^ which involves the following electronic structure theory methods and corrections:

E4 $$E_{\mathrm{ANL}-\mathrm{like}}=E_{\mathrm{CCSD}\left(T\right)-F12/\mathrm{cc}-\mathrm{pVQZ}-F12}+\Delta E_{\mathrm{anharm}}+\Delta E_{\mathrm{quad}}+\Delta E_{\mathrm{core}}+\Delta E_{\mathrm{DK}}$$

where

$$\Delta E_{\mathrm{anharm}}=ZPE_{\mathrm{CCSD}\left(T\right)-F12/\mathrm{cc}-\mathrm{pVDZ}-F12}^{\mathrm{harmonic}}+\left(ZPE_{B3\mathrm{LYP}/6-311+G^{**}}^{\mathrm{anharmonic}}-ZPE_{B3\mathrm{LYP}/6-311+G^{**}}^{\mathrm{harmonic}}\right)$$

$$\Delta E_{\mathrm{quad}}=E_{\mathrm{CCSDT}\left(Q\right)/\mathrm{aug}-\mathrm{cc}-\mathrm{pVDZ}}-E_{\mathrm{CCSD}\left(T\right)/\mathrm{aug}-\mathrm{cc}-\mathrm{pVDZ}}$$

$$\Delta E_{\mathrm{core}}=E_{\mathrm{CCSD}\left(T,\mathrm{full}\right)/\mathrm{aug}-\mathrm{cc}-\mathrm{pCVTZ}}-E_{\mathrm{CCSD}\left(T\right)/\mathrm{aug}-\mathrm{cc}-\mathrm{pCVTZ}}$$

$$\Delta E_{\mathrm{DK}}=E_{\mathrm{CCSD}\left(T,\mathrm{DK}\right)/\mathrm{cc}-\mathrm{pVTZ}-\mathrm{DK}}-E_{\mathrm{CCSD}\left(T\right)/\mathrm{cc}-\mathrm{pVTZ}-\mathrm{DK}}$$

For the HOCO decomposition kinetics, we slightly increased the basis sets for the geometry optimizations and CCSD­(T) single-point energies from cc-pVDZ-F12 to cc-pVTZ-F12 and cc-_P_VQZ-F12 to cc-pV5Z*-*F12, respectively. All DFT calculations in the current work were performed using the Gaussian 09 software,^39^ the calculations for the Δ*E*_quad_ correction were performed using MRCC,^40^ and all other calculations were performed in Molpro.^41^

One notable omission compared to the original ANL0 scheme is that we are not currently able to include corrections for spin–orbit coupling. We also, in general, use smaller basis sets and omit the complete basis set extrapolations employed in the original ANL approaches. We have mitigated this to some extent using explicitly correlated F12 methods.^42^ We estimate total uncertainties of the order of 1 to 2 kJ mol^–1^ on our stationary point energies.

To capture anharmonicity in the various species considered in this work, all hindered rotation or torsional modes are treated explicitly. The underlying 1D or 2D torsional potentials were formed using M062*X*/6-31+G** constrained optimizations over 30-degree increments of the corresponding dihedral angles.^43^ The M062*X*/6-31+G** method was also used to perform IRC calculations at the various reaction saddle points. These calculations formed the basis of one-dimensional tunneling potentials, which were further refined by M062*X*/6-31+G** projected frequency calculations to incorporate zero-point energy and CCSD­(T)-F12/aug-cc-pVTZ-f12 single-point energies at each point.

With the above ingredients, rate coefficients for both OH + formic acid and HOCO decomposition reactions could be calculated using the master equation software MESMER.^44^ These calculations were performed with an energy grain size of 50 cm^–1^ by using the zero-point energy-corrected stationary point energies obtained above. Energy transfer was modeled using an exponential down model parameterized by the average energy transferred upon downward collision with a bath gas molecule (⟨Δ*E*_*d*_⟩), and collision frequencies were calculated assuming a Lennard-Jones potential between the collision partners. In practice, energy transfer is only important in the HOCO decomposition case since none of the prereaction complexes in the OH + formic acid system exhibit sufficient lifetimes to be collisionally stabilized by the bath gas under the conditions of interest here. For HOCO decomposition, a temperature-dependent ⟨Δ*E*_*d*_⟩ was assumed according to the following expression:

E5 $$\langle \Delta E_{d}\rangle =\langle \Delta E_{d}\rangle \left(298\;K\right){\left(\frac{T}{298}\right)}^{n}$$

Both ⟨Δ*E*_*d*_ ⟩(298 K) and *n* were fitted to the experimental rate coefficients using a built-in Levenberg–Marquardt method in MESMER. However, because studies were carried out over a limited temperature range, equally good fits could be produced with *n* fixed.

Molecular densities of states for various species were calculated by assuming rigid rotations and harmonic vibrational frequencies for most modes. For torsional modes, a fully coupled hindered approach was used to accurately determine the contribution of these torsions to the molecular densities of states.^38^ Such a treatment implicitly incorporates any conformational flexibility in a given molecule; however, we presently make the approximation that the nontorsional modes are invariant to the particular conformation of a species. We estimate that the effective uncertainty on the calculated rate coefficients associated with these approximations is equivalent to the 1–2 kJ mol^–1^ uncertainty associated with the particular barrier heights.

A final consideration for the MESMER calculations is quantum mechanical tunneling. In this work, we have primarily used a one-dimensional WKB approach incorporating the potentials derived from IRC calculations as described above. We have also calculated tunneling transmission coefficients assuming an asymmetric Eckart potential parametrized by the imaginary frequency of the particular rate coefficient.

## 3 Results and Discussion

### 3.1 Overall Kinetics of Reaction 1

Experiments to determine *k*_1_ were repeated over a range of temperatures and pressures, using a variety of OH precursors, including H_2_O_2_ photolysis at 248 or 266 nm, acetylacetone (ACAC) at 248 or 266 nm, and formic acid itself at either 193, 213, 248, or 266 nm. The early OH experiments used either 248 nm photolysis of formic acid or 266 nm photolysis of ACAC at ca. 10 Torr total pressure in Ar. While these experiments were consistent with the results presented below, the majority of the experiments used direct photolysis of formic acid at 193, 213, and 266 nm at a total pressure of ∼ 50 Torr; there were a few experiments at 266 nm where H_2_O_2_ was the main OH precursor. A full set of experimental results and conditions presented in this article can be found in Table S1.

Previous studies^12–15^ have been carried out over the temperature range of 298–445 K, and over this temperature range, our results are in good agreement with the literature, as shown in Figure 4. Our room temperature value for *k*_1_ = (3.44 ± 0.48) × 10^–13^ cm^3^ molecule^1^ s^–1^ based on the average of eight measurements, where the error represents the 95% confidence interval (Figure S4 in Section S4 of SI) of 3.4 × 10^–14^ cm^3^ molecule^1^ s^–1^, combined in quadrature with an estimated 10% systematic error. This value is at the lower end of the recommended range given by IUPAC^11^ (*k*_1_ = $\left(4.5_{-1.3}^{+1.9}\right)$ × 10^–13^ cm^3^ molecule^–1^ s^–1^) but in good agreement with Zetzsch and Stuhl^45^ ((3.2 ± 0.2) × 10^–13^ cm^3^ molecule^–1^ s^–1^) and Dagaut et al.^15^ ((3.7 ± 0.4) × 10^–13^ cm^3^ molecule^–1^ s^–1^).

**Figure 4:**
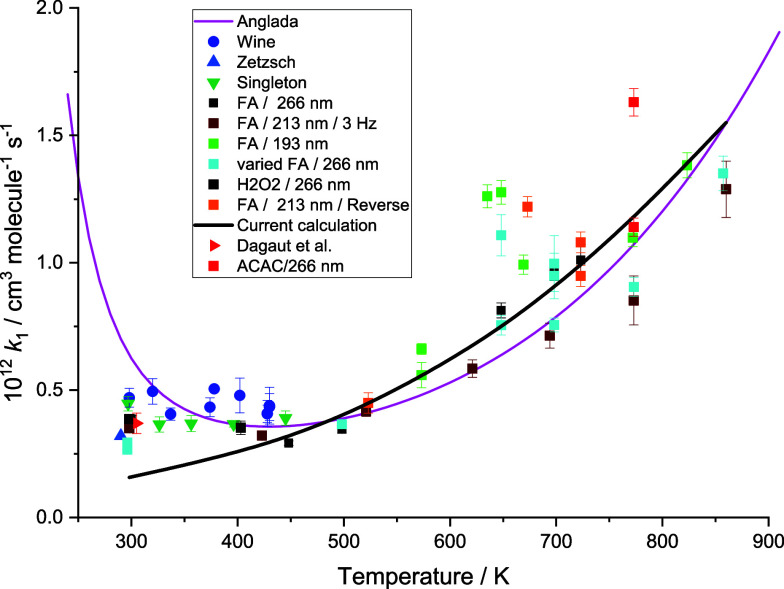
Data for *k*_1_ from 295 to 860 K from this work, shown as squares of varying color, with error bars showing the 2σ statistical errors from the bimolecular fit; “varied” means that the flow rate was different from the standard 10 sccm/Torr, thus giving a different residence time in the cell; “reverse” means that the direction of flow through the cells was reversed. Literature data from previous studies are also shown. Wine et al.^12^ (blue ●), Zetzsch (blue-gray ▲, 298 K offset for clarity), Singleton et al.^14^ (green ▼), and Dagaut et al.^15^ (red ▶, 298 K, but offset for clarity). Solid lines are theoretical calculations; this work with WKB correction (black line) which can be parametrized as *k*_1_ = 8.5 × 10^–23^× *T* ^3.4^ × exp­(580/*T*), Anglada^19^ (magenta line).

Values for *k*_1_ behaved sensibly up to ∼600 K, with the rate coefficient gradually increasing as predicted by the calculations of Anglada.^19^ Above 600 K, results became significantly more scattered; mainly, we believe due to secondary chemistry either from photolysis coproducts (e.g., the complex photolysis of acetylacetone^46^) or from the products of Reaction 1. It is likely that a significant source of the complexity comes from reactions such as [Disp-formula ueq19]:^11^

R7 $$H+{\mathrm{HO}}_{2}\to 2\mathrm{OH}\;,k_{7}=7.2\times 10^{-11}\;{\mathrm{cm}}^{3}\;{\mathrm{molecule}}^{\mathrm{-1}}\;s^{-1}$$

where HO_2_ can be generated from the reaction of HCO or H (products of HC­(O)­OH photolysis) with O_2_ present either from trace leaks or H_2_O_2_ decomposition. In a number of experiments over the range 625–800 K, the addition of O_2_ resulted in a dramatic reduction of the measured rate coefficient, which was typically between 2 and 3 × 10^–13^ cm^3^ molecule^–1^ s^–1^. The system took many minutes to recover after turning off the added O_2_. Careful experiments controlling radical concentrations to low levels, minimizing leaks, and working at low laser repetition rates produced the consistent values shown in Figure 4. Despite careful control of conditions, it can be seen that data above 600 K are more scattered. Decomposition of HOCO, discussed below, contributes additional secondary chemistry and may explain the increased scatter of data points.

A weighted fit through our experimental data gives

$$k_{1}\left(T\right)=9.8\times 10^{-15}\times {\left(T/298K\right)}^{5.1}\times \exp\left(-14200/RT\right)\;{\mathrm{cm}}^{3}\;{\mathrm{molecule}}^{-1}\;s^{-1}$$

where we estimate that the overall uncertainty in this expression increases from ∼ ±20% between 300 and 600 K to ± 50% above 600 K, reflecting the scatter in the data shown in Figure 4. The larger errors reflect the scatter of our data and the difficulty of performing these experiments due to the photolysis of FA at most photolysis wavelengths and, hence, the potential for fast secondary radical–radical reactions that can compete with the relatively slow target reaction. Although FA can be used as an OH precursor, photolysis to HCO + OH is not the sole channel of the reaction (see SI, section S5), and secondary chemistry arising from HCO can interfere. OH precursors do exist for wavelengths greater than 280 nm, where FA no longer dissociates, but such precursors can require oxygen (e.g., Carr et al.^47^), or be thermally unstable and a source of NO (e.g., HONO^48^ ).

### 3.2 H Atom Yields from Reaction 1 at *T* < 600 K

HCO_2_ formed from Reaction [Disp-formula ueq1] (Δ_r_ *H*_298 K_ = −28.5 kJ mol^–1^)^10^ rapidly falls apart to H + CO_2_ ([Disp-formula ueq3]). It is an academic question whether decomposition occurs via chemically activated products from the bimolecular reaction^49^ as, due to the small barrier for dissociation, thermal decomposition will be rapid compared to the time scale of R1. At temperatures below 600 K, HOCO decomposition, whether to OH + CO or H + CO_2_, is very slow.^17^ Depending on the photolysis precursor, there can also be an instant source of H; however, the HCO_2_ product from H + FA is thermodynamically inaccessible.^10^ Therefore, from 300 to 600 K, the only source of H atoms, on the time scale of OH removal, is via [Disp-formula ueq1] followed by [Disp-formula ueq3], and the H atom signal can be compared with the initial OH via the calibration procedure described above. As R1 is the rate-determining step in H atom production, the pseudo-first-order rate coefficient for H atom growth should match that of OH decay, as shown in Figure 1, confirming [Disp-formula ueq1] as the source of H atoms below 600 K. Below ∼600 K, the H atom yield shows a steady monotonic fall, in good agreement with the calculations of this work and Anglada.^19^

Figure 5 shows a plot of the H atom yield from 300 to 820 K. Above 600 K, the H atom yield begins to rise, and we believe this is due to the decomposition of HOCO, as described in Section 3.5.

**Figure 5:**
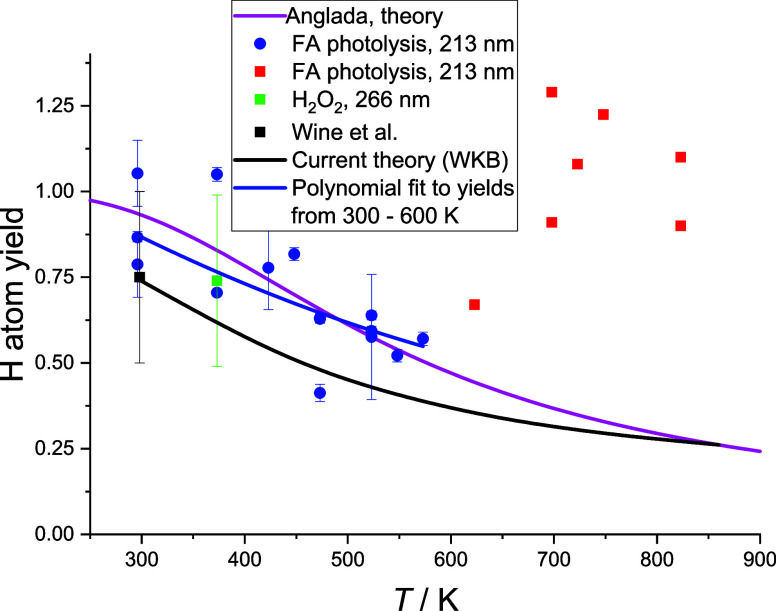
H atom yields from Reaction 1. (blue ●, 213 nm, green ■, 266 nm) this work under conditions of limited HOCO decomposition; (black ■) Wine et al.;^12^ (red ■) this work where HOCO decomposition enhances H atom yield. The solid lines are theoretical calculations from this work (black line) and Anglada^19^ (magenta line). The experimental yield from 300 to 600 K can be parametrized as H_yield_ = 1.41 – 2.1 × 10^–3^ × *T* + 1.1 × 10^–6^ × *T* ^2^ with errors of ± 25%. Our calculated yield can be parametrized as H_yield_ = (1.37 ± 0.05) – (2.61 ± 0.17) × 10^–3^ × *T* + (1.55 ± 0.15) × 10^–6^ × *T* ^2^.

### 3.3 Theoretical Calculations and Master Equation Fit to the Data for the OH + HC­(O)­OH Reaction and H Atom Branching Ratios

A schematic potential energy surface showing the stationary point energies at the ANL level for the OH + formic acid system is shown in Figure 6. When obtaining these ANL energies, it was noted that the Δ*E*_anharm_ correction was particularly large for TS_HOCO_ at −6.5 kJ mol^–1^. Furthermore, the B3LYP calculations for this transition state gave an imaginary frequency of 217 cm^–1^ compared to the reliable CCSD­(T)-F12 value of 1294 cm^–1^. Considering these factors, we also obtained the Δ*E*_anharm_ correction for this species at the MP2/6-311+G** level of theory, which gave a more modest Δ*E*_anharm_ of −3.4 kJ mol^–1^. The energy values along with the Δ*E*_anharm_ corrections are provided in Table 1, and we have chosen to use the MP2/6-311+G** Δ*E*_anharm_ correction for TS_HOCO_ in all subsequent calculations.

**Figure 6:**
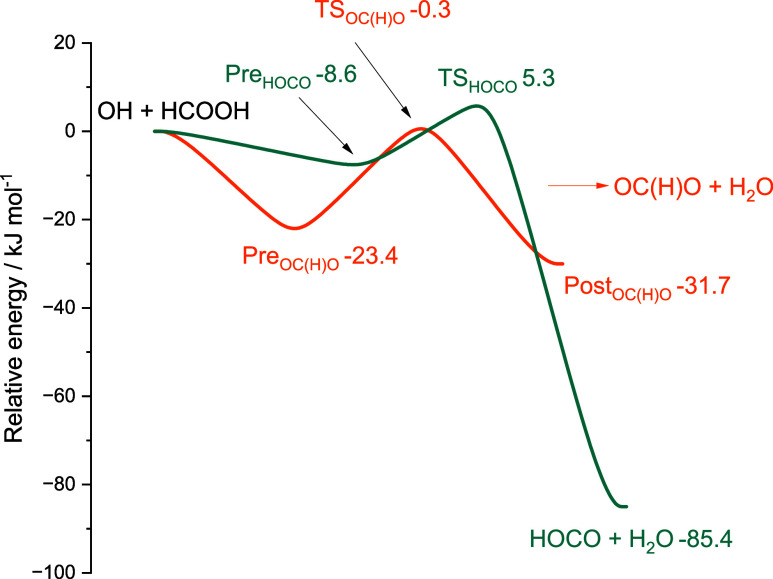
Stationary points for the OH + formic acid reaction are given with energy values calculated at the ANL level.

**Table 1:** Comparison of Energy Values (in kJ mol^–1^) for the Stationary Points on the OH + Formic Acid Potential Energy Surface

Stationary point energy (kJ mol^–1^)	This work^a^	$\Delta E_{\mathrm{anharm}}$	Anglada^b^ ^19^	Galano et al.^c^ ^20^	Sun and Saeys^d^ ^50^	Mendes et al.^e^ ^23^
PRC_OC(H)O_	–23.4	–2.0	–27.5	–15.4	–15.2	–31.4
TS_OC(H)O_	–0.3	–3.1	2.1	18.3	14.1	15.0
PRC_HOCO_	–8.6	–2.5	–14.4	–8.4	–14.8	–18.0
TS_HOCO_	5.3	–3.4^f^ (−6.5)	4.9	16.9	12.4	12.5
Post_OC(H)O_	–31.7	–2.3	–23.1	-	–22.1	–25.1
HCO_2_ + water					–28.4	–13.8
HOCO + water	–85.4	–1.6	–72.8	-	–77.7	–69.8

a Calculations at the ANL level.

b Calculations at the CCSD­(T)/aug-cc-pVTZ//QCISD/6-311+G­(2df,2p) level of theory.^19^

c Calculations at the CCSD­(T)/6-311++G­(2d,2p)//B3LYP/6-311++G­(d,p) level of theory.^20^

d Calculations at the CBS-QB3 level of theory.^50^

e Calculations at the CCSD­(T)/cc-pVXZ (X = D,T, and Q) level of theory.^23^

f Indicates the MP2/6-311+G** Δ*E*_anharm_ correction, whereas all other values are from B3LYP/6-311+G** calculations.

The OH + formic acid system has been studied previously, and results from earlier work on the stationary points are summarized in Table 1. The calculations performed here are expected to be significantly more accurate than those in previous studies. If we compare our estimated 1–2 kJ mol^–1^ uncertainty with the expected 4–6 kJ mol^–1^ uncertainty in the calculations of Anglada, both studies are found to agree within the combined uncertainties. The lower level calculations for the barrier heights, however, deviate significantly from both the current work and that of Anglada, and the resulting rate coefficients are not expected to be accurate.

Calculated rate coefficients from this work for the OH + formic acid total loss rate coefficients are shown versus the experimental values in Figure 4, and the corresponding calculated branching ratios between the two abstraction sites are shown in Figure 5. We have chosen to present calculated values with two models for treating quantum mechanical tunneling, a WKB method and an asymmetric Eckart barrier. While, in principle, the WKB method gives a more realistic description of the true 1-D tunneling potential, we note that this reaction potential is based on M062X/6-31+G** calculations, and the imaginary frequency used to parametrize the Eckart tunneling potential is calculated at the CCSD­(T)-F12 level. The agreement between experiment and theory is good for both models, although the WKB method underpredicts the experimental rate coefficients at lower temperature. Both models are within a factor of 2 of the experimental rate coefficients over the full temperature range, and considering that no tuning of barrier heights has been carried out, the theoretical description is nearing quantitative accuracy. Clearly, the tunneling method is a source of error, and multidimensional tunneling methods based on anharmonic transition-state frequencies look promising. Additionally, our coupled rotor calculations do not yet account for couplings between torsional angles and other stretching and bending frequencies which is another likely small source of error.

Interestingly, previous calculations by Anglada also agree well with the experimental rate coefficients, despite the lower levels of theory employed. Anglada also employed a steady-state expression when calculating rate coefficients, implying macroscopic and thus high-pressure limiting rate coefficients for the association and dissociation reactions between the reactants and the prereaction complexes. A master equation calculation clearly shows that the complexes have negligible lifetimes and are kinetically irrelevant under the conditions of interest. Our focus is on higher temperatures; low-temperature measurements, which might be expected to show evidence of the role of an initial van der Waals complex, are difficult due to dimerization. Should such data become available, modeling would have to consider the outer bottleneck to complex formation.

Turning to the branching ratios, we find that the channel occurring via the lower energy barrier to form HCO_2_ ([Disp-formula ueq1]) dominates at low temperatures. As the temperature increases, the HOCO-forming channel takes over despite its higher energy barrier, implying a larger *A* factor for this channel. The structures of the two transition states are shown in Figure 7, and the lower-energy TS_HCO2_ is clearly entropically tighter due to the presence of two hydrogen bonds restricting the internal rotation of the OH moiety.

**Figure 7:**
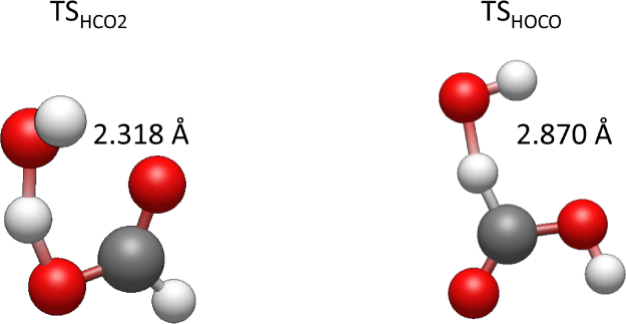
Structures of TS_HCO2_ (left) and TS_HOCO_ (right) as optimized by CCSD­(T)-F12/cc-pVDZ-F12 calculations.

### 3.4 H Atom Yield Production from HOCO Decomposition

As shown in Figure 5, above 600 K, the experimental yield of H atoms increases in contrast to the calculations from this work and Anglada.^19^ Possible sources of H include decomposition of HCO (produced from FA photolysis; see SI, section S5) or HOCO:

R8 HCO→H+CO

R3b $$\mathrm{HOCO}\to {H+\mathrm{CO}}_{2}$$

The H channel for Reaction 1 has been proposed by Yin et al.^31^ ([Disp-formula ueq8]) to explain the faster-than-expected decomposition of FA in their jet-stirred reactor study on the pyrolysis of formic acid at temperatures of 600–1100 K and at 1 bar. We have calculated the barrier for [Disp-formula ueq8] at the ANL level of theory, and the resulting value, 37 kJ mol^–1^, is significantly higher than that calculated by Yin et al. (22.6 kJ mol^–1^) and precludes [Disp-formula ueq8] as a source of enhanced H atoms. HCO is a coproduct of OH in FA photolysis; however, enhanced H atom yields were also observed when 266 nm photolysis of H_2_O_2_ is used as the OH source (green point in Figure 5), and hence, no HCO is photolytically produced. Our proposed source of enhanced H atom production above 600 K is therefore HOCO decomposition.

To help verify our hypothesis on enhanced H atom formation, we initially moved to using Cl + FA ([Disp-formula ueq19]), where the acidic H abstraction to give HCO_2_ + HCl is 37.3 kJ mol^–1^ ^10^ endothermic, to study H atom production at higher temperatures:^10^

R9 $$\mathrm{Cl}+\mathrm{HC}\left(O\right)\mathrm{OH}\to \mathrm{HCl}+\mathrm{HOCO},\Delta_{r}H_{298K}=-19.3{\mathrm{kJ mol}}^{-1}$$

where Cl atoms were generated by the photolysis of oxalyl chloride at 355 nm (no FA photolysis occurs at this wavelength):^51^

P1 $${\left(\mathrm{COCl}\right)}_{2}+h\nu \left(\mathrm{355 nm}\right)\to \mathrm{Cl}+\mathrm{ClCOCO}\to \mathrm{2Cl}+\mathrm{2CO}$$

Figure 8 shows typical H atom traces from reaction [Disp-formula ueq5] at temperatures from 573 to 723 K.

**Figure 8:**
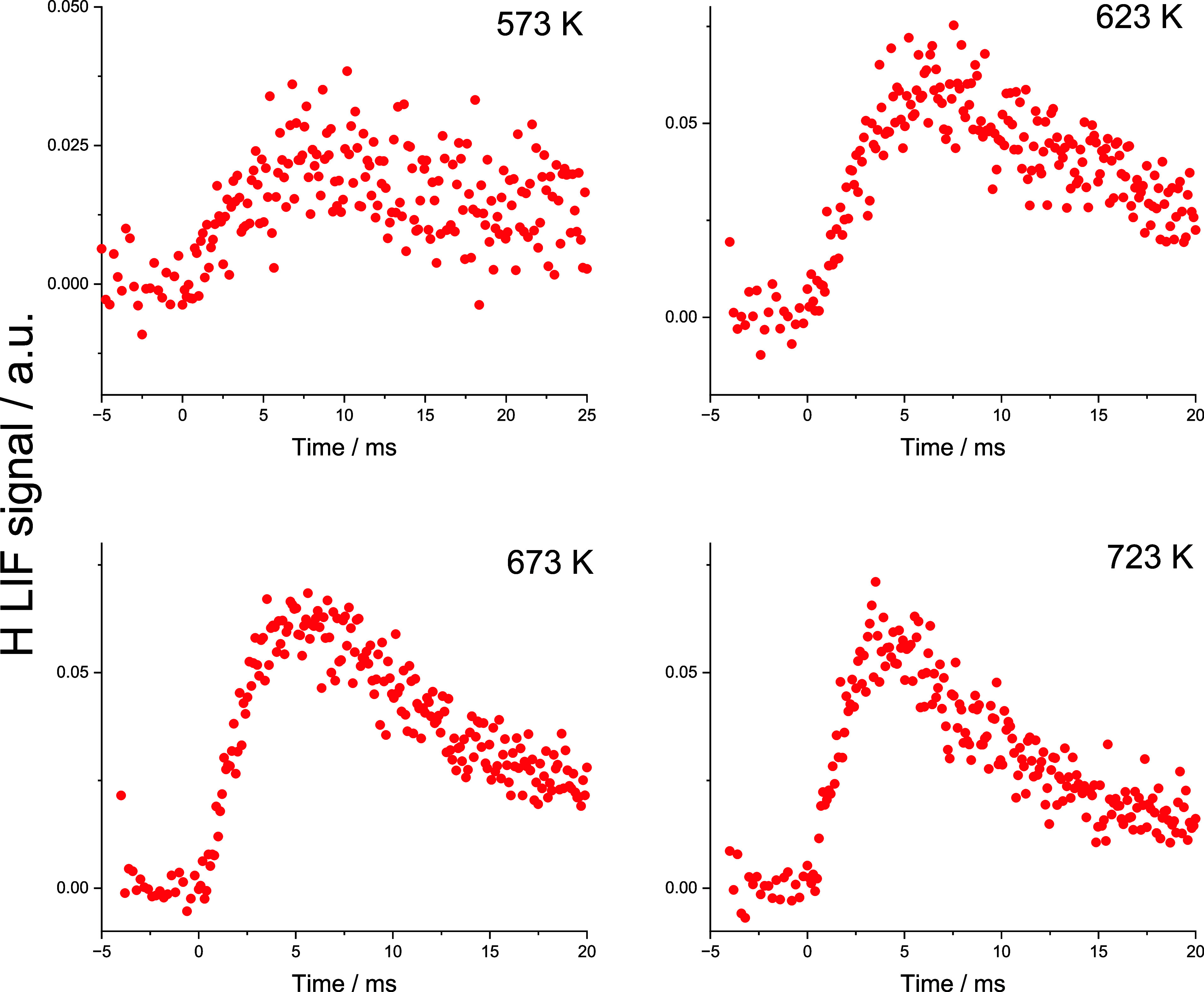
Typical H atoms signals following the photolysis of oxalyl chloride (COCl)_2_ to generate Cl and the fast Cl + HC­(O)­OH reaction to solely generate HOCO ([Disp-formula ueq19]). Experiments were performed at ∼50 Torr Ar. As expected, the H atom signal grows from zero, with a slight induction time.

Analysis of the H atom traces following initiation by [Disp-formula ueq24] is complex due to the multiple steps in Cl production and the difficulty in adding sufficient HC­(O)­OH to make the rate of HOCO generation via [Disp-formula ueq24] significantly greater than that of HOCO decomposition. The resulting values for *k*_3_ are correlated with *k*_9_. However, qualitatively, the traces show H atom growth from a zero background, consistent with no photolytic source or rapid production via HCO_2_ decomposition.

For the majority of experiments on HOCO decomposition, FA photolysis at 266 nm ([Disp-formula ueq26]) was used as the HOCO source (as opposed to FA photolysis at other wavelengths or the Cl + HC­(O)­OH reaction).

P2 HC(O)OH+hν(266 nm)→H+HOCO

[Disp-formula ueq26] promptly generated equal amounts of HOCO and H, and the subsequent growth of H from [Disp-formula ueq5] can be seen in Figure 9. Under these conditions, *k*_3_ can be extracted from the growth of the H atom signal. FA photolysis allows for a greater dynamic range of HOCO decomposition to be monitored. Following FA photolysis at 266 nm, the prompt OH formation signal is very small, as would be expected since the threshold for OH production is 252 nm.^52^ See Figure S5 showing the energetics of FA photolysis. The inset of Figure 9 shows the highly magnified OH signal. Given that we are intrinsically more sensitive to OH than H, by at least a factor of 10, we believe that the observed prompt OH is more than 100 times less than prompt H and probably arises from photolysis of secondary products. A small growth in OH is observed, and at higher temperatures and pressures, this correlates well with H growth, suggesting that this OH does arise from HOCO decomposition. At the highest total pressures, where OH from HOCO decomposition is promoted, the H and OH signals were compared (in He to minimize OH LIF quenching) using the calibration reaction [Disp-formula ueq12]. This allowed yields from R3 to be assigned (with the typical H atom yield being 0.93–0.98), which in turn can be used to test the tunneling description used in our MESMER calculations.

**Figure 9:**
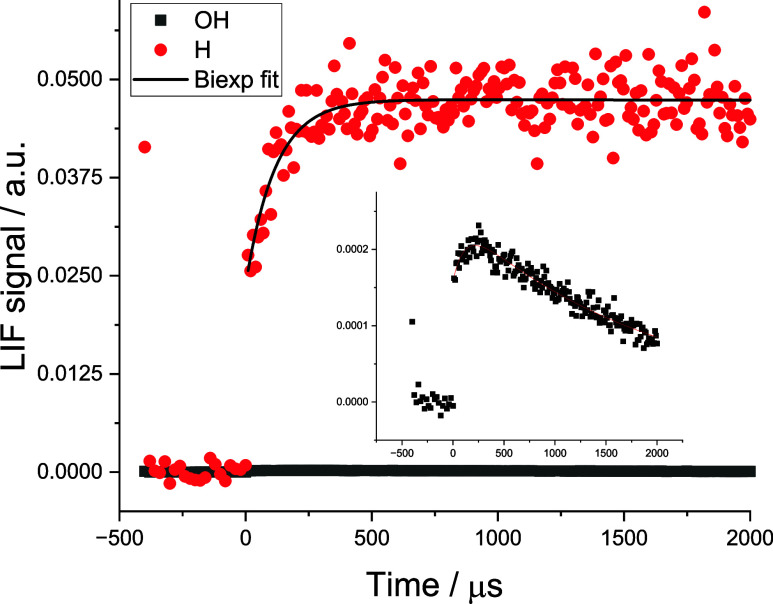
Typical H atom and OH LIF signal following the photolysis of FA at 266 nm at 723 K and ∼ 200 Torr of He. FA photolysis leads to equal amounts of [H]_0_ and [HOCO]_0_. H atom growth from HOCO decomposition can be seen, and the approximate doubling of the H LIF signal demonstrates the reaction [Disp-formula ueq5] is dominant. This is reinforced by the extremely low OH signal shown in the main figure and expanded in the intercept. LIF detection for OH is more sensitive than H; the OH yield in this experiment is equal to 0.06 ± 0.03.

Figure 10 shows *k*_3_ (red dots) as a function of temperature and pressure following HC­(O)­OH photolysis at 266 nm, along with the master equation fits (see below). As would be expected, the rate coefficient for HOCO decomposition increases with both temperature and pressure. The data shown in Figure 10 are tabulated in SI, Table S2.

**Figure 10:**
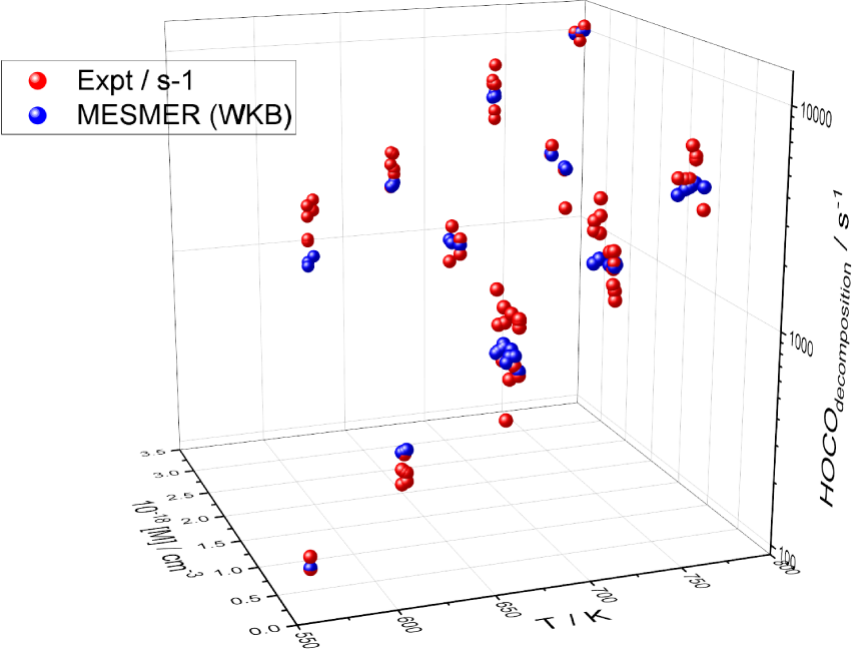
Rate coefficient for HOCO decomposition, *k*_3_, as a function of temperature. Red dots are the experimental data points following FA photolysis at 266 nm to generate HOCO. The blue dots are the results of the MESMER fit.

The potential energy surface for the two HOCO dissociation channels is shown in Figure 11. A previous high-level study characterized the stationary points using the accurate HEAT protocol.^53^ The energy values from this previous study are tabulated alongside those from our current work in Table 2, and excellent agreement is observed.

**Table 2:** Comparison of Energy Values for the Stationary Points on the HOCO Decomposition Potential Energy Surface

Stationary point energy (kJ mol^–1^) at 0 K	This work^a^	Barker et al.^b^	ATcT^c^
TS_OH_	107.1	107.3	
TS_H_	110.0	111.3	
OH + CO	Not calculated	104.1	104.62 ± 0.40
H + CO_2_	5.3	3.7	4.06 ± 0.40

a Calculations at the ANL level.

b Calculations at the HEAT level,^53^ relative to Δ_f_H_0 K_ HOCO.

c ATcT values,^10^ relative to relative to Δ_f_H_0 K_ HOCO = −181.14 ± 0.40 kJ mol^–1^.

**Figure 11:**
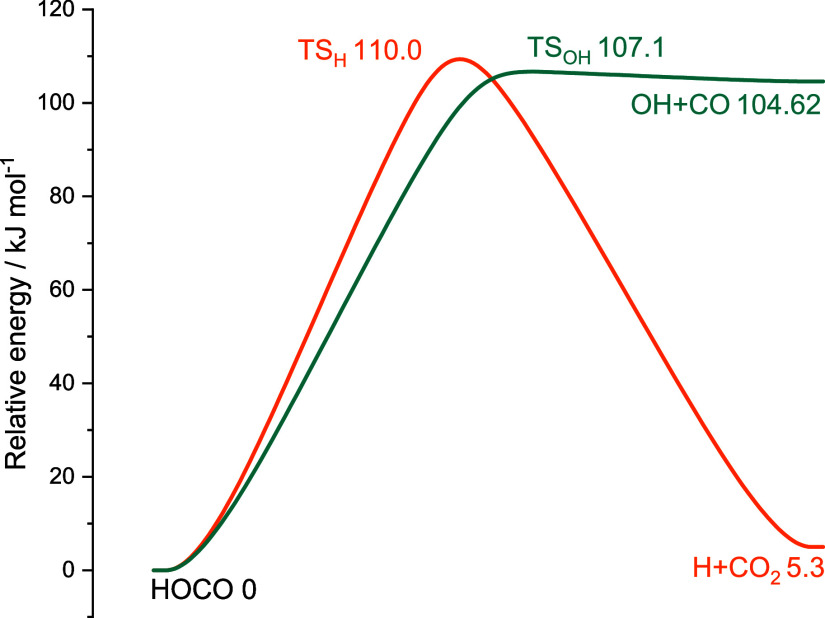
Stationary points for the HOCO decomposition reaction with energy values calculated at the ANL level. The value for the OH + CO channel is from ATcT.^10^

Having obtained a robust PES, MESMER was used to fit the energy transfer parameters of HOCO to the experimental data obtained herein using the WKB tunneling method (see SI, Table S3 for results with Eckart tunneling parameters), and the fitted parameters are given in Table 3 with both Ar and He bath gases. Given the relatively narrow temperature range of the experiments, floating both energy transfer parameters may be overfitting the data (although both are well-defined); with *n* fixed, ⟨Δ*E*_*d*_⟩(298K) is given with high precision, and the Ar value is sensibly greater than that of He. Given that the system is so far into the falloff region from the high-pressure limit, our experiments provide no information on the barrier heights for HOCO decomposition.

**Table 3:** Fitted Energy Transfer Parameters for HOCO in He and Ar Bath Gases

Energy transfer parameters $\langle \Delta E_{d}\rangle \left(T\right)=\langle \Delta E_{d}\rangle \left(298K\right)\times {\left(\frac{T}{298K}\right)}^{n}$	Fitted values using WKB tunneling method Errors statistical at 2σ level
⟨Δ*E*_*d*_⟩(298 K)Ar (cm^–1^)	51 ± 13
*n* Ar	1.06 ± 0.31
⟨Δ*E*_*d*_⟩(298 K)He (cm^–1^)	74 ± 15
*n* He	0.51 ± 0.24
⟨Δ*E*_*d*_⟩(298 K)Ar (cm^–1^)	79 ± 2
*n* Ar	0.5 fixed
⟨Δ*E*_*d*_⟩(298 K)He (cm^–1^)	48 ± 1
*n* He	1.0 fixed

Within the scatter of the experimental data, the calculated rate coefficients agree well with the model. As can be seen in SI, the Eckart model also gives good agreement for *k*_3_, where the two models differ more substantially is in the experimental values for the H atom yields (see Figure 12). Both theoretical models agree that H + CO_2_ is the dominant product under the temperature range of interest here, despite being energetically and entropically unfavorable. The dominance of the H atom channel is entirely due to quantum mechanical tunneling, and previous dynamical studies^54^ have highlighted the efficiency of tunneling in this reaction. Both tunneling methods used here are 1-dimensional and approximate and are thus a significant source of uncertainty in the current calculations. Nevertheless, the calculated yields agree well with the experimental data for the WKB method and qualitatively for Eckart. This agreement confirms the importance of tunneling in promoting H atom formation from HOCO decomposition.

**Figure 12:**
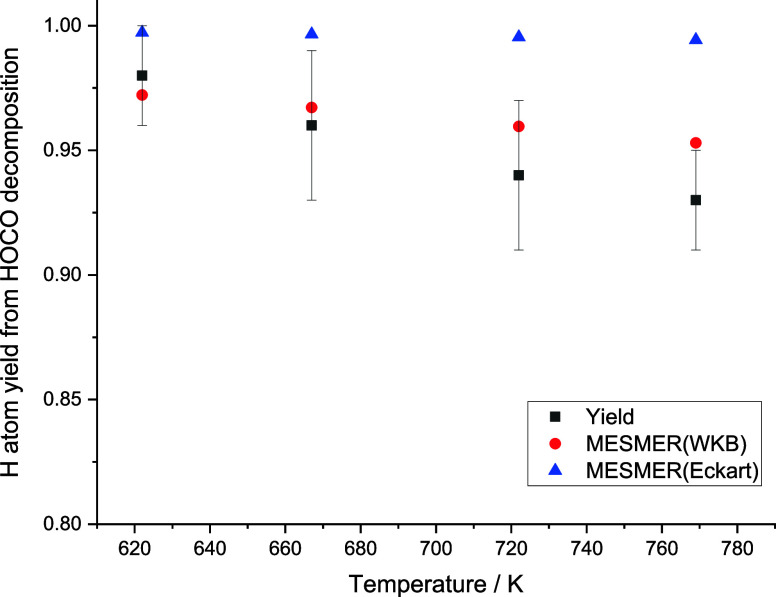
Comparison between theoretical and experimental (black ■) yields of H + CO_2_ from HOCO decomposition. Two theoretical models are used, one with an Eckart tunneling method (blue ▲) and one using a WKB approach (red ●). The error bars shown are statistical errors at the 2σ level from MESMER analysis.

Using our recommended MESMER model (WKB with *T*-dependent energy transfer), simulations have been run over a large range of temperatures (400–1200 K, every 100 K) and pressures (0.001–10000 atm, every order of magnitude) in order to provide a sufficiently large data set to parametrize our results. These data have been parametrized using the approach of Gou et al.,^55^ where for each pressure (in atm), the data are fitted to the equation:

ln(k)=ln⁡A×nln(T)−(Ea/(1.987×T)

The parameters ln*A*, *n*, and *Ea* can be obtained directly from MESMER^44^ but have presently been obtained by nonlinear least-squares fitting to provide easily usable estimates of *k*_3_. The three parameters, as a function of ln­(pressure/atm), can be represented using cubic equations:

$$\ln A=A_{0}+A_{1}\times \ln\left(p\right)+A_{2}\times \ln{\left(p\right)}^{2}+A_{3}\times \ln{\left(p\right)}^{3}$$

$$n=n_{0}+n_{1}\times \ln\left(p\right)+n_{2}\times \ln{\left(p\right)}^{2}+n_{3}\times \ln{\left(p\right)}^{3}$$

$$Ea=Ea_{0}+Ea_{1}\times \ln\left(p\right)+Ea_{2}\times \ln{\left(p\right)}^{2}+Ea_{3}\times \ln{\left(p\right)}^{3}$$

Such an approach provides a simple method to calculate the rate coefficient at any pressure and temperature, *k*(*T*,*p*). The parameters are given in SI (section S8) for HOCO decomposition to both H and OH. It is noted that all of the MESMER rate coefficients are reproduced to within 20% using this parametrization. While this parametrization has not been used before, it might turn out to be a robust and straightforward approach to parametrize the rate coefficients for most systems, even if the master equation describes a complicated potential energy surface.

Barrier heights for the two dissociation channels are quite similar, but the looser transition state leading to OH + CO means that simple calculations ignoring tunneling would predict that dissociation with HO + CO would dominate. For example, Golden et al.^17^ calculate a high-pressure branching ratio to HO + CO of 0.90 at 600 K, increasing to 0.92 at 1000 K. Our master equation simulations show that as both temperature and pressure increase, the yield of OH increases, but OH remains a minor channel (see Figure 12).

Most studies on the HOCO system have focused on the kinetics of the OH + CO reaction ([Disp-formula eq1]) as this is a key process in heat release during combustion and the main route to CO_2_ formation:

R10 $$\mathrm{OH}+\mathrm{CO}\to \mathrm{HOCO}\to H+{\mathrm{CO}}_{2}$$

Recently, studies have recognized that tunneling from HOCO to the H + CO_2_ products is important.^16,53^ However, starting from OH + CO, the HOCO intermediate will be formed with a large excess (∼100 kJ mol^–1^) of internal energy, in contrast to our studies, where HOCO will be formed thermally relaxed, closer to the bottom of the HOCO energy well. High-temperature studies on OH + CO will therefore be less sensitive to the parameters controlling HOCO thermal decomposition.

The potential role of tunneling in the decomposition of HOCO has been suggested by Miyoshi et al.^56^ based on different lifetimes for HOCO depending on the method of generation. When Miyoshi et al. generated HOCO via [Disp-formula ueq24], its lifetime at room temperature, ∼10 ms, was significantly longer than when HOCO was generated via the photolysis of acrylic acid, ∼1.25 ms. The lifetime of HOCO was also dependent on the H_2_ bath gas pressure, increasing from ∼1.25 ms at ∼2 Torr of H_2_ to ∼ 2.5 ms at ∼8 Torr of H_2_. Additionally, DOCO showed a small kinetic isotope effect (0.7 ± 0.2) over the pressure range of 2–11 Torr of H_2_ at room temperature. While there may be some loss of HOCO via Reaction [Disp-formula ueq5] due to energized HOCO generated in the photolysis pulse, the ms time scales of the experiments should have predominantly thermalized the HOCO from either source, and the observed kinetic isotope effect is much smaller than would be expected from the differences in H and D tunneling probabilities. It therefore seems more likely that the decreased lifetime of HOCO following photolytic production observed by Miyoshi et al. is due to secondary chemistry; however, further studies are warranted.

### 3.5 Determination of the Rate Coefficient for HOCO + O_*2*_ at 673–773 K

The production of H atoms from HOCO decomposition above 600 K allows for the determination of the rate coefficient for Reaction 5:

R5 $$\mathrm{HOCO}+O_{2}\to {\mathrm{HO}}_{2}+{\mathrm{CO}}_{2}$$

Adding O_2_ will increase the rate of HOCO removal (and hence H atom production) and decrease the H atom signal due to the competition between R3 and [Disp-formula ueq31]. Figure 13 shows an example of H atom traces in the presence of oxygen. Although the addition of oxygen decreases the signal quality, *k*’_5_ (where *k*’_5_ = *k*_5_[O_2_]) is determined both from the rate of growth of the H atom signal and the final H atom signal level. Hence, the errors on *k*’_5_ are relatively low, and the bimolecular rate coefficient is defined to be ±10% (2σ).

**Figure 13:**
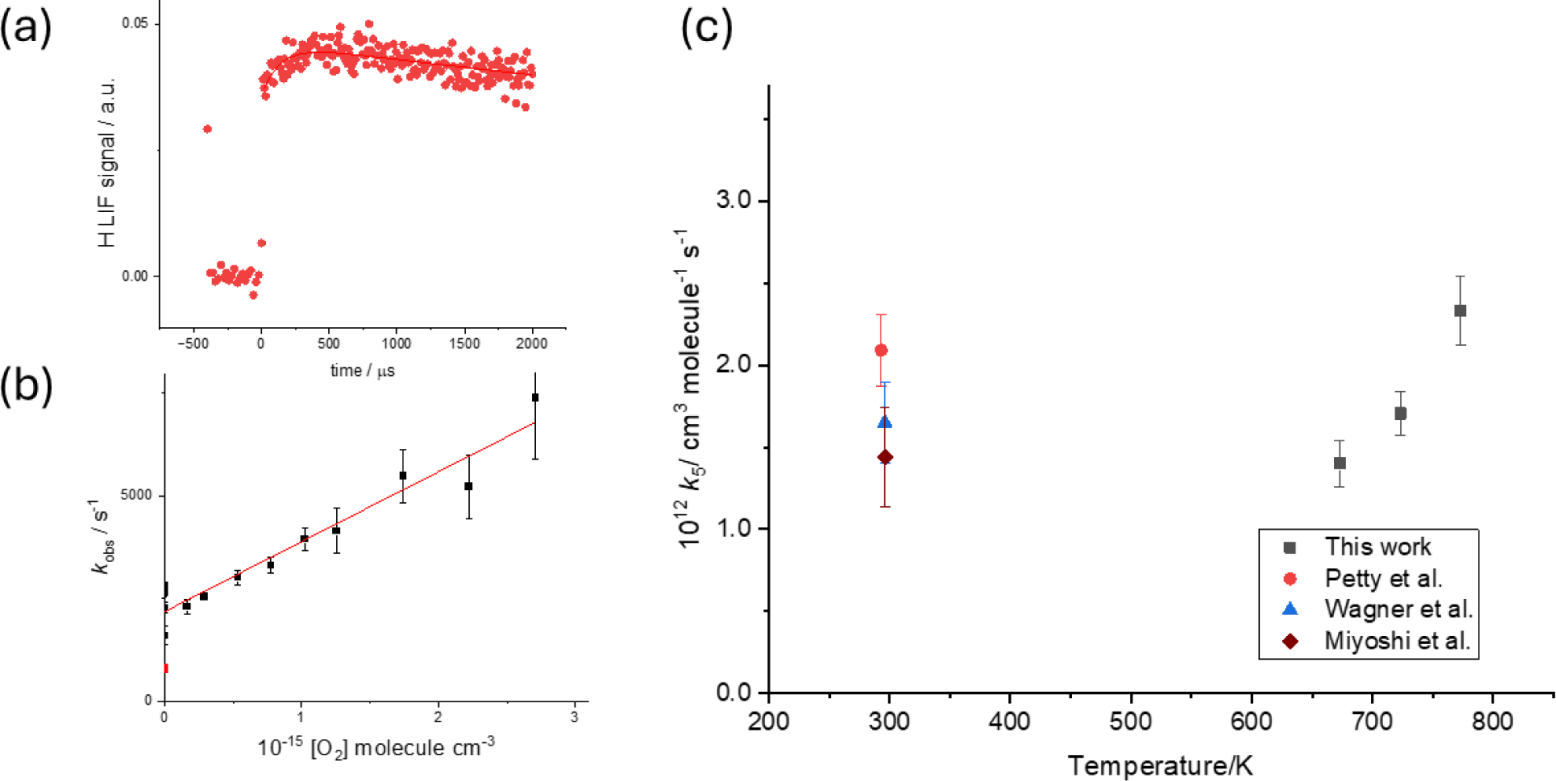
(a) Typical H atom signal, note that unlike Figure 9, the magnitude of the growth from HOCO decomposition is much smaller than from FA photolysis as much of the HOCO reacts with O_2_. This reduction in the signal results in the increasing magnitude of the error bars at high [O_2_] on the bimolecular plot. (b) Bimolecular plot to determine *k*_5_. (c) Values of *k*_5_ as a function of temperature from the room temperature literature studies of Petty et al.^57^ (red ●), Nolte et al.^58^ (blue ▲), Miyoshi et al.^56^ (maroon ⧫), and the experimental values (black ■) from this work.

Three previous experimental studies of *k*_5_ were carried out at room temperature. Petty et al.^57^ (*k*_5_ = (2.0 ± 0.2) × 10^–12^ cm^3^ molecule^–1^ s^–1^) monitored HOCO removal by transient IR absorption spectroscopy following photolysis of acrylic acid. Nolte et al.^58^ (*k*_5_ = (1.65 ± 0.25) × 10^–12^ cm^3^ molecule^–1^ s^–1^) used [Disp-formula ueq19] in a discharge flow tube to generate HOCO and followed its removal by LMR. Finally, Miyoshi et al.^56^ (*k*_5_ = (1.44 ± 0.30) × 10^–12^ cm^3^ molecule^–1^ s^–1^) generated HOCO via [Disp-formula ueq19] and monitored HOCO by photoionization spectroscopy. The room temperature measurements of *k*_5_ are in reasonable agreement, with the error bars of the highest and lowest measurements of *k*_5_ close to overlapping. [Disp-formula ueq31] has also been studied theoretically. Yu and Muckerman^59^ used a direct dynamics approach to calculating *k*_5_; their room temperature rate coefficient (*k*_5_ = 2.1 × 10^–12^ cm^3^ molecule^–1^ s^–1^) is in excellent agreement with experimental results, and they predicted a modest positive temperature dependence, with *k*_5_ rising to 5.79 × 10^–12^ cm^3^ molecule^–1^ s^–1^ at 800 K, significantly higher than the value from our measurements ((1.4 – 2.3) × 10^–12^ cm^3^ molecule^–1^ s^–1^). In contrast, Yin et al.^31^ derived *k*_5_ from a calculated potential energy surface using rate theory and predicted a modest negative temperature dependence, giving *k*_5_ = 5.81 × 10^–12^ cm^3^ molecule^–1^ s^–1^ at 300 K and 4.51 × 10^–12^ cm^3^ molecule^–1^ s^–1^ at 800 K.

The errors shown in our values are statistical only at the 2σ level. Given the overall uncertainties in our measurements, which are more likely to be ±30%, we do not read too much into the slight positive temperature dependence in *k*_5_ observed in this study. One possibility is that *k*_5_ is essentially temperature-independent over the temperature range of 300–800 K, with a value of *k*_5_ = (1.75 ± 0.50) × 10^–12^ cm^3^ molecule^–1^ s^–1^. Such a limited temperature dependence would be broadly consistent with an addition/elimination mechanism. However, Poggi and Francisco^60^ predict that there will be a direct abstraction channel with a barrier that would eventually dominate at sufficiently high temperatures; the contribution of direct abstraction would explain our observed positive temperature dependence for *k*_5_. Studies between 300 and 650 K, as well as above 800 K and over a range of pressures (to identify an addition/elimination process), would be beneficial to determine the mechanism of reaction [Disp-formula ueq31].

### 3.6 Impacts

This work extends the experimental temperature range to 850 K. These are challenging experiments, and we have estimated a combined uncertainty of ±20% in the total rate coefficient between 300 and 600 K, rising to ± 50%. This high uncertainty should be taken into account in modeling studies. As can be seen from Figure 14a, the current data for *k*_1_, even with a 50% uncertainty, significantly constrain *k*_1_ compared to the spread of values that have been used in previous modeling studies.

**Figure 14:**
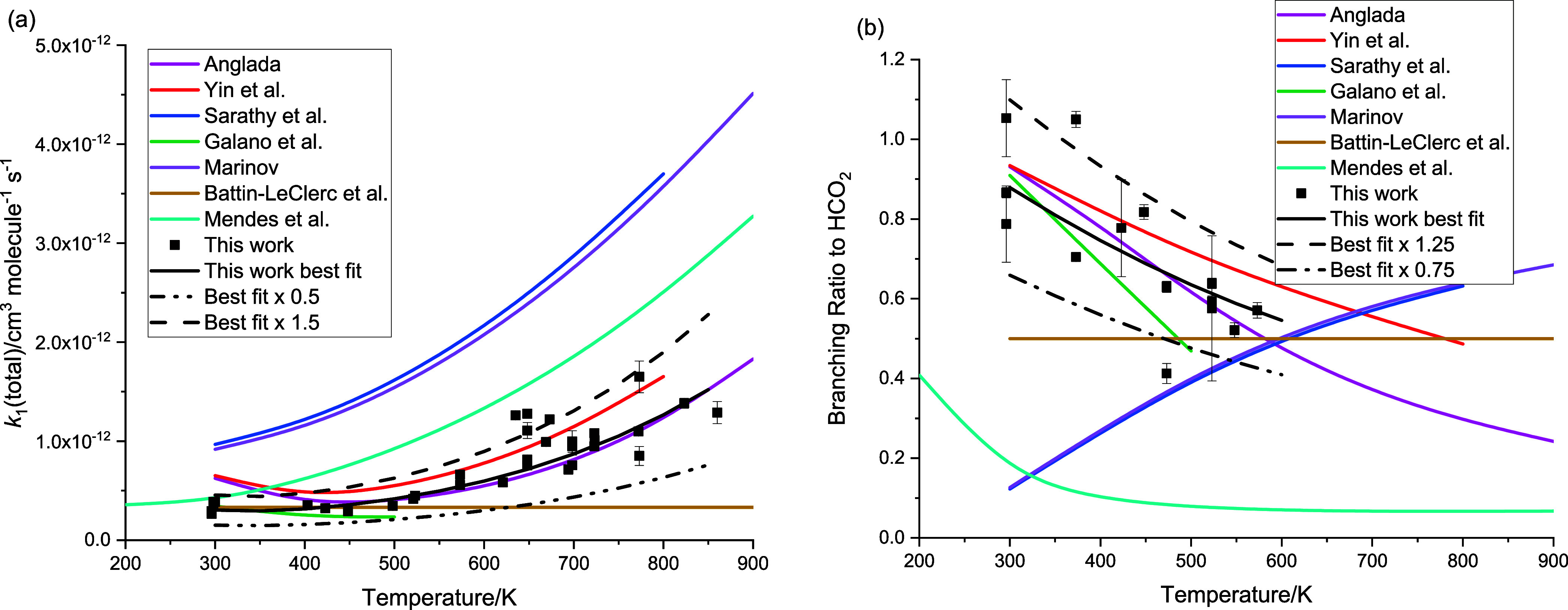
Comparison of our experimental results with values for (a) the total rate coefficient *k*_1_ and (b) the branching ratio to HCO_2_ and hence H.

The overall kinetics and branching ratios calculated by Anglada form the basis of the rate coefficients used in several low-temperature combustion models (e.g., Wako et al.^27^ and Marshall and Glarborg^18^), and the current work validates these values. Other models use values for R1 based on Marinov,^30^ which predict the opposite behavior in the H atom yield as a function of temperature. An early study on formic acid production by Battin-Leclerc et al.^6^ used a temperature-independent value of *k*_1_ = 3.2 × 10^–13^ cm^3^ molecule^–1^ s^–1^ with a temperature-independent 50:50 split between channels forming HOCO and HCO_2_. The experimental and calculated HCO_2_ (and hence H) yields from various studies are shown in Figure 14b.

The stability of formic acid means that unimolecular decomposition of the fuel plays a minor role in fuel consumption compared to that of other oxygenated fuels. Therefore, the branching ratio of R1 is still a key parameter in HC­(O)­OH combustion at higher temperatures than those under typical LTC conditions. Sarathy et al.^4^ state that H atom abstraction reactions by H, OH, and O are the primary routes for formic acid consumption at 1250 K and that Reactions [Disp-formula ueq1] and [Disp-formula ueq2] (and their H and O equivalents) have high and opposite sensitivity coefficients.

Most combustion or pyrolysis models with a focus on FA include the subset of FA chemistry developed by Marshall and Glarborg.^18^ In this submechanism, the kinetics of [Disp-formula ueq5] are based on the study of Golden et al.,^17^ although this reference has been superseded by more recent calculations by Golden, Barker, and coworkers^53^ for OH + CO kinetics. Golden et al.^17^ provide values for *k*_3b_ that are significantly lower than those observed in this work. The commonly used Aramco 2.0 and 3.0 models use the estimates of Marinov^30^ for the kinetics of the OH + FA reactions, and Marinov assumes instant decomposition of HOCO or HCO_2_, so that the products of the OH + FA reaction (R1) in the basic Aramco mechanism are H_2_O + either OH + CO or H + CO_2_. HOCO does arise from other routes in the mechanism, and decomposition parameters are based on calculations from a private communication with John Barker. More recently, Sarathy et al.^4^ have integrated the Marshall and Glarborg FA submechanism into the Aramco model for a study of FA and FA/H_2_ combustion.

Qualitative support for enhanced H atom production comes from the jet-stirred reactor studies of Yin et al.,^31^ carried out at 1 bar, who proposed the alternative channel [Disp-formula ueq8] to provide the additional H atoms needed to bridge the gap between experiment and model for their pyrolysis studies. However, the barrier to forming H + (HO)_2_CO is too high, and H atoms from [Disp-formula ueq5] can provide the required enhancement in reactivity. Additionally, Lavadera and Konnov^28^ increased *k*_3b_ by a factor of 2 from the Golden et al.^17^ value in their experimental and modeling study of HC­(O)­OH/CH_4_ flames. The importance of HOCO chemistry in formic acid oxidation has also been highlighted by Sarathy et al.^4^ Nilsson and Konnov^61^ have carried out a careful review of HOCO chemistry, recommending data from Larson et al.^62^ for *k*_3b_ and from Yu and Muckerman^59^ for *k*_5_. They applied their revised model to syngas combustion and found little difference in the results with or without the HOCO module, emphasizing that HOCO chemistry will predominantly be of interest when there is a significant channel for generating thermal HOCO, i.e., via [Disp-formula ueq2].

## 4 Summary

The overall rate coefficient for R1, *k*_1_, is in good agreement with previous experimental literature over the common temperature range (300 ∼ 450 K). The results confirm the slow rate coefficient at room temperature (*k*_1_ = (3.44 ± 0.48) × 10^–13^ cm^3^ molecule^1^ s^–1^) associated with complex formation and a much tighter transition state required to move forward to either set of products, compared to redissociation. The measured results are in good agreement with the overall kinetics calculated in this work and by Anglada.^19^

Figure 5 also shows good agreement for the H atom yield with the calculations from our work and those of Anglada up to 600 K. Channel [Disp-formula ueq1], abstraction at the acid site, is favored at low temperatures due to a lower barrier and possibly some tunneling, with channel [Disp-formula ueq2] becoming more important as the temperature increases. Above 600 K, we observe an increase in H atom yield, but we believe this is due to HOCO decomposition. It is difficult to extract the primary H atom yield from R1 above 600 K, but our measurements are consistent with the calculations of Anglada, and we therefore recommend the parameters for R1 based on calculations.

Our results on HOCO decomposition suggest that Reaction [Disp-formula ueq23] is the source of the observed increase in H atom yield above 600 K. This observation of H from HOCO decomposition contrasts with some previous studies on the OH + CO/H + CO_2_ system; however, these studies would be insensitive to HOCO thermal decomposition under low-temperature combustion conditions. Our calculations confirm the importance of tunneling in H atom production from [Disp-formula ueq23].^16,53,54^ The experimental values can be fit using the master equation code MESMER.^44^ In this fitting process, the barriers for HOCO decomposition are constrained to their calculated values, the WKB tunneling parameterization is applied, and Δ*E*_*d*_ was adjusted to fit the experimental data.

Our current results show that HOCO → H + CO_2_ needs to be considered when modeling formic acid oxidation under LTC conditions, particularly for pyrolysis at relatively low pressures. At higher temperatures and high pressures, HOCO decomposition to OH + CO becomes dominant.

## Supplementary Material







## Notes

An extended description of the apparatus (S1), data on the conditions for dimerization of formic acid (S2), details on H atom yield analysis (S3), details on the variance of *k*_1,298 K_ (S4), a discussion on formic acid photochemistry (S5), a tabulation of HOCO decomposition data (S6), a summary of energy transfer parameters with Eckart tunneling correction (S7), PLOG outputs for H atom yields as a function of temperature and pressure (S8), vibrational frequencies and rotational constants (S9) (PDF)
MESMER file for HOCO decomposition (TXT)
MESMER file for OH + HC­(O)­OH reaction (PDF)

## References

[1] Millet D. B., Baasandorj M., Farmer D. K., Thornton J. A., Baumann K., Brophy P., Chaliyakunnel S., de Gouw J. A., Graus M., Hu L. (2015). A Large and Ubiquitous Source of Atmospheric Formic
Acid. Atmos. Chem. Phys..

[2] Paulot F., Wunch D., Crounse J. D., Toon G. C., Millet D. B., DeCarlo P. F., Vigouroux C., Deutscher N. M., Abad G. G., Notholt J. (2011). Importance
of Secondary
Sources in the Atmospheric Budgets of Formic and Acetic Acids. Atmos. Chem. Phys..

[3] Eppinger J., Huang K. W. (2017). Formic Acid as a Hydrogen Energy
Carrier. ACS Energy Lett..

[4] Sarathy S. M., Brequigny P., Katoch A., Elbaz A. M., Roberts W. L., Dibble R. W., Foucher F. (2020). Laminar Burning Velocities and Kinetic
Modeling of a Renewable E-Fuel: Formic Acid and Its Mixtures with
H_2_ and CO_2_. Energy Fuels..

[5] Alfazazi A., Li J. J., Xu C. C., Es-Sebbar E. T., Zhang X. Y., Abdullah M., Younes M., Sarathy S. M., Dally B. (2024). Effects of N-Decane Substitution on Structure and Extinction Limits
of Formic Acid Diffusion Flames. Fuel.

[6] Battin-Leclerc F., Konnov A. A., Jaffrezo J. L., Legrand M. (2007). To Better Understand
the Formation of Short-Chain Acids in Combustion Systems. Combust. Sci. Technol..

[7] Popolan-Vaida D. M., Eskola A. J., Rotavera B., Lockyear J. F., Wang Z., Sarathy S. M., Caravan R. L., Zádor J., Sheps L., Lucassen A. (2022). Formation
of Organic
Acids and Carbonyl Compounds in N-Butane Oxidation Via Γ-Ketohydroperoxide
Decomposition. Angew. Chem. Int. Ed..

[8] Yokelson R. J., Griffith D. W. T., Ward D. E. (1996). Open-Path
Fourier Transform Infrared
Studies of Large-Scale Laboratory Biomass Fires. J. Geophys. Res.: Atmos..

[9] Permar W., Wielgasz C., Jin L. X., Chen X., Coggon M. M., Garofalo L. A., Gkatzelis G. I., Ketcherside D., Millet D. B., Palm B. B. (2023). Assessing
Formic and
Acetic Acid Emissions and Chemistry in Western Us Wildfire Smoke:
Implications for Atmospheric Modeling. Environ.
Sci.: Atmos..

[10] Klippenstein S. J., Harding L. B., Ruscic B. (2017). Ab Initio Computations and Active
Thermochemical Tables Hand in Hand: Heats of Formation of Core Combustion
Species. J. Phys. Chem. A.

[11] Atkinson R., Baulch D. L., Cox R. A., Crowley J. N., Hampson R. F., Hynes R. G., Jenkin M. E., Rossi M. J., Troe J. (2006). Evaluated
Kinetic and Photochemical Data for Atmospheric Chemistry: Volume II
- Gas Phase Reactions of Organic Species. Atmos.
Chem. Phys..

[12] Wine P. H., Astalos R. J., Mauldin R. L. (1985). Kinetic and Mechanistic Study of
the OH+HCOOH Reaction. J. Phys. Chem..

[13] Jolly G. S., McKenney D. J., Singleton D. L., Paraskevopoulos G., Bossard A. R. (1986). Rates of OH Radical Reactions.14. Rate-Constant and
Mechanism for the Reaction of Hydroxyl Radical with Formic Acid. J. Phys. Chem..

[14] Singleton D. L., Paraskevopoulos G., Irwin R. S., Jolly G. S., McKenney D. J. (1988). Rates of
OH Radical Reactions.17. Rate and Mechanism of the Reaction of Hydroxyl
Radicals with Formic and Deuteriated Formic Acids. J. Am. Chem. Soc..

[15] Dagaut P., Wallington T. J., Liu R. Z., Kurylo M. J. (1988). The Gas-Phase Reactions
of Hydroxyl Radicals with a Series of Carboxylic-Acids over the Temperature
Range 240–440 K. Int. J. Chem. Kinet..

[16] Nguyen T. L., Xue B. C., Weston R. E., Barker J. R., Stanton J. F. (2012). Reaction
of HO with CO: Tunneling Is Indeed Important. J. Phys. Chem. Lett..

[17] Golden D. M., Smith G. P., McEwen A. B., Yu C. L., Eiteneer B., Frenklach M., Vaghjiani G. L., Ravishankara A. R., Tully F. P. (1998). OH­(OD)+CO: Measurements
and an Optimized RRKM Fit. J. Phys. Chem. A.

[18] Marshall P., Glarborg P. (2015). Ab Initio and Kinetic
Modeling Studies of Formic Acid
Oxidation. Proc. Combustion. Inst..

[19] Anglada J. M. (2004). Complex
Mechanism of the Gas Phase Reaction between Formic Acid and Hydroxyl
Radical. Proton Coupled Electron Transfer Versus Radical Hydrogen
Abstraction Mechanisms. J. Am. Chem. Soc..

[20] Galano A., Alvarez-Idaboy J. R., Ruiz-Santoyo M. E., Vivier-Bunge A. (2002). Rate Coefficient
and Mechanism of the Gas Phase OH Hydrogen Abstraction Reaction from
Formic Acid: A Quantum Mechanical Approach. J. Phys. Chem. A.

[21] Sun W., Saeys M. (2008). First Principles Study
of the Reaction of Formic and Acetic Acids
with Hydroxyl Radicals. J. Phys. Chem. A.

[22] Elm J., Bilde M., Mikkelsen K. V. (2013). Assessment
of Binding Energies of
Atmospherically Relevant Clusters. Phys. Chem.
Chem. Phys..

[23] Mendes J., Zhou C. W., Curran H. J. (2014). Theoretical Chemical Kinetic Study
of the H-Atom Abstraction Reactions from Aldehydes and Acids by H
Atoms and OH, HO_2_, and CH_3_ Radicals. J. Phys. Chem. A.

[24] Anglada J. M., Gonzalez J. (2009). Different Catalytic
Effects of a Single Water Molecule:
The Gas-Phase Reaction of Formic Acid with Hydroxyl Radical in Water
Vapor. ChemPhyschem.

[25] Iuga C., Raul alvarez-Idaboy J., Vivier-Bunge A. (2011). Mechanism and Kinetics of the Water-Assisted
Formic Acid + OH Reaction under Tropospheric Conditions. J. Phys. Chem. A.

[26] Luo Y., Maeda S., Ohno K. (2009). Water-Catalyzed Gas-Phase Reaction
of Formic Acid with Hydroxyl Radical: A Computational Investigation. Chem. Phys. Lett..

[27] Wako F. M., Pio G., Salzano E. (2022). Modeling Formic
Acid Combustion. Energy Fuels.

[28] Lavadera M. L., Konnov A. A. (2021). Laminar Burning Velocities of Methane Plus Formic Acid
Plus Air Flames: Experimental and Modeling Study. Combust. Flame..

[29] Fischer S. L., Dryer F. L., Curran H. J. (2000). The Reaction
Kinetics of Dimethyl
Ether. I: High-Temperature Pyrolysis and Oxidation in Flow Reactors. Int. J. Chem. Kinet..

[30] Marinov N. M. (1999). A Detailed
Chemical Kinetic Model for High Temperature Ethanol Oxidation. Int. J. Chem. Kinet..

[31] Yin G. Y., Xu J. W., Hu E. J., Gao Q. F., Zhan H. C., Huang Z. H. (2021). Experimental and
Kinetic Study on the Low Temperature
Oxidation and Pyrolysis of Formic Acid in a Jet-Stirred Reactor. Combust. Flame..

[32] Glowacki D. R., Lockhart J., Blitz M. A., Klippenstein S. J., Pilling M. J., Robertson S. H., Seakins P. W. (2012). Interception of
Excited Vibrational Quantum States by O_2_ in Atmospheric
Association Reactions. Science.

[33] Onel L., Blitz M. A., Seakins P. W. (2012). A Laser Flash Photolysis, Laser Induced
Fluorescence Determination of the Rate Coefficient for the Reaction
of OH Radicals with Monoethanol Amine (MEA) from 296 - 510 K. J. Phys. Chem. Lett..

[34] Gannon K. L., Blitz M. A., Liang C. H., Pilling M. J., Seakins P. W., Glowacki D. R., Harvey J. N. (2010). An Experimental and Theoretical Investigation
of the Competition between Chemical Reaction and Relaxation for the
Reactions of ^1^CH_2_ with Acetylene and Ethene:
Implications for the Chemistry of the Giant Planets. Faraday Discuss..

[35] McKee K., Blitz M. A., Hughes K. J., Pilling M. J., Qian H. B., Taylor A., Seakins P. W. (2003). H Atom Branching Ratios from the
Reactions of CH with C_2_H_2_C_2_H_4_, C_2_H_6_, and Neo-C_5_H_12_ at Room Temperature and 25, T.H Atom Branching Ratios from the Reactions
of CH with C_2_H_2_C_2_H_4_, C_2_H_6_, and Neo-C_5_H_12_ at Room
Temperature and 25. J. Phys. Chem. A.

[36] Randi P. A.
S., Pastega D. F., Bettega M. H. F., Jones N. C., Hoffmann S. V., Eden S., Barbosa A. S., Limao-Vieira P. (2023). Electronically
Excited States of Formic Acid Investigated by Theoretical and Experimental
Methods. Spectrochim. Acta, Part A.

[37] Choi N., Blitz M. A., McKee K. W., Pilling M. J., Seakins P. W. (2004). H Atom
Branching Ratios from the Reactions of CN Radicals with C_2_H_2_ and C_2_H_4_. Chem. Phys. Lett..

[38] Robertson N. C. K., Onel L., Blitz M. A., Shannon R., Stone D., Seakins P. W., Robertson S. H., Kuhn C., Pazdera T. M., Olzmann M. (2024). Temperature-Dependent,
Site-Specific Rate Coefficients
for the Reaction of OH (OD) with Methyl Formate Isotopologues Via
Experimental and Theoretical Studies. J. Phys.
Chem. A.

[39] Frisch, M. J. ; Trucks, G. W. ; Schlegel, H. B. ; Scuseria, G. E. ; Robb, M. A. ; Cheeseman, J. R. ; Scalmani, G. ; Barone, V. ; Petersson, G. A. ; Nakatsuji, H. , Gaussian 09, Revision A.02, Wallingford CT, Gaussian, 2009.

[40] Kallay M., Nagy P. R., Mester D., Rolik Z., Samu G., Csontos J., Csoka J., Szabo P. B., Gyevi-Nagy L., Hegely B. (2020). The MRCC
Program System: Accurate Quantum Chemistry
from Water to Proteins. J. Chem. Phys..

[41] Werner H. J., Knowles P. J., Manby F. R., Black J. A., Doll K., Hesselmann A., Kats D., Köhn A., Korona T., Kreplin D. A. (2020). The Molpro Quantum Chemistry
Package. J. Chem. Phys..

[42] Adler T. B., Knizia G., Werner H. J. (2007). A Simple and Efficient Ccsd­(T)-F12
Approximation. J. Chem. Phys..

[43] Shannon R. J., Martínez-Núñez E., Shalashilin D. V., Glowacki D. R. (2021). Chemdyme: Kinetically Steered, Automated Mechanism
Generation through Combined Molecular Dynamics and Master Equation
Calculations. J. Chem. Theory Comput..

[44] Glowacki D. R., Liang C. H., Morley C., Pilling M. J., Robertson S. H. (2012). MESMER:
An Open-Source Master Equation Solver for Multi-Energy Well Reactions. J. Phys. Chem. A.

[45] Zetzsch C., Stuhl F. (1982). Rate constants for
reactions of OH with carbonic acids. Physical
Chemical Behaviour Atmos. Pollut..

[46] Antonov I., Voronova K., Chen M. W., Sztáray B., Hemberger P., Bodi A., Osborn D. L., Sheps L. (2019). To Boldly
Look Where No One Has Looked Before: Identifying the Primary Photoproducts
of Acetylacetone. J. Phys. Chem. A.

[47] Carr S. A., Baeza-Romero M. T., Blitz M. A., Price B. J. S., Seakins P. W. (2008). Ketone
Photolysis in the Presence of Oxygen: A Useful Source of OH for Flash
Photolysis Kinetics Experiments. Int. J. Chem.
Kinet..

[48] Karunanandan R., Holscher D., Dillon T. J., Horowitz A., Crowley J. N., Vereecken L., Peeters J. (2007). Reaction of HO with Glycolaldehyde,
Hoch_2_cho: Rate Coefficients (240–362 K) and Mechanism. J. Phys. Chem. A.

[49] Shannon R. J., Blitz M. A., Seakins P. W. (2024). Solving
the OH + Glyoxal Problem:
A Complete Theoretical Description of Post-Transition-State Energy
Deposition in Activated Systems. J. Phys. Chem.
A.

[50] Sun W., Yang L., Yu L., Saeys M. (2009). Ab Initio Reaction
Path Analysis for the Initial Hydrogen Abstraction from Organic Acids
by Hydroxyl Radicals. J. Phys. Chem. A.

[51] Ghosh B., Papanastasiou D. K., Burkholder J. B. (2012). Oxalyl Chloride, ClC­(O)­C­(O)­Cl: UV/VIS
Spectrum and Cl Atom Photolysis Quantum Yields at 193, 248, and 351
nm. J. Chem. Phys..

[52] Brouard M., Wang J. X. (1992). Photophysics of HCOOH­(a) Close to Its Electronic Origin. J. Chem. Soc., Faraday Trans..

[53] Barker J. R., Stanton J. F., Nguyen T. L. (2020). Semiclassical
Transition State Theory/Master
Equation Kinetics of HO Plus CO: Performance Evaluation. Int. J. Chem. Kinet..

[54] Ma D. D., Ma J. Y. (2022). Full-Dimensional
Quantum Mechanical Calculations for the Tunneling
Behavior of HOCO Dissociation to H + CO_2_. Phys. Chem. Chem. Phys..

[55] Gou, X. ; Miller, J. A. ; Sun, W. ; Ju, Y. G. Implementation of Plog Function in Chemkin II and III. https://engine.princeton,edu/model-reduction/ (accessed 1st June 2025).

[56] Miyoshi A., Matsui H., Washida N. (1994). Detection
and Reactions of the HOCO
Radical in Gas Phase. J. Chem. Phys..

[57] Petty J. T., Harrison J. A., Moore C. B. (1993). Reactions
of Trans-HOCO Studied by
Infrared-Spectroscopy. J. Phys. Chem..

[58] Nolte J., Grussdorf J., Temps E., Wagner H. G. (1993). Kinetics of the
Reaction HOCO+O_2_ in the Gas Phase. Z. fur Naturforsch. - J. Phys. Sci..

[59] Yu H. G., Muckerman J. T. (2006). Quantum
Molecular Dynamics Study of the Reaction of
O_2_ with HOCO. J. Phys. Chem. A.

[60] Poggi G., Francisco J. S. (2004). An Ab Initio
Study of the Competing Reaction Channels
in the Reaction of HOCO Radicals with NO and O_2_. J. Chem. Phys..

[61] Nilsson E. J. K., Konnov A. A. (2016). Role of HOCO Chemistry
in Syngas Combustion. Energy Fuels.

[62] Larson C. W., Stewart P. H., Golden D. M. (1988). Pressure and Temperature-Dependence
of Reactions Proceeding Via a Bound Complex - an Approach for Combustion
and Atmospheric Chemistry Modelers - Application to HO+CO –
> HOCO – > H+CO_2_. Int.
J.
Chem. Kinet..

