# Supplementary material for: Rate Coefficients and Branching Ratios for the Reaction of the OH Radical with Formic Acid under Low-Temperature Combustion Conditions and the Fate of the HOCO Product
Source: J Phys Chem A. 2025 Jul 2;129(28):6437–50. doi: 10.1021/acs.jpca.5c01814 (PMC12278225; doi:10.1021/acs.jpca.5c01814)
Supplement: Supplementary file 1 [file jp5c01814_si_001.pdf]

Rate Coefficients and Branching Ratios for the Reaction of the OH Radical with Formic Acid under Low Temperature Combustion Conditions and the Fate of the HOCO Product

Supplementary Information

Mark A. Blitz[1,2]*, Poppy Guy[1], Robin Shannon[1] and Paul W. Seakins[1]*

1 - University of Leeds, Leeds, LS2 9JT, UK

2 - National Centre for Atmospheric Science (NCAS), University of Leeds, Leeds. LS2 9JT, UK

Contents

S1. Extended description of apparatus set up for simultaneous determination of H and OH kinetics

S2. Dimerization of formic acid

S3. Further details on H atom yield determination

S4. Variation in room temperature rate coefficients and tabulation of data

S5. Formic acid photolysis

S6. HOCO decomposition rate coefficients

S7. Energy transfer parameters

S8. Plog parameterization of HOCO decomposition

S9. Vibrational frequencies and rotational constants from ab initio calculations

Sample MESMER Input Files are also available in the SI

S1. Apparatus setup

Figure S1 shows a schematic of the apparatus used in this study. The two conventional slow-flow, flash photolysis cells, as used in previous studies,[1, 2] are coupled in series via ¼" tubing. The photolysis laser beam passes through both cells and the laser fluence can be measured after each cell. Both cells could be heated and typically the temperature difference between cells was less than 3 K. Gas flows typically entered reactor 1 (H atom detection and monitoring [HCOOH]) first; this means that gases entering reactor 2 (OH detection) could be contaminated by low concentrations of products from reactor 1. However, the gas flow could be revered to check for any effects and no significant effects were observed.

In addition to monitoring [HCOOH] directly via in situ VUV absorption, based on the absorption cross section at 121.6 nm ((1.32 ± 0.07) × 10^{-17} cm^2 $molecule^{-1}$)[3], [HCOOH] could also be monitored in a conventional 1 m long absorption cell (not shown in schematic) using the mercury line at 185 nm. The absorption cross section at 185 nm is significantly smaller, so the absorbance is not large; however, the values of [HCOOH] were consistent with those from the VUV absorbance and those calculated from the bubbler. Absorption at 185 nm was only used infrequently to double check on [HCOOH]. Given the difficulties of working with FA, due to its low vapour pressure, it is important to have a range of measurement techniques.

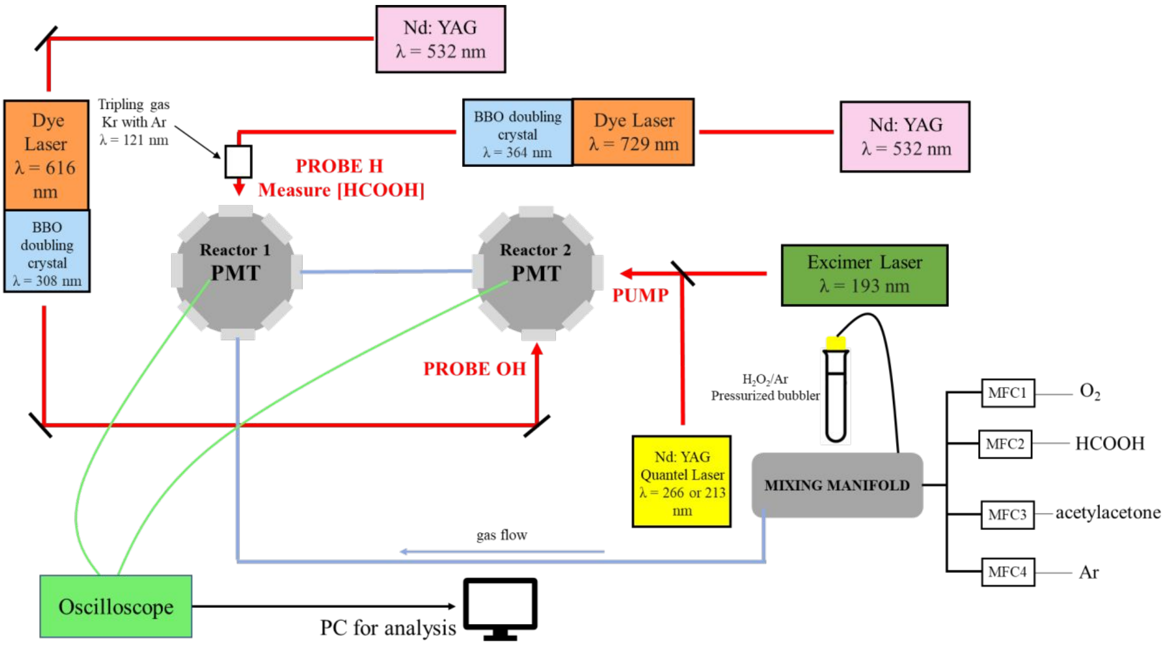

Figure S1. Schematic of apparatus. Beyond Reactor 1 was a VUV detector that could monitor the absorption from the formic acid as its concentration was changed between experiments.

S2. Dimerization of formic acid

Dimerization of formic acid is a potential complication in these studies. We are able to use the VUV light as an in situ absorption probe for [HCOOH]. At higher concentrations (Figure S2) one starts to see a significant deviation in the ratio of the calculated vs expected absorbance as dimerization occurs. Experiments close to room temperature used [HCOOH] < 3×10^{15} molecule cm^{-3}. Dimerization is less of a problem above 400 K.

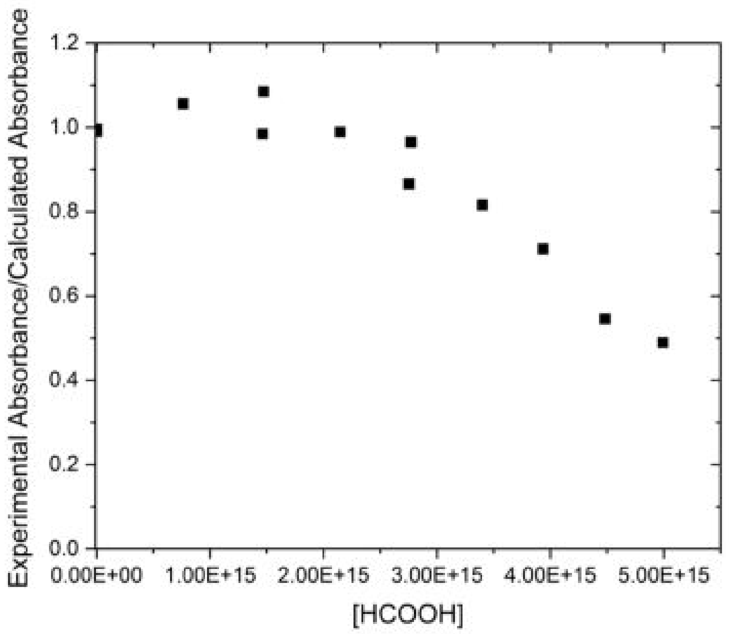

Figure S2. Onset of HCOOH dimerization at room temperature for [HCOOH] > 3×10^{15} molecule cm^{-3}.

S3. Determination of H atom yield

Figure S3 shows our approach to determining the H atom yield from Reaction (1), where reaction numbers in the SI refer to those in the main text. The black points represent the H atom signal, normalized for variations in VUV probe intensity, from formic acid photolysis at 213 nm. At this wavelength, the photolysis process is dominated by production of HCO + OH (see below) with HCO rapidly dissociating to H + CO. The H atom signal is fitted to the following equation:

$$[\mathrm{H}] = C * \left(\frac{k_{\mathrm{reaction}}}{k_{\mathrm{reaction}} - k_{\mathrm{diff}}}\left[e^{-k_{\mathrm{diff}}t} - e^{-k_{\mathrm{reaction}}t}\right]\right) + P * e^{-k_{\mathrm{diff}}t} \qquad \text{ES1}$$

where C is the signal associated with growth via reaction (either from R1 or R6) and is proportional to the initial OH, $[OH]_0$, k_{reaction} is the pseudo-first-order rate coefficient for the

reaction of OH with either HCOOH (R1) or OH with H_2 and HCOOH, k_{diff} is the diffusional rate coefficient for H, approximated to a first order loss process and P is the H signal associated with photolysis.

The reported values shown in Figure 5 are from analysis where all the traces recorded at a given temperature were fitted simultaneously, global analysis. Here, typically five formic acid concentrations were used, where for a given formic acid concentration an additional trace is recorded with an excess of added H_2, so that the majority of the OH is reacting with H_2. The overall kinetics scheme is:

$OH + HC(O)OH \rightarrow H_2O + HCO_2 \quad \rightarrow \quad H + H_2O$ and CO_2 R1a, $k_{1a,bim}$

$OH + HC(O)OH \rightarrow H_2O + HOCO$ R1b, $k_{1b,bim}$

Therefore, the H yield is given by k_{1a} / k_1 (=H_{yield}). When H_2 is added OH is also removed via the well-known reaction:

$OH + H_2 \rightarrow H_2O + H$ R6, $k_{6,bim}$

Under all conditions there was no evidence for chemical removal of hydrogen atoms, so it was reasonably approximated as a slow loss:

$H \rightarrow$ loss Rloss, k_{diff}

Reactions R1 and R2 are pseudo-first-order, and together with Rloss represent coupled first order differential equations (ODE). Rearranging and eliminating leads to a second order ODE in terms of H. Solving this leads to an analytical solution that needs to be solved for the initial condition, $[OH]_0$ and $[H]_0$. The photolysis of HCOOH at 213 nm forms both OH and H:

HCOOH + 213 nm $\rightarrow$ H + OH + other (HCO) $P_{HCOOH,213nm}$

The overall solution is equation ES1, where $k_{reaction} = k_{1bim}[HC(O)OH] + k_6[H_2]$ and $C \propto [OH]_0 \times H_{yield}$ and $P \propto [H]_0$. In the analysis, the pair of traces for each formic acid is assigned a $[OH]_0$ and the $[H]_0$ is assigned via $[OH]_0 \times H_{ratio}$, where H_{ratio} recognises that the H:OH ratio from $P_{HCOOH,213nm}$ is constant at a given T. Overall, the adjusted parameters in the analysis were: $[OH]_0$, H_{ratio}, $k_{1,bim}$, $k_{6,bim}$, H_{yield} and k_{diff} and the fixed parameters were [HC(O)OH] and $[H_2]$. This global analysis was carried out using the software package ORIGIN, where close attention was paid to the fits to the data.

The yield can be gauged by the enhancement in the H signal height when H_2 is added, and in general the increase is not great and implies the reaction R1a is dominant, see Figure S3.

The red dots show the H atom signal when a large concentration of H_2 is added, sufficient to intercept more than 98% of the initial OH. Due to the large excess of H_2, the growth in H atom signal from reaction is much more rapid and diffusional loss makes a much small contribution. The yield of H from R1 is given by the ratio of the C factors from the two traces.

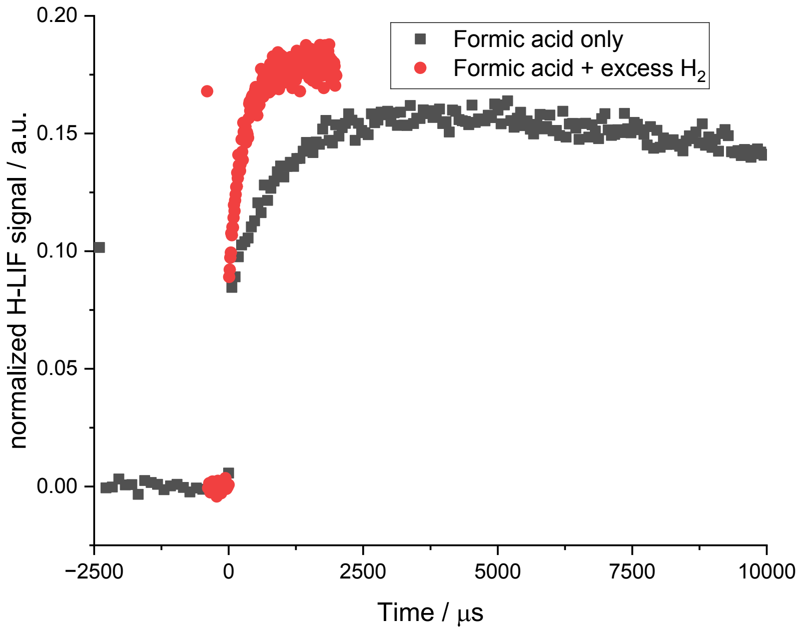

Figure S3. Typical experimental traces used to determine the H atom yield from R1. (■) data points from photolysis of formic acid only at 213, (●) data points with the same [HCOOH] (2.1×10^{15} molecule cm^{-3}) and a large excess of H_2 (7.5×10^{16} molecule cm^{-3}). These traces were recorded at 373 K and 53 Torr total pressure (Ar).

S4. Summary of room temperature measurements and tabulation of data

Figure S4 shows a summary of the room temperature measurements recorded over several weeks and under various conditions. The mean value is $k_1 = (3.44 \pm 0.34) \times 10^{-13}$ cm^3 $molecule^{-1}$ s^{-1}, where the errors represent the 95% confidence interval.

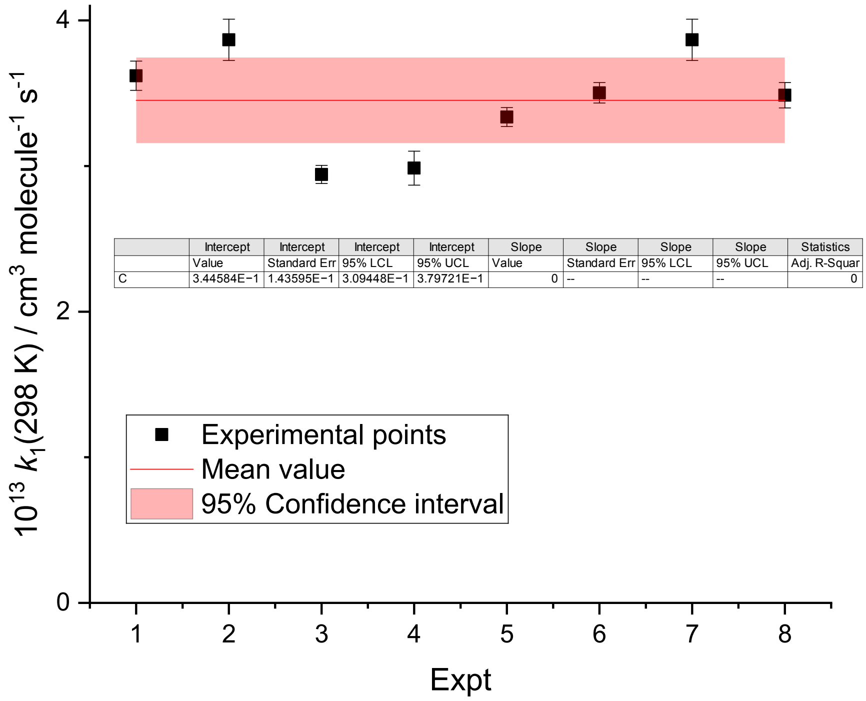

	Intercept	Intercept	Intercept	Intercept	Slope	Slope	Slope	Slope	Statistics
	Value	Standard Err	95% LCL	95% UCL	Value	Standard Err	95% LCL	95% UCL	Adj. R-Squar
C	3.44584E−1	1.43595E−1	3.09448E−1	3.79721E−1	0	--	--	--	0

Figure S4. Variation in k_1 (298 K) with experiment.

Table S1. Experimental k_1 over the T range 295 – 860 K determined via OH removal kinetics; these are the data shown in Figure 4 of the main manuscript.

T/K	[M]/Torr	Precursor / Photolysis	Rep/s^{-1}	F/R [a]	10^{13} k_1 / cm^3 $molecule^{-1}$ s^{-1}
300	~50	FA / 266 nm	5	F	3.86 ± 0.140
448	~50	FA / 266 nm	5	F	2.92 ± 0.09
498	~50	FA / 266 nm	5	F	3.46 ± 0.14
648	~50	FA / 266 nm	5	F	8.13 ± 0.29
698	~50	FA / 266 nm	5	F	9.71 ± 0.41
723	~50	FA /266 /nm	5	F	10.1 ± 0.6
T/K	[M]/Torr	Precursor / Photolysis	Rep/s^{-1}	F/R [a]	10^{13} k_1 / cm^3 $molecule^{-1}$ s^{-1}
298	52	FA / 213nm	3	F	3.50 ± 0.07
423	52	FA / 213nm	3	F	3.22 ± 1.22
521	52	FA / 213nm	3	F	4.18 ± 0.20
621	52	FA / 213nm	3	F	5.85 ± 0.35
694	53	FA / 213nm	3	F	7.14 ± 049
773	53	FA / 213nm	3	F	8.52 ± 0.96
860	53	FA / 213nm	3	F	12.9 ± 1.11
573	63	FA / 193 nm	5	F	5.59 ± 0.50
573	61	FA / 193 nm	10	F	6.62 ± 0.21
635	60	FA / 193 nm	10	F	12.6 ± 0.5
648	52	FA / 193 nm	10	F	12.8 ± 0.47
669	104	FA / 193 nm	10	F	9.92 ± 0.38
772	145	FA / 193 nm	10	F	11.0 ± 0.35
823	55	FA / 193 nm	10	F	13.8 ± 0.49
296	47	varied FA/266nm [b]	5	F	2.66± 0.06
296	36	varied FA/266nm	5	F	2.94 ± 0.06
498	58	varied FA/266nm	5	F	3.66 ± 0.15
648	11.6	varied FA/266nm	5	F	11.1 ± 0.81
648	55	varied FA/266nm	5	F	7.55 ± 0.38
698	11.7	varied FA/266nm	5	F	9.48 ± 0.90
698	26.8	varied FA/266nm	5	F	9.97 ± 1.09
698	55	varied FA/266nm	5	F	7.56 ± 0.27
773	58	varied FA/266nm	5	F	9.05 ± 0.37
857	60	varied FA/266nm	5	F	13.5 ± 0.68
298	~50	H_2O_2/266 nm	5	F	3.87 ± 0.14
403	~50	H_2O_2/266 nm	5	F	3.52 ± 0.26
523	~50	FA/213 nm	3	R	4.50 ± 0.40
673	~50	FA/213 nm	3	R	12.2 ± 0.40

723	~50	FA/213 nm	3	R	9.48± 0.42
723	58	FA/213 nm	3	R	10.8 ± 0.41
723	53	FA/213 nm	3	R	15.0 ± 0.50
773	11	ACAC/ 266 nm	10	F	11.4 ± 0.36
773	11	ACAC/ 266 nm	10	F	16.3 ± 0.54

(a) F/R refers to the direction of the gas flow, either forward (F) or reverse (R). (b) For the majority of the experiments the total flow was ~10 SCCM / Torr, i.e. 500 SCMM for 50 Torr, but for varied experiments the flow was significantly different.

S5. Formic acid photolysis

Figure S5 shows a schematic of HC(O)OH photolysis. The origin for the first excited singlet state at 448 kJ mol^{-1} (or 267.2 nm) has been determined experimentally by Ioannoni et al.[4] Experimentally, Brouard et al.[5] determined a barrier height of 475 kJ mol^{-1} (or 252 nm) as a threshold for OH production. Ioannoni et al. showed a clear decrease in the fluorescence lifetime of HC(O)OH(A) as the excitation wavelength decreased below 267 nm, suggesting that there are photolysis pathways above the A state origin, but below the barrier for OH production, suggesting H + HOCO as a possible channel for wavelengths 267.2 $\geq \lambda \geq$ 252 nm.

A number of photolysis wavelengths have been used in studies of the OH + FA reaction. Singleton and co-workers[6-8] used 222 nm photolysis of FA as their OH source. One can see from Fig S5 that absorption of a photon at this wavelength will produce excited FA well above the barrier for OH + HCO formation, consistent with the measured high quantum yield (~0.8) for OH production.[7]

Photolysis of FA at 248 nm produces excited state OH just above the barrier to dissociation to HCO + OH. Indeed OH is seen, but in our earlier studies of the H atom yield, we saw a significant prompt H photolysis and little discernible growth in H atom signal suggesting that $[OH]_0 << [H]_0$.

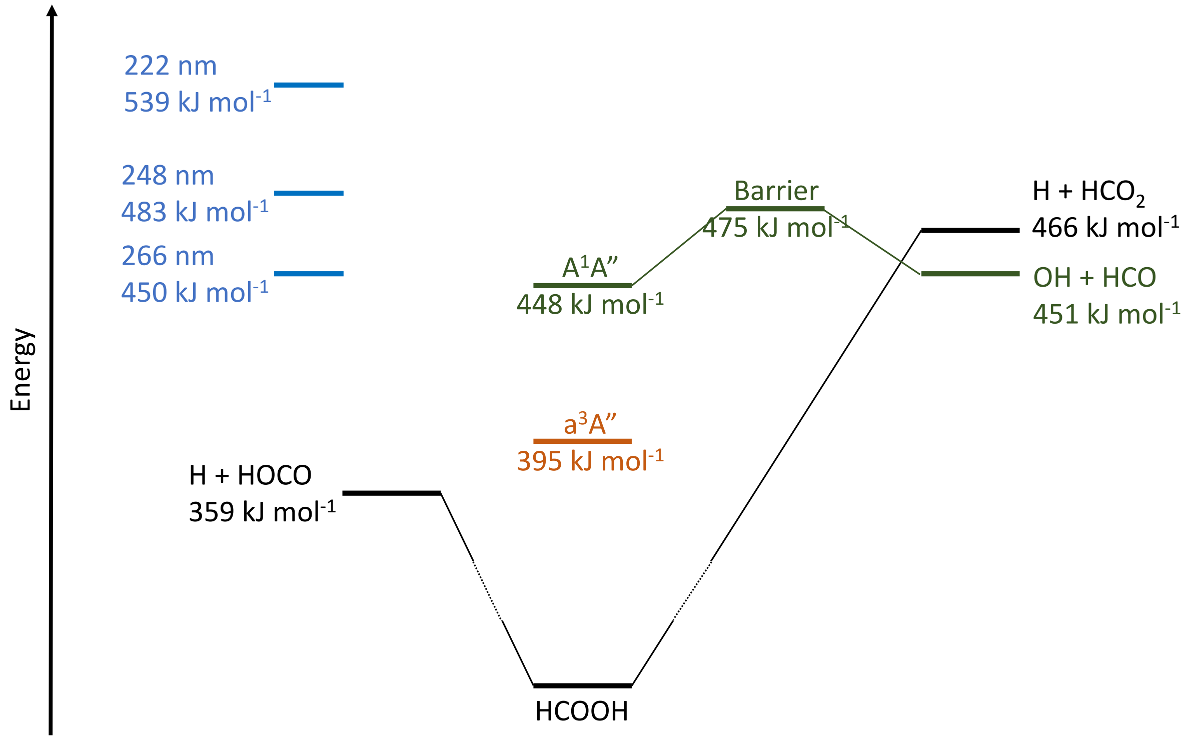

Figure S5. Schematic of HC(O)OH photolysis.

266 nm photolysis of FA produces even less OH and it may be that the photolytic OH that we observe is from residual H_2O_2 or products. Wine et al.[9] used 266 nm photolysis of O_3 followed by insertion of the result O(^{1}D) into H_2O as the OH source. As shown in Fig 9 of the main text, photolysis of formic acid does produce a significant initial H atom signal and the energetics limits the co-product to being HOCO. Our observation of photolytic products at wavelengths longer than 252 nm is consistent with the HC(O)OH fluorescence studies of Ioannoni et al. mentioned above.

S6. HOCO decomposition rate coefficients

The rate coefficients for reaction 3, HOCO decomposition, and the H atom yield are tabulated in Table S2.

$$HOCO \rightarrow OH + CO \qquad R3a$$

$$HOCO \rightarrow H + CO_2 \qquad R3b$$

Table S2. Experimental HOCO decomposition rate coefficients, k_3. All measurements were made via 266 nm photolysis of formic acid.

T / K	[M] / Torr	Bath gas	Measured k_3 / s^{-1}	MESMER k_3 / s^{-1}	H_{yield}
667	30.9	He	433 ± 52	793	
669	31.9	He	659 ± 83	837	
671	30.4	He	760 ± 110	844	
670	119.4	He	1390 ± 390	1764	
670	115.9	He	2080 ± 380	1736	
672	109.4	He	1870 ± 330	1748	
672	109.4	He	1580 ± 390	1748	
722	30.2	He	2020 ± 190	1940	
722	31.1	He	1610 ± 240	1970	
722	30.9	He	2280 ± 540	1970	
722	31.3	He	1900 ± 270	1980	
723	30.5	He	1370 ± 210	1980	
723	31.3	He	1510 ± 220	2010	
722	117.2	He	4340 ± 760	4350	
722	115.7	He	4770 ± 540	4310	
723	100.2	He	3920 ± 740	4030	
723	98.2	He	2620 ± 690	3980	
626	206.9	He	1800 ± 140	920	
626	206.9	He	1590 ± 130	920	
622	203.6	He	1500 ± 130	840	
622	203.6	He	1710 ± 160	840	
623	206.9	He	1100 ± 130	860	
623	206.9	He	1130 ± 120	860	
622	206	He			0.98 ± 0.02
667	201.1	He	3160 ± 310	2190	
666	201.1	He	3190 ± 320	2150	
666	202	He	2770 ± 270	2160	
666	202	He	2140 ± 260	2160	
668	202.7	He	2471 ± 260	2250	

T / K	[M] / Torr	Bath gas	Measured k_3 / s^{-1}	MESMER k_3 / s^{-1}	H_{yield}
668	202.7	He	2620 ± 250	2250	
667	202	He			0.96 ± 0.03
723	206.2	He	5010 ± 480	6120	
723	206.2	He	4570 ± 510	6120	
721	203.5	He	6870 ± 650	5880	
721	203.5	He	7160 ± 780	5880	
722	202.6	He	8530 ± 970	5960	
722	202.6	He	6860 ± 800	5960	
722	203	He			0.94 ± 0.03
767	206.3	He	11930 ± 800	11980	
766	206.3	He	12090 ± 680	11800	
768	204	He	11020 ± 760	12100	
768	204	He	12030 ± 650	12100	
770	203.3	He	13020 ± 940	12400	
770	203.3	He	12800± 1000	12400	
769	204	He			0.93 ± 0.02
673	30	Ar	1210 ± 340	720	
673	30	Ar	1240 ± 290	720	
673	30	Ar	1140 ± 420	720	
673	30	Ar	690 ± 240	720	
572	44.3	Ar	96 ± 22	97	
572	44.3	Ar	110 ± 230	97	
620	44.4	Ar	300 ± 44	310	
620	44.3	Ar	249 ± 27	310	
619	45	Ar	253 ± 27	310	
619	45	Ar	217 ± 27	310	
621	43.9	Ar	224 ± 28	320	
621	43.9	Ar	246 ± 31	320	
667	44.4	Ar	1560 ± 210	800	
668	44.4	Ar	1080 ± 180	820	

T / K	[M] / Torr	Bath gas	Measured k_3 / s^{-1}	MESMER k_3 / s^{-1}	H_{yield}
670	43.8	Ar	740 ± 120	840	
671	43.8	Ar	1100 ± 270	860	
671	45.2	Ar	1280 ± 240	880	
671	45.2	Ar	1290 ± 230	880	
717	44.8	Ar	2670 ± 490	1900	
717	44.8	Ar	2950 ± 450	1890	
720	44.4	Ar	3700 ± 540	1980	
720	44.4	Ar	2580 ± 600	1980	
720	44.2	Ar	3090 ± 660	1980	
761	44.8	Ar	4300 ± 760	3620	
765	44.8	Ar	4200 ±1300	3820	
767	44.4	Ar	4200 ± 1400	3900	
768	44.4	Ar	6000 ± 1500	3950	
770	44.4	Ar	5200 ± 1900	4060	
770	44.4	Ar	5400 ± 1600	4060	
673	40.1	Ar	1233 ± 97	847	
723	38.4	Ar	2178 ± 164	1902	
773	39.8	Ar	3109 ± 391	3935	

S7. Energy transfer parameters

Table S3 shows the energy transfer parameters for both WKB and Eckart tunnelling corrections.

Table S3. Fitted energy transfer parameters for HOCO in He and Ar bath gases

	WKB	Eckart
$\langle \Delta E \rangle d{,}298\ K$ Ar (cm^{-1})	51 ± 13	32 ± 9
n Ar	1.06 ± 0.31	1.09 ± 0.32
$\langle \Delta E \rangle d{,}298$ K He (cm^{-1})	74 ± 15	53 ± 11
n He	0.51 ± 0.24	0.55 ± 0.23
χ^2	377	421
$\langle \Delta E \rangle d{,}298\ K$ Ar (cm^{-1})	59 ± 15	36 ± 10
n Ar	0.87 ± 0.31	0.95 ± 0.33
$\langle \Delta E \rangle d{,}298$ K He (cm^{-1})	68 ± 15	52 ± 11
n He	0.60 ± 0.24	0.56 ± 0.33
$\langle \Delta E \rangle d{,}298\ K$ Ar (cm^{-1})	79 ± 2	51.2 ± 1.5
n Ar	0.5 fixed	0.5 fixed
$\langle \Delta E \rangle d{,}298$ K He (cm^{-1})	48 ± 1	35.9 ± 0.6
n He	1.0 fixed	1.0 fixed
χ^2	411	454
$\langle \Delta E \rangle d{,}298\ K$ Ar (cm^{-1})		61 ± 20
n Ar		0.94 ± 0.36
$\langle \Delta E \rangle d{,}298$ K He (cm^{-1})		77 ± 21
n He		0.55 ± 0.27
imFreqs (cm^{-1})		1793 ± 26
χ^2		381

S8. PLOG parameters

The rate coefficients from MESMER simulations over T range 400 – 1200 K, using WKB for the tunnelling and He energy transfer parameters, see Table S2, were used to determine PLOG parameters by fitting k_3 at a given pressure to the equation:

$$\ln(k) = \ln A \text{ x } n\ln(T) - (Ea/(1.987*T))$$

The resultant parameters are given in Table S4.

Table S4. PLOG parameters for HOCO decomposition to OH (k_{3a}) and H (k_{3b}).

	$\ln(k_{3a})$			$\ln(k_{3a})$		
p / atm	$\ln A_{3a}$-OH	n_{3a}-OH	Ea_{3a}-OH	$\ln A_{3b}$-H	n_{3b}-H	Ea_{3b}-H
0.001	20.50991	-1.63984	31496.92814	49.60555	-4.6854	19461.2939
0.01	38.828	-3.4365	29023.73945	51.85252	-4.64173	20956.68387
0.1	57.22245	-5.20496	28334.2854	53.55766	-4.53035	22455.58716
1	67.05926	-5.95933	28940.59967	55.79283	-4.54598	23885.12262
10	70.6569	-6.0418	29668.81818	55.11039	-4.24677	24623.47291
100	71.19572	-5.77597	30382.15009	51.1797	-3.51214	24913.6027
1000	67.70533	-5.00371	30785.61646	43.2513	-2.23294	24660.24946
10000	57.05871	-3.33601	30100.99639	29.23499	-0.17168	23324.4282
100000	41.7938	-1.15568	28313.46443	12.92099	2.1218	21266.39605

A cubic equation can adequately represent these PLOG parameters as a function of pressure:

$$\ln A_{3a}\text{-OH} = 66.316 + 3.400*\ln(p) + -0.4791*\ln(p)^2$$

$$n_{3a}\text{-OH} = -5.8979 + -0.2451*\ln(p) + 0.05554*\ln(p)^2 + 1.2468\text{E-}4*\ln(p)^3$$

$$Ea_{3a}\text{-OH} = 28758 + 276.11*\ln(p) + 48.982*\ln(p)^2 + -6.6848*\ln(p)^3$$

$$\ln A_{3b}\text{-H} = 55.530 + 0.1367*\ln(p) + -0.1954*\ln(p)^2 + -0.01239*\ln(p)^3$$

$$n_{3b}\text{-H} = -4.5296 + 0.09466*\ln(p) + 0.02298*\ln(p)^2 + 0.00171*\ln(p)^3$$

$$Ea_{3b}\text{-H} = 23768 + 470.76*\ln(p) + -36.835*\ln(p)^2 + -2.0147*\ln(p)^3$$

S9 Vibrational frequencies and rotational constants from ab initio calculations

Information on the structures, frequencies and rotational constants are available in the MESMER files. Below we have tabulated the frequencies and rotational constants.

Table S5. Vibrational frequencies and rotational constants

**HC(O)OH**	
Vibrational frequencies/cm^{-1}	616.807, 663.682, 1035.63, 1110.57, 1302.56, 1391.24, 1775.86, 3096.99, 3722.75
Rotational constants/cm^{-1}	0.340239 0.392778 2.5436
**OH -- HC(O)OH Pre-Reaction Complex**	
Vibrational frequencies/cm^{-1}	14.9523, 51.8151, 173.627, 267.586, 335.094, 466.553, 835.557, 1066.07, 1100.36, 1341.95, 1349.17, 1692.7, 3088.26, 3540.37, 3763.94
Rotational constants/cm^{-1}	0.127665 0.187332 0.395114
**OH + HC(O)OH TS1**	
Vibrational frequencies/cm^{-1}	260.366, 369.806, 455.94, 552.097, 637.215, 804.215, 1014.38, 1280.77, 1319.23, 1461.57, 1637.5, 1695.22, 3089.38, 3726.63
Rotational constants/cm^{-1}	0.159401 0.255829 0.407759
**OH + HC(O)OH TS2**	
Vibrational frequencies/cm^{-1}	112.522, 201.908, 239.334, 525.091, 631.404, 654.329, 819.984, 985.691, 1095.29, 1320.4, 1513.48, 1809, 3691.84, 3697.97
Rotational constants/cm^{-1}	0.108167 0.149047 0.394369
**HOCO**	
Vibrational frequencies/cm^{-1}	531.857, 612.712, 1075.03, 1253.42, 1882.48, 3804.67
Rotational constants/cm^{-1}	0.355234, 0.379699, 5.51315
**HOCO TS to OH + CO**	
Vibrational frequencies/cm^{-1}	206.024, 237.615, 653.281, 2130.28, 3729.88
Rotational constants/cm^{-1}	0.213232 0.226275 3.69925
**HOCO TS to H + CO_2**	
Vibrational frequencies/cm^{-1}	518.495 639.684 926.931 1283.24 2159.4
Rotational constants/cm^{-1}	0.348733 0.365484 7.60879

References

1. Glowacki, D. R.; Lockhart, J.; Blitz, M. A.; Klippenstein, S. J.; Pilling, M. J.; Robertson, S. H.; Seakins, P. W., Interception of Excited Vibrational Quantum States by O_2 in Atmospheric Association Reactions. *Science* **2012,** *337* (6098), 1066-1069.

2. Onel, L.; Blitz, M. A.; Seakins, P. W., A laser flash photolysis, laser induced fluorescence determination of the rate coefficient for the reaction of OH radicals with monoethanol amine (MEA) from 296 - 510 K. *J. Phys. Chem. Lett.* **2012,** *3*, 853-856.
3. Randi, P. A. S.; Pastega, D. F.; Bettega, M. H. F.; Jones, N. C.; Hoffmann, S. V.; Eden, S.; Barbosa, A. S.; Limao-Vieira, P., Electronically excited states of formic acid investigated by theoretical and experimental methods. *Spectrochimica Acta Part a-Molecular and Biomolecular Spectroscopy* **2023,** *289*, 122237.
4. Ioannoni, F.; Moule, D. C.; Clouthier, D. J., Laser spectroscopic and quantum chemical studies of the lowest excited-states of formic acid. *J. Phys. Chem.* **1990,** *94* (6), 2290-2294.
5. Brouard, M.; Simons, J. P.; Wang, J. X., State-to-state photodissociation dynamics in formic acid. *Faraday Discussions* **1991,** *91*, 63-72.
6. Jolly, G. S.; McKenney, D. J.; Singleton, D. L.; Paraskevopoulos, G.; Bossard, A. R., Rates of OH radical reactions .14. Rate-constant and mechanism for the reaction of hydroxyl radical with formic acid. *J. Phys. Chem.* **1986,** *90* (24), 6557-6562.
7. Jolly, G. S.; Singleton, D. L.; Paraskevopoulos, G., Direct determination of the quantum yield of OH in the laser photolysis of formic acid at 222 nm. *J. Phys. Chem.* **1987,** *91* (13), 3463-3465.
8. Singleton, D. L.; Paraskevopoulos, G.; Irwin, R. S.; Jolly, G. S.; McKenney, D. J., Rates of OH radical reactions .17. Rate and mechanism of the reaction of hydroxyl radicals with formic and deuteriated formic acids. *J. Am. Chem. Soc.* **1988,** *110* (23), 7786-7790.
9. Wine, P. H.; Astalos, R. J.; Mauldin, R. L., Kinetic and mechanistic study of the OH+HCOOH reaction. *J. Phys. Chem.* **1985,** *89* (12), 2620-2624.
