# Supplementary material for: Rate Coefficients and Branching Ratios for the Reaction of the OH Radical with Formic Acid under Low-Temperature Combustion Conditions and the Fate of the HOCO Product
Source: J Phys Chem A. 2025 Jul 2;129(28):6437–50. doi: 10.1021/acs.jpca.5c01814 (PMC12278225; doi:10.1021/acs.jpca.5c01814)
Supplement: Supplementary file 3 [file jp5c01814_si_003.pdf]

```
<?xml version="1.0" encoding="utf-8"?>
<?xml-stylesheet type='text/xsl' href='../../mesmer1.xsl'?>
<me:mesmer xmlns="http://www.xml-cml.org/schema"
xmlns:me="http://www.chem.leeds.ac.uk/mesmer"
xmlns:xsi="http://www.w3.org/2001/XMLSchema-instance">
<title> Glyoxal</title>
<moleculeList>
  <molecule id="N2">
    <atomArray>
      <atom id="a1" elementType="N" />
      <atom id="a2" elementType="N" />
    </atomArray>
    <bondArray>
      <bond atomRefs2="a2 a1" order="3" />
    </bondArray>
    <propertyList>
      <property dictRef="me:epsilon">
        <scalar>82</scalar>
      </property>
      <property dictRef="me:sigma">
        <scalar>3.74</scalar>
      </property>
      <property dictRef="me:MW">
        <scalar units="amu">28.0</scalar>
      </property>
    </propertyList>
  </molecule>
  <molecule spinMultiplicity="2" id="OH">
            <atomArray>
             <atom id="a1" elementType="O" x3="0.000000" y3="0.000000"
z3="0.107992"/>
             <atom id="a2" elementType="H" x3="0.000000" y3="0.000000" z3="-
0.863936"/>
            </atomArray>
            <bondArray>
             <bond atomRefs2="a2 a1" order="1"/>
            </bondArray>
      <propertyList>
        <property dictRef="me:ZPE">
          <scalar units="kJ/mol">0.0</scalar>
        </property>
        <property dictRef="me:rotConsts">
          <array units="cm-1">19.2438</array>
        </property>
        <property dictRef="me:symmetryNumber">
          <scalar>1</scalar>
        </property>
        <property dictRef="me:vibFreqs">
          <array units="cm-1">3722.0</array>
        </property>
```

<property dictRef="me:MW">
<scalar units="amu">17</scalar>
</property>
<property dictRef="me:spinMultiplicity">
<scalar>2</scalar>
</property>
<property dictRef="me:electronicExcitation">
<array units="cm-1">139.7</array>
</property>
<property dictRef="me:frequenciesScaleFactor" default="true">
<scalar>1</scalar>
</property>
</propertyList>
<me:DOSCMethod default="true" name="QMRotors" />
</molecule>
<molecule spinMultiplicity="2" id="FormicAcid">
<atomArray>
<atom id="a1" elementType="C" x3="-0.322384" y3="0.273379" z3="0.417795"/>
<atom id="a2" elementType="O" x3="0.221696" y3="1.157088" z3="-0.213192"/>
<atom id="a3" elementType="O" x3="-1.251652" y3="-0.586042" z3="-0.082974"/>
<atom id="a4" elementType="H" x3="-0.138334" y3="0.045272" z3="1.484393"/>
<atom id="a5" elementType="H" x3="-1.376187" y3="-0.343589" z3="-1.018654"/>
</atomArray>
<bondArray>
<bond atomRefs2="a5 a3" order="1"/>
<bond atomRefs2="a2 a1" order="2"/>
<bond atomRefs2="a3 a1" order="1" id="b1"/>
<bond atomRefs2="a1 a4" order="1"/>
</bondArray>
<propertyList>
<property dictRef="me:ZPE">
<scalar units="kJ/mol">0</scalar>
</property>
<property dictRef="me:vibFreqs">
<array units="cm-1"></array>
</property>
<property dictRef="me:spinMultiplicity">
<scalar units="cm-1">1</scalar>
</property>
<property dictRef="me:hessian">
<matrix matrixType="squareSymmetricLT" rows="15" units="Hartree/Bohr2">
0.4262198

0.27989 0.6064675

-0.0027796 -0.2486658 0.7792644

-0.2003625 -0.2086214 0.1397492 0.2088715

-0.2059015 -0.4107104 0.2574012 0.2742137
0.4594885
0.1331473 0.2498377 -0.3045323 -0.1602402 -
0.2860354 0.2756718
-0.1574739 -0.0925899 -0.0538504 -0.0222922 -
0.0577866 0.0028758 0.2200879

-0.0841557 -0.1453308 -0.0208141 -0.061622 -
0.0429519 -0.0015818 0.1576267 0.2502563

-0.0743335 -0.0443185 -0.1567996 0.0121547
0.0090703 0.0388773 0.125055 -0.0772539 0.5735689

-0.0641067 0.0139503 -0.040944 0.014032 -
0.0146606 0.0176455 -0.0030437 -0.0034006 -0.0284076 0.0532075

0.0162168 -0.0624049 0.0472416 -0.008037 -
0.0026422 0.0337774 -0.0120181 0.0053971 -0.0219389 0.0097608
0.0597533
-0.0464645 0.0409155 -0.290308 0.0015795
0.0153549 -0.0124129 -0.0074861 0.0020513 -0.0156686
0.0549646 -0.0573701 0.3173363
-0.0042767 0.007371 -0.0421752 -0.0002489 0.004135
0.0065715 -0.0372781 -0.0084485 -0.0344685 -0.000089 -
0.0059224 -0.0025936 0.0418926
-0.0060496 0.0119786 -0.0351629 0.0040666 -
0.0031841 0.0040021 0.0047679 -0.0673707 0.134441 -0.0056499 -
0.0001034 -0.0009515 0.002865 0.0586796
-0.0095697 0.0022311 -0.0276245 0.0067568 0.004209
0.0023961 -0.0665943 0.0975985 -0.439978 -0.0032586 -0.00171
0.0010531 0.0726658 -0.1023286 0.4641533
</matrix>
</property>
<property dictRef="me:sigma" default="true">
<scalar>5.0</scalar>
</property>
<property dictRef="me:epsilon" default="true">
<scalar>50.0</scalar>
</property>
<property dictRef="me:frequenciesScaleFactor" default="true">
<scalar>1</scalar>
</property>

<property dictRef="me:symmetryNumber" default="true">
<scalar>1</scalar>
</property>

```
    </propertyList>
    <me:DOSCMethod default="true" name="QMRotors" />
    <me:ExtraDOSCMethod xsi:type="me:HinderedRotorQM1D">
      <bondRef>b1</bondRef>
      <me:HinderedRotorPotential format="numerical" units="kJ/mol"
expansionSize="10" UseSineTerms="yes">
                  <me:PotentialPoint  angle="    0     "potential="  0      "/>
                  <me:PotentialPoint  angle="    30    "potential="  12.44487     "/>
                  <me:PotentialPoint  angle="    60    "potential="  38.8574      "/>
                  <me:PotentialPoint  angle="    90    "potential="  55.319285    "/>
                  <me:PotentialPoint  angle="    120   "potential="  47.285255    "/>
                  <me:PotentialPoint  angle="    150   "potential="  28.40791     "/>
                  <me:PotentialPoint  angle="    180   "potential="  19.559975    "/>
                  <me:PotentialPoint  angle="    210   "potential="  28.40791     "/>
                  <me:PotentialPoint  angle="    240   "potential="  47.285255    "/>
                  <me:PotentialPoint  angle="    270   "potential="  55.319285    "/>
                  <me:PotentialPoint  angle="    300   "potential="  38.8574      "/>
                  <me:PotentialPoint  angle="    330   "potential="  12.44487     "/>
                  <me:PotentialPoint  angle="    360   "potential="  0      "/>
      </me:HinderedRotorPotential>
          <me:CalculateInternalRotorInertia phaseDifference ="180.0"/>
    </me:ExtraDOSCMethod>
  </molecule>
  <molecule spinMultiplicity="2" id="Pre1">
         <atomArray>
          <atom id="a1" elementType="C" x3="1.092810" y3="0.175496"
z3="0.020738"/>
          <atom id="a2" elementType="O" x3="0.355551" y3="1.136637"
z3="0.001187"/>
          <atom id="a3" elementType="O" x3="0.706523" y3="-1.096977" z3="-
0.028274"/>
          <atom id="a4" elementType="H" x3="2.183878" y3="0.246463"
z3="0.080632"/>
          <atom id="a5" elementType="H" x3="-0.272242" y3="-1.101711" z3="-
0.079840"/>
          <atom id="a6" elementType="O" x3="-1.886176" y3="-0.147812"
z3="0.068940"/>
          <atom id="a7" elementType="H" x3="-1.875688" y3="0.667496" z3="-
0.460040"/>
         </atomArray>
         <bondArray>
          <bond atomRefs2="a7 a6" order="1"/>
          <bond atomRefs2="a5 a3" order="1"/>
          <bond atomRefs2="a3 a1" order="1"/>
          <bond atomRefs2="a2 a1" order="2"/>
          <bond atomRefs2="a1 a4" order="1"/>
          <bond atomRefs2="a5 a6" order="1"/>
         </bondArray>
    <propertyList>
      <property dictRef="me:ZPE">
```

<scalar units="kJ/mol">-22.84574138</scalar>
</property>
<property dictRef="me:vibFreqs">
<array units="cm-1"></array>
</property>
<property dictRef="me:spinMultiplicity">
<scalar units="cm-1">2</scalar>
</property>
<property dictRef="me:hessian">
<matrix matrixType="squareSymmetricLT" rows="21" units="Hartree/Bohr2">
0.63514756 0.10206277 0.23447383 0.04510834 -0.10133634 0.97740156 -
0.16081559 0.00148969 -0.16051799 0.17495275 -0.00041655 -0.07913543
0.04508925 0.00389904 0.03766745 -0.14145932 0.04957171 -0.65896285
0.22525464 -0.04540028 0.73063533 -0.29714226 -0.05208773 -0.04218471 -
0.03191326 -0.00517189 -0.07241505 0.51577471 -0.05413598 -0.07592192
0.00047244 -0.00430353 0.02892632 -0.01049748 0.12545890 0.08720530 -
0.02489535 0.00448542 -0.13771969 -0.07432692 -0.01008382 -0.02443379 -
0.06904104 -0.07846524 0.48154481 -0.14226409 -0.03535148 0.11393944
0.01602912 0.00065607 -0.01392798 -0.03303739 -0.01434041 0.02461310
0.16409166 -0.03690310 -0.08036158 0.04663281 -0.00286312 0.01927359
0.00523490 -0.00933155 0.00756675 0.00636115 0.05135378 0.04735158
0.12307486 0.04891254 -0.19474548 0.01058227 0.01131854 -0.04004271 -
0.00855807 -0.00178971 0.00864870 -0.12168543 -0.05836134 0.22477289 -
0.04243875 -0.01693190 0.03807336 0.00727822 0.00233318 0.00338614 -
0.14967110 -0.05097564 0.14226151 -0.00442861 -0.00234169 -0.00265199
0.19137752 -0.01220287 0.00065041 0.00644694 0.00373617 -0.00563628
0.00149519 -0.05651514 -0.04918124 0.07617208 -0.00219729 0.00608387
0.00025169 0.06647960 0.05102427 0.01014964 -0.00048985 0.02711509 -
0.00422366 -0.00111961 -0.01391783 0.17700230 0.08424789 -0.31414507 -
0.00418019 0.00015065 0.00031988 -0.17656786 -0.08129444 0.29501081
0.00684603 0.00055575 0.00404039 0.00008550 0.00012236 -0.00208535 -
0.00240098 -0.00059254 0.00080241 -0.00049930 0.00005751 -0.00068052 -
0.00332531 -0.00093895 -0.00022551 0.31584635 0.00208526 -0.00017155
0.00210907 -0.00236907 0.00105049 -0.00041386 -0.00290692 0.00213780
0.00370128 -0.00022328 -0.00041683 -0.00021030 0.00154634 -0.00471021 -
0.00427695 -0.17880551 0.10719325 -0.01217041 -0.00196317 -0.00925235
0.00230440 0.00068946 0.00588572 0.01356969 0.00608598 -0.01277459
0.00124222 -0.00012562 0.00065223 -0.00181222 -0.00443964 0.00462458
0.16542732 -0.09307392 0.09992261 0.00066709 0.00026291 0.00154117 -
0.00561674 -0.00142221 0.00124692 -0.00160972 -0.00111080 0.00058630
0.00010861 0.00002816 -0.00008111 0.00120802 0.00163849 -0.00195471 -
0.31655228 0.18067319 -0.16856100 0.32179502 -0.00048953 0.00046624
0.00058583 0.00041082 -0.00214614 0.00000982 0.00055434 -0.00073302 -
0.00217088 0.00010261 0.00050261 -0.00012142 -0.00010990 0.00176918
0.00278231 0.17960139 -0.10508296 0.09282691 -0.18006973 0.10522409
0.00019225 0.00081970 -0.00383629 0.00092726 -0.00049355 0.00083611
0.00162688 -0.00005388 -0.00112037 -0.00000115 0.00010744 0.00039449 -
0.00268894 0.00136818 0.00099254 -0.16727873 0.09216468 -0.08905820
0.16722243 -0.09391257 0.09179172
</matrix>

```
      </property>
      <property dictRef="me:sigma" default="true">
        <scalar>5.0</scalar>
      </property>
      <property dictRef="me:epsilon" default="true">
        <scalar>50.0</scalar>
      </property>
      <property dictRef="me:frequenciesScaleFactor" default="true">
        <scalar>1</scalar>
      </property>

      <property dictRef="me:symmetryNumber" default="true">
        <scalar>1</scalar>
      </property>
    </propertyList>
    <me:DOSCMethod default="true" name="QMRotors" />
  </molecule>

  <molecule spinMultiplicity="2" id="Pre2">
          <atomArray>
           <atom id="a1" elementType="C" x3="1.092810" y3="0.175496"
z3="0.020738"/>
           <atom id="a2" elementType="O" x3="0.355551" y3="1.136637"
z3="0.001187"/>
           <atom id="a3" elementType="O" x3="0.706523" y3="-1.096977" z3="-
0.028274"/>
           <atom id="a4" elementType="H" x3="2.183878" y3="0.246463"
z3="0.080632"/>
           <atom id="a5" elementType="H" x3="-0.272242" y3="-1.101711" z3="-
0.079840"/>
           <atom id="a6" elementType="O" x3="-1.886176" y3="-0.147812"
z3="0.068940"/>
           <atom id="a7" elementType="H" x3="-1.875688" y3="0.667496" z3="-
0.460040"/>
          </atomArray>
          <bondArray>
           <bond atomRefs2="a7 a6" order="1"/>
           <bond atomRefs2="a5 a3" order="1"/>
           <bond atomRefs2="a3 a1" order="1"/>
           <bond atomRefs2="a2 a1" order="2"/>
           <bond atomRefs2="a1 a4" order="1"/>
           <bond atomRefs2="a5 a6" order="1"/>
          </bondArray>
    <propertyList>
      <property dictRef="me:ZPE">
        <scalar units="kJ/mol">-8.057269679</scalar>
      </property>
      <property dictRef="me:vibFreqs">
        <array units="cm-1"></array>
      </property>
```

<property dictRef="me:spinMultiplicity">
<scalar units="cm-1">2</scalar>
</property>
<property dictRef="me:hessian">
<matrix matrixType="squareSymmetricLT" rows="21" units="Hartree/Bohr2">
0.63514756 0.10206277 0.23447383 0.04510834 -0.10133634 0.97740156 -
0.16081559 0.00148969 -0.16051799 0.17495275 -0.00041655 -0.07913543
0.04508925 0.00389904 0.03766745 -0.14145932 0.04957171 -0.65896285
0.22525464 -0.04540028 0.73063533 -0.29714226 -0.05208773 -0.04218471 -
0.03191326 -0.00517189 -0.07241505 0.51577471 -0.05413598 -0.07592192
0.00047244 -0.00430353 0.02892632 -0.01049748 0.12545890 0.08720530 -
0.02489535 0.00448542 -0.13771969 -0.07432692 -0.01008382 -0.02443379 -
0.06904104 -0.07846524 0.48154481 -0.14226409 -0.03535148 0.11393944
0.01602912 0.00065607 -0.01392798 -0.03303739 -0.01434041 0.02461310
0.16409166 -0.03690310 -0.08036158 0.04663281 -0.00286312 0.01927359
0.00523490 -0.00933155 0.00756675 0.00636115 0.05135378 0.04735158
0.12307486 0.04891254 -0.19474548 0.01058227 0.01131854 -0.04004271 -
0.00855807 -0.00178971 0.00864870 -0.12168543 -0.05836134 0.22477289 -
0.04243875 -0.01693190 0.03807336 0.00727822 0.00233318 0.00338614 -
0.14967110 -0.05097564 0.14226151 -0.00442861 -0.00234169 -0.00265199
0.19137752 -0.01220287 0.00065041 0.00644694 0.00373617 -0.00563628
0.00149519 -0.05651514 -0.04918124 0.07617208 -0.00219729 0.00608387
0.00025169 0.06647960 0.05102427 0.01014964 -0.00048985 0.02711509 -
0.00422366 -0.00111961 -0.01391783 0.17700230 0.08424789 -0.31414507 -
0.00418019 0.00015065 0.00031988 -0.17656786 -0.08129444 0.29501081
0.00684603 0.00055575 0.00404039 0.00008550 0.00012236 -0.00208535 -
0.00240098 -0.00059254 0.00080241 -0.00049930 0.00005751 -0.00068052 -
0.00332531 -0.00093895 -0.00022551 0.31584635 0.00208526 -0.00017155
0.00210907 -0.00236907 0.00105049 -0.00041386 -0.00290692 0.00213780
0.00370128 -0.00022328 -0.00041683 -0.00021030 0.00154634 -0.00471021 -
0.00427695 -0.17880551 0.10719325 -0.01217041 -0.00196317 -0.00925235
0.00230440 0.00068946 0.00588572 0.01356969 0.00608598 -0.01277459
0.00124222 -0.00012562 0.00065223 -0.00181222 -0.00443964 0.00462458
0.16542732 -0.09307392 0.09992261 0.00066709 0.00026291 0.00154117 -
0.00561674 -0.00142221 0.00124692 -0.00160972 -0.00111080 0.00058630
0.00010861 0.00002816 -0.00008111 0.00120802 0.00163849 -0.00195471 -
0.31655228 0.18067319 -0.16856100 0.32179502 -0.00048953 0.00046624
0.00058583 0.00041082 -0.00214614 0.00000982 0.00055434 -0.00073302 -
0.00217088 0.00010261 0.00050261 -0.00012142 -0.00010990 0.00176918
0.00278231 0.17960139 -0.10508296 0.09282691 -0.18006973 0.10522409
0.00019225 0.00081970 -0.00383629 0.00092726 -0.00049355 0.00083611
0.00162688 -0.00005388 -0.00112037 -0.00000115 0.00010744 0.00039449 -
0.00268894 0.00136818 0.00099254 -0.16727873 0.09216468 -0.08905820
0.16722243 -0.09391257 0.09179172
</matrix>
</property>
<property dictRef="me:sigma" default="true">
<scalar>5.0</scalar>
</property>
<property dictRef="me:epsilon" default="true">

```
      <scalar>50.0</scalar>
    </property>
    <property dictRef="me:frequenciesScaleFactor" default="true">
      <scalar>1</scalar>
    </property>

    <property dictRef="me:symmetryNumber" default="true">
      <scalar>1</scalar>
    </property>
  </propertyList>
  <me:DOSCMethod default="true" name="QMRotors" />
</molecule>
<molecule spinMultiplicity="2" id="TS1">
      <atomArray>
        <atom id="a1" elementType="C" x3="-0.98132" y3="-0.143307"
z3="0.010889" />
        <atom id="a2" elementType="O" x3="-0.18667" y3="-1.13481" z3="-
0.014726" />
        <atom id="a3" elementType="O" x3="-0.602629" y3="1.0799"
z3="0.041645" />
        <atom id="a4" elementType="H" x3="-2.066" y3="-0.359496"
z3="0.011018" />
        <atom id="a5" elementType="H" x3="0.584288" y3="0.849995"
z3="0.019831" />
        <atom id="a6" elementType="O" x3="1.56661" y3="0.125886" z3="-
0.106113" />
        <atom id="a7" elementType="H" x3="1.78598" y3="-0.261874"
z3="0.761677" />
      </atomArray>
      <bondArray>
        <bond atomRefs2="a6 a7" order="1" />
        <bond atomRefs2="a1 a2" order="2" />
        <bond atomRefs2="a1 a4" order="1" />
        <bond atomRefs2="a1 a3" order="1" id="b1" />
        <bond atomRefs2="a3 a5" order="1" />
            <bond atomRefs2="a3 a6" order="1" id="b2" />
      </bondArray>
  <propertyList>
    <property dictRef="me:ZPE">
      <scalar units="kJ/mol" >-0.32</scalar>
    </property>
    <property dictRef="me:vibFreqs">
      <array units="cm-1"></array>
    </property>
    <property dictRef="me:spinMultiplicity">
      <scalar units="cm-1">2</scalar>
    </property>
    <property dictRef="me:hessian">
      <matrix matrixType="squareSymmetricLT" rows="21" units="Hartree/Bohr2">
```

0.7668442

0.0105748 0.7397694

-0.0005792 0.0131127 0.1880211

-0.2618677 0.1697742 0.0035228 0.2748807

0.196055 -0.3034398 -0.005743 -0.2157695 0.435756

0.0045287 -0.0031882 -0.0669724 -0.003676
0.0095086 0.0312932

-0.1588011 -0.0679177 -0.001339 0.0452037
0.0051293 -0.000602 0.1963523

-0.1195947 -0.4051497 -0.0076535 0.0294575 -
0.0991302 -0.0028532 0.0663386 0.5692545

-0.0026119 -0.0064346 -0.0713954 0.002191 -
0.0026648 0.0307823 -0.000981 0.012671 0.0521729

-0.297524 -0.0561826 -0.0003371 -0.0258641
0.0069312 -0.0004847 -0.0070284 -0.0054975 -0.0004673
0.3264265

-0.0469902 -0.0481935 -0.0001014 0.0306908 -
0.0001887 -0.0016511 -0.0435232 -0.0156532 -0.0009387 0.053713
0.0690862
-0.0003783 -0.0003157 -0.05877 0.000366 -0.000706
0.0158877 -0.0011468 -0.001061 0.0139249 0.0004066
0.0016179 0.0235453

-0.0280778 -0.1277949 -0.0030734 -0.0334547
0.0377555 0.0024011 0.0453519 0.0335201 -0.0005888
0.0018985 0.0125955 0.0004506 -0.0261003

-0.0386688 0.0484236 0.0009412 0.0130638 -
0.0243527 -0.0035961 -0.0329061 -0.0585117 -0.0001129 -0.0006641 -
0.0089099 0.0005754 0.0696214 0.0679013

-0.0004098 0.0025469 0.0079841 -0.0002829 -
0.0022529 -0.0058212 -0.0027417 -0.0011179 -0.0383285 0.0004791 -
0.000024 0.0055159 0.0013976 -0.0010716 0.0688528

-0.017383 0.0694583 0.0027556 0.0031312 -
0.0286374 0.0019076 -0.1145727 -0.0031862 0.0003626 0.0012389 -
0.0071621 -0.000797 0.0348308 -0.0131562 0.0172588 0.1321723

-0.0018249 -0.0256971 -0.0007322 -0.0212085 -
0.0081588 0.0053212 0.0670602 0.0087277 0.0025316 0.0010655
0.0022444 -0.0016973 -0.0187182 -0.026028 -0.0149174 -
0.0731397 0.1367152
-0.0014348 -0.0106012 0.0014548 -0.0043634
0.0029762 -0.0042982 0.0052034 0.0036181 0.0146372 -
0.0006268 0.0010756 -0.0007756 -0.001603 0.0044698 -0.0418221
0.0839964 -0.1648366 0.4250678
-0.0031905 0.0020879 -0.0009497 -0.0020291 -
0.0014641 -0.0040746 -0.0065057 -0.0010378 0.0020953 0.0008526
0.0006761 0.001099 0.0055517 0.00271 -0.0157011 -
0.0394175 0.0467657 -0.0811719 0.0447385
0.0004489 -0.0057129 0.0001763 -0.0060083 -
0.0004859 -0.0035413 0.0058189 0.0004626 -0.0050517 0.0006346
0.0016146 0.0015866 -0.0069794 0.0014774 0.016837
0.0558232 -0.0878034 0.1632982 -0.0497379 0.0904476
0.0008854 0.0048801 -0.0003222 0.0022426 -0.001118
-0.0008713 0.001607 -0.0036035 -0.0017932 0.0010301
0.0000216 0.0006718 0.001016 -0.0012058 0.0036189 -0.105484
0.1743307 -0.394264 0.098703 -0.1733052 0.3929599
</matrix>
</property>
<property dictRef="me:sigma" default="true">
<scalar>5.0</scalar>
</property>
<property dictRef="me:epsilon" default="true">
<scalar>50.0</scalar>
</property>
<property dictRef="me:frequenciesScaleFactor" default="true">
<scalar>1</scalar>
</property>

<property dictRef="me:symmetryNumber" default="true">
<scalar>0.5</scalar>
</property>
</propertyList>
<me:DOSCMethod xsi:type="me:ClassicalCoupledRotors">
<me:MCPoints>500000</me:MCPoints>
<me:RotorArray>
<me:Rotor bondRef="b1" periodicity="1">
</me:Rotor>
<me:Rotor bondRef="b2" periodicity="1">
</me:Rotor>
</me:RotorArray>
<me:ExpansionSize>
<property dictRef="size">

<array >5 5</array>
</property>
</me:ExpansionSize>
<me:FourierSeries>
<property dictRef="coeficients">
<array units="kJ/mol"> 3985.5709 -545.1303 -1722.1683 -134.7869 -
70.5005 -188.1414 -613.996 -282.2113 -267.4196 -159.1857
10.6358 245.2404 218.9737 347.1025 365.0686 57.3571
-99.6071 -26.8602 -34.0298 -32.822 101.3592 -45.1851
113.7221 -3.0028 -13.9042</array>
</property>
</me:FourierSeries>
<me:FourierSeries>
<property dictRef="coeficients">
<array units="kJ/mol">0 73.5168 1.63 -3.0783 30.4075 0
-51.4142 29.9366 -15.2841 -118.2175 0 3.9356
32.511 50.8838 54.9548 0 -7.425 -20.5373 29.0099
69.1591 0 21.2285 1.6799 17.794 56.8859</array>
</property>
</me:FourierSeries>
<me:FourierSeries>
<property dictRef="coeficients">
<array units="kJ/mol">0 0 0 0 0 142.7535 -182.2907
-27.0838 38.7206 57.5148 -37.3965 125.9835 27.7951
-126.8799 -129.1822 -72.4943 131.8516 -47.6137 72.005
35.6564 66.3623 -109.8658 1.234 -64.3606 -
69.2083</array>
</property>
</me:FourierSeries>
<me:FourierSeries>
<property dictRef="coeficients">
<array units="kJ/mol">0 0 0 0 0 0 231.7059
231.0243 13.9756 15.562 0 17.4889 0.0216
33.0151 -33.4695 0 59.3966 59.1062 -25.4516 -
86.0002 0 83.4031 119.1956 136.3526 112.7835</array>
</property>
</me:FourierSeries>
</me:DOSCMethod>
</molecule>
<molecule spinMultiplicity="2" id="TS2">
<atomArray>
<atom id="a1" elementType="C" x3="0.457644" y3="-0.143150"
z3="0.000006"/>
<atom id="a2" elementType="O" x3="1.345893" y3="-0.958132"
z3="0.000010"/>
<atom id="a3" elementType="O" x3="0.582571" y3="1.208721"
z3="0.000042"/>
<atom id="a4" elementType="H" x3="-0.721428" y3="-0.412560" z3="-
0.000031"/>

<atom id="a5" elementType="H" x3="1.537677" y3="1.409716"
z3="0.000072"/>
<atom id="a6" elementType="O" x3="-2.068018" y3="-0.360218" z3="-
0.000064"/>
<atom id="a7" elementType="H" x3="-2.183659" y3="0.611532" z3="-
0.000044"/>
</atomArray>
<bondArray>
<bond atomRefs2="a6 a7" order="1"/>
<bond atomRefs2="a4 a1" order="1"/>
<bond atomRefs2="a1 a2" order="2"/>
<bond atomRefs2="a1 a3" order="1" id="b1"/>
<bond atomRefs2="a3 a5" order="1"/>
<bond atomRefs2="a6 a1" order="1" id="b2"/>
</bondArray>
<propertyList>
<property dictRef="me:ZPE">
<scalar units="kJ/mol" >5.26</scalar>
</property>
<property dictRef="me:vibFreqs">
<array units="cm-1"></array>
</property>
<property dictRef="me:spinMultiplicity">
<scalar units="cm-1">2</scalar>
</property>
<property dictRef="me:hessian">
<matrix matrixType="squareSymmetricLT" rows="21" units="Hartree/Bohr2">
0.7004963

-0.2493478 0.6936992

0.0002668 -0.0003049 0.1505257

-0.4700424 0.330991 0.000454 0.4814246

0.3285622 -0.3760176 0.0002682 -0.3880867
0.4448163

-0.0002127 0.0001355 -0.0577631 -0.0001127 -
0.0001867 0.0240662

-0.111077 -0.0517919 0.0002937 0.0203135
0.0472442 -0.0001502 0.5641085

-0.0057866 -0.2606871 0.0003215 0.0305431 -
0.0781663 -0.000194 0.0827322 0.4149408

-0.000022 0.0001149 -0.0511386 -0.0001157 -
0.0001725 0.0241461 -0.0001296 0.0000086 0.0349982

0.0110984 0.0016842 -0.0006339 -0.0447471
0.0079323 0.0004018 -0.0202921 -0.0145723 0.0002995 -
0.0507364
-0.025921 -0.0407913 -0.000086 0.0254001
0.0018779 0.0000834 -0.0270907 -0.0158599 -0.000082
0.0101159 0.0778352

0.0003007 0.0000745 -0.0510996 -0.0003123
0.0001874 0.0160618 0.0000137 0.0000354 0.0093334 -
0.0000752 0.0000225 0.0258591

-0.0105758 -0.0086092 -0.0004439 0.0002465 0.006083
0.0002291 -0.4601707 -0.0977922 0.0001641 0.0034601 -
0.0009535 -0.0000339 0.4678307

-0.0575867 -0.0184295 -0.0004367 0.0057421
0.0060866 0.0002091 -0.0488585 -0.0598052 0.0001575 -
0.0004283 -0.0047736 -0.0000754 0.101275 0.0768845

-0.0000678 -0.0000214 0.0059328 -0.0000083
0.0000419 -0.006049 0.0000261 -0.0000506 -0.0162213 -
0.0001134 0.0000292 0.0040913 -0.0000085 0.0000158 0.0119154

-0.1133128 -0.0271858 0.0002054 0.0102293 -
0.0000252 -0.0000217 0.0051778 0.0050017 -0.0002171 0.1014054
0.0032705 -0.0003704 -0.0010833 0.0000395 0.0001417
0.0269768
0.0022634 -0.0019166 0.0006643 -0.0017579
0.0015559 -0.0001891 -0.0009277 0.001801 -0.0002457
0.0051026 -0.0159724 -0.0002347 0.000095 0.0000177 -
0.0000148 -0.0453756 0.4956664
-0.0002401 0.00001 0.0038138 0.000077 -0.000183
-0.0011887 -0.0000599 -0.0000059 -0.0010535 -0.0000225 -
0.0001665 -0.0044311 0.0000868 0.0001898 0.0007873 0.0003064 -
0.0000693 0.0038088
-0.0065868 0.0042594 -0.0001421 0.0025755 -
0.0017098 -0.0001336 0.0019399 -0.0001258 0.0000207 -0.0001883
0.0151787 0.0004775 0.0002924 -0.0001831 0.0000301 -
0.0293932 0.0406002 -0.0001477 0.0313605
0.0078165 0.0041429 -0.0004264 -0.0028317 -
0.0001528 0.0001418 -0.0013076 -0.0022233 0.0002193 -0.0098345 -
0.0023158 -0.0000097 -0.0000981 0.0000195 -0.0000001 0.0642749 -
0.4811521 0.0002249 -0.0580196 0.4816816
-0.0000249 -0.0000086 -0.000271 0.000018
0.0000446 0.0007268 0.0000062 -0.0001149 -0.0000642
0.0001436 0.0001994 0.0001851 0.0000063 -0.0000601 -

```
0.0004565    -0.0000443   0.0000894    -0.0017366   -0.0001049   -0.0001498
        0.0016163
                 </matrix>
     </property>
     <property dictRef="me:sigma" default="true">
       <scalar>5.0</scalar>
     </property>
     <property dictRef="me:epsilon" default="true">
       <scalar>50.0</scalar>
     </property>
     <property dictRef="me:frequenciesScaleFactor" default="true">
       <scalar>1</scalar>
     </property>

     <property dictRef="me:symmetryNumber" default="true">
       <scalar>1</scalar>
     </property>
    </propertyList>
    <me:DOSCMethod xsi:type="me:ClassicalCoupledRotors">
     <me:MCPoints>500000</me:MCPoints>
     <me:RotorArray>
             <me:Rotor bondRef="b1" periodicity="1">
             </me:Rotor>
             <me:Rotor bondRef="b2" periodicity="1">
             </me:Rotor>
          </me:RotorArray>
          <me:ExpansionSize>
       <property dictRef="size">
         <array >6 5</array>
       </property>
          </me:ExpansionSize>
          <me:FourierSeries>
       <property dictRef="coeficients">
         <array units="kJ/mol">2364.5042  -200.942       -1894.9926   35.9313
         52.9416        5.3846         -175.1963      543.8824       -159.9105      84.1117
         46.789         42.2194        -8.2002        -98.6432       134.8169       -3.504
         80.1105        13.0548        5.9817         58.718         70.3189        16.1461
         70.0553        8.3763         -4.0272        40.342         80.8994        63.6902
         22.8793        44.1731</array>
       </property>
          </me:FourierSeries>
          <me:FourierSeries>
       <property dictRef="coeficients">
         <array units="kJ/mol">0      -0.1763        13.7713        2.7331         -18.0374
         0.0028         0      -0.1084        22.5687        4.7052         -29.0074
         0.0085         0      0.5305         9.5472         2.7247         -10.5098
         0.0029         0      1.3228         -6.543 0.2762         12.3578        -0.009 0
         1.9753         -19.556        -1.7065        30.8494        -0.0059</array>
       </property>
          </me:FourierSeries>
```

<me:FourierSeries>
<property dictRef="coeficients">
<array units="kJ/mol">0 0 0 0 0 0 -0.6787
7.5886 1.4472 -19.3067 -0.7696 23.4297 -1.0989
12.2817 2.3446 -31.2369 -1.249 37.9071 -1.1006
12.2841 2.3528 -31.2364 -1.2546 37.8984 -0.6812
7.5961 1.4634 -19.2977 -0.7802 23.4057</array>
</property>
</me:FourierSeries>
<me:FourierSeries>
<property dictRef="coeficients">
<array units="kJ/mol">0 0 0 0 0 0 0 -235.332
127.3308 -87.2159 20.4957 -0.0016 0 185.793 -
84.8573 9.2776 -32.7561 -0.0004 0 53.6099 -8.6458
-8.615 -25.3497 -0.0101 0 37.199 6.7929 -26.6281
-4.6401 -0.0029</array>
</property>
</me:FourierSeries>
</me:DOSCMethod>
</molecule>
<molecule spinMultiplicity="2" id="Post1">
<atomArray>
<atom id="a1" elementType="C" x3="1.092810" y3="0.175496"
z3="0.020738"/>
<atom id="a2" elementType="O" x3="0.355551" y3="1.136637"
z3="0.001187"/>
<atom id="a3" elementType="O" x3="0.706523" y3="-1.096977" z3="-
0.028274"/>
<atom id="a4" elementType="H" x3="2.183878" y3="0.246463"
z3="0.080632"/>
<atom id="a5" elementType="H" x3="-0.272242" y3="-1.101711" z3="-
0.079840"/>
<atom id="a6" elementType="O" x3="-1.886176" y3="-0.147812"
z3="0.068940"/>
<atom id="a7" elementType="H" x3="-1.875688" y3="0.667496" z3="-
0.460040"/>
</atomArray>
<bondArray>
<bond atomRefs2="a7 a6" order="1"/>
<bond atomRefs2="a5 a3" order="1"/>
<bond atomRefs2="a3 a1" order="1"/>
<bond atomRefs2="a2 a1" order="2"/>
<bond atomRefs2="a1 a4" order="1"/>
<bond atomRefs2="a5 a6" order="1"/>
</bondArray>
<propertyList>
<property dictRef="me:ZPE">
<scalar units="kJ/mol">-32.32014076</scalar>
</property>
<property dictRef="me:vibFreqs">

<array units="cm-1"></array>
</property>
<property dictRef="me:spinMultiplicity">
<scalar units="cm-1">2</scalar>
</property>
<property dictRef="me:hessian">
<matrix matrixType="squareSymmetricLT" rows="21" units="Hartree/Bohr2">
0.63514756 0.10206277 0.23447383 0.04510834 -0.10133634 0.97740156 -
0.16081559 0.00148969 -0.16051799 0.17495275 -0.00041655 -0.07913543
0.04508925 0.00389904 0.03766745 -0.14145932 0.04957171 -0.65896285
0.22525464 -0.04540028 0.73063533 -0.29714226 -0.05208773 -0.04218471 -
0.03191326 -0.00517189 -0.07241505 0.51577471 -0.05413598 -0.07592192
0.00047244 -0.00430353 0.02892632 -0.01049748 0.12545890 0.08720530 -
0.02489535 0.00448542 -0.13771969 -0.07432692 -0.01008382 -0.02443379 -
0.06904104 -0.07846524 0.48154481 -0.14226409 -0.03535148 0.11393944
0.01602912 0.00065607 -0.01392798 -0.03303739 -0.01434041 0.02461310
0.16409166 -0.03690310 -0.08036158 0.04663281 -0.00286312 0.01927359
0.00523490 -0.00933155 0.00756675 0.00636115 0.05135378 0.04735158
0.12307486 0.04891254 -0.19474548 0.01058227 0.01131854 -0.04004271 -
0.00855807 -0.00178971 0.00864870 -0.12168543 -0.05836134 0.22477289 -
0.04243875 -0.01693190 0.03807336 0.00727822 0.00233318 0.00338614 -
0.14967110 -0.05097564 0.14226151 -0.00442861 -0.00234169 -0.00265199
0.19137752 -0.01220287 0.00065041 0.00644694 0.00373617 -0.00563628
0.00149519 -0.05651514 -0.04918124 0.07617208 -0.00219729 0.00608387
0.00025169 0.06647960 0.05102427 0.01014964 -0.00048985 0.02711509 -
0.00422366 -0.00111961 -0.01391783 0.17700230 0.08424789 -0.31414507 -
0.00418019 0.00015065 0.00031988 -0.17656786 -0.08129444 0.29501081
0.00684603 0.00055575 0.00404039 0.00008550 0.00012236 -0.00208535 -
0.00240098 -0.00059254 0.00080241 -0.00049930 0.00005751 -0.00068052 -
0.00332531 -0.00093895 -0.00022551 0.31584635 0.00208526 -0.00017155
0.00210907 -0.00236907 0.00105049 -0.00041386 -0.00290692 0.00213780
0.00370128 -0.00022328 -0.00041683 -0.00021030 0.00154634 -0.00471021 -
0.00427695 -0.17880551 0.10719325 -0.01217041 -0.00196317 -0.00925235
0.00230440 0.00068946 0.00588572 0.01356969 0.00608598 -0.01277459
0.00124222 -0.00012562 0.00065223 -0.00181222 -0.00443964 0.00462458
0.16542732 -0.09307392 0.09992261 0.00066709 0.00026291 0.00154117 -
0.00561674 -0.00142221 0.00124692 -0.00160972 -0.00111080 0.00058630
0.00010861 0.00002816 -0.00008111 0.00120802 0.00163849 -0.00195471 -
0.31655228 0.18067319 -0.16856100 0.32179502 -0.00048953 0.00046624
0.00058583 0.00041082 -0.00214614 0.00000982 0.00055434 -0.00073302 -
0.00217088 0.00010261 0.00050261 -0.00012142 -0.00010990 0.00176918
0.00278231 0.17960139 -0.10508296 0.09282691 -0.18006973 0.10522409
0.00019225 0.00081970 -0.00383629 0.00092726 -0.00049355 0.00083611
0.00162688 -0.00005388 -0.00112037 -0.00000115 0.00010744 0.00039449 -
0.00268894 0.00136818 0.00099254 -0.16727873 0.09216468 -0.08905820
0.16722243 -0.09391257 0.09179172
</matrix>
</property>
<property dictRef="me:sigma" default="true">
<scalar>5.0</scalar>

</property>
<property dictRef="me:epsilon" default="true">
<scalar>50.0</scalar>
</property>
<property dictRef="me:frequenciesScaleFactor" default="true">
<scalar>1</scalar>
</property>

<property dictRef="me:symmetryNumber" default="true">
<scalar>1</scalar>
</property>
</propertyList>
<me:DOSCMethod default="true" name="QMRotors" />
</molecule>
<molecule spinMultiplicity="2" id="Post2">
<atomArray>
<atom id="a1" elementType="C" x3="1.092810" y3="0.175496"
z3="0.020738"/>
<atom id="a2" elementType="O" x3="0.355551" y3="1.136637"
z3="0.001187"/>
<atom id="a3" elementType="O" x3="0.706523" y3="-1.096977" z3="-
0.028274"/>
<atom id="a4" elementType="H" x3="2.183878" y3="0.246463"
z3="0.080632"/>
<atom id="a5" elementType="H" x3="-0.272242" y3="-1.101711" z3="-
0.079840"/>
<atom id="a6" elementType="O" x3="-1.886176" y3="-0.147812"
z3="0.068940"/>
<atom id="a7" elementType="H" x3="-1.875688" y3="0.667496" z3="-
0.460040"/>
</atomArray>
<bondArray>
<bond atomRefs2="a7 a6" order="1"/>
<bond atomRefs2="a5 a3" order="1"/>
<bond atomRefs2="a3 a1" order="1"/>
<bond atomRefs2="a2 a1" order="2"/>
<bond atomRefs2="a1 a4" order="1"/>
<bond atomRefs2="a5 a6" order="1"/>
</bondArray>
<propertyList>
<property dictRef="me:ZPE">
<scalar units="kJ/mol">-90</scalar>
</property>
<property dictRef="me:vibFreqs">
<array units="cm-1"></array>
</property>
<property dictRef="me:spinMultiplicity">
<scalar units="cm-1">2</scalar>
</property>
<property dictRef="me:hessian">

<matrix matrixType="squareSymmetricLT" rows="21" units="Hartree/Bohr2">
0.63514756 0.10206277 0.23447383 0.04510834 -0.10133634 0.97740156 -
0.16081559 0.00148969 -0.16051799 0.17495275 -0.00041655 -0.07913543
0.04508925 0.00389904 0.03766745 -0.14145932 0.04957171 -0.65896285
0.22525464 -0.04540028 0.73063533 -0.29714226 -0.05208773 -0.04218471 -
0.03191326 -0.00517189 -0.07241505 0.51577471 -0.05413598 -0.07592192
0.00047244 -0.00430353 0.02892632 -0.01049748 0.12545890 0.08720530 -
0.02489535 0.00448542 -0.13771969 -0.07432692 -0.01008382 -0.02443379 -
0.06904104 -0.07846524 0.48154481 -0.14226409 -0.03535148 0.11393944
0.01602912 0.00065607 -0.01392798 -0.03303739 -0.01434041 0.02461310
0.16409166 -0.03690310 -0.08036158 0.04663281 -0.00286312 0.01927359
0.00523490 -0.00933155 0.00756675 0.00636115 0.05135378 0.04735158
0.12307486 0.04891254 -0.19474548 0.01058227 0.01131854 -0.04004271 -
0.00855807 -0.00178971 0.00864870 -0.12168543 -0.05836134 0.22477289 -
0.04243875 -0.01693190 0.03807336 0.00727822 0.00233318 0.00338614 -
0.14967110 -0.05097564 0.14226151 -0.00442861 -0.00234169 -0.00265199
0.19137752 -0.01220287 0.00065041 0.00644694 0.00373617 -0.00563628
0.00149519 -0.05651514 -0.04918124 0.07617208 -0.00219729 0.00608387
0.00025169 0.06647960 0.05102427 0.01014964 -0.00048985 0.02711509 -
0.00422366 -0.00111961 -0.01391783 0.17700230 0.08424789 -0.31414507 -
0.00418019 0.00015065 0.00031988 -0.17656786 -0.08129444 0.29501081
0.00684603 0.00055575 0.00404039 0.00008550 0.00012236 -0.00208535 -
0.00240098 -0.00059254 0.00080241 -0.00049930 0.00005751 -0.00068052 -
0.00332531 -0.00093895 -0.00022551 0.31584635 0.00208526 -0.00017155
0.00210907 -0.00236907 0.00105049 -0.00041386 -0.00290692 0.00213780
0.00370128 -0.00022328 -0.00041683 -0.00021030 0.00154634 -0.00471021 -
0.00427695 -0.17880551 0.10719325 -0.01217041 -0.00196317 -0.00925235
0.00230440 0.00068946 0.00588572 0.01356969 0.00608598 -0.01277459
0.00124222 -0.00012562 0.00065223 -0.00181222 -0.00443964 0.00462458
0.16542732 -0.09307392 0.09992261 0.00066709 0.00026291 0.00154117 -
0.00561674 -0.00142221 0.00124692 -0.00160972 -0.00111080 0.00058630
0.00010861 0.00002816 -0.00008111 0.00120802 0.00163849 -0.00195471 -
0.31655228 0.18067319 -0.16856100 0.32179502 -0.00048953 0.00046624
0.00058583 0.00041082 -0.00214614 0.00000982 0.00055434 -0.00073302 -
0.00217088 0.00010261 0.00050261 -0.00012142 -0.00010990 0.00176918
0.00278231 0.17960139 -0.10508296 0.09282691 -0.18006973 0.10522409
0.00019225 0.00081970 -0.00383629 0.00092726 -0.00049355 0.00083611
0.00162688 -0.00005388 -0.00112037 -0.00000115 0.00010744 0.00039449 -
0.00268894 0.00136818 0.00099254 -0.16727873 0.09216468 -0.08905820
0.16722243 -0.09391257 0.09179172
</matrix>
</property>
<property dictRef="me:sigma" default="true">
<scalar>5.0</scalar>
</property>
<property dictRef="me:epsilon" default="true">
<scalar>50.0</scalar>
</property>
<property dictRef="me:frequenciesScaleFactor" default="true">
<scalar>1</scalar>

</property>

<property dictRef="me:symmetryNumber" default="true">
<scalar>1</scalar>
</property>
</propertyList>
<me:DOSCMethod default="true" name="QMRotors" />
</molecule>
<molecule spinMultiplicity="2" id="HOCO">
<atomArray>
<atom id="a1" elementType="C" x3="0.000000" y3="0.408982"
z3="0.000000"/>
<atom id="a2" elementType="O" x3="-1.170899" y3="0.254212"
z3="0.000000"/>
<atom id="a3" elementType="O" x3="0.944992" y3="-0.546685"
z3="0.000000"/>
<atom id="a4" elementType="H" x3="1.807260" y3="-0.114112"
z3="0.000000"/>
</atomArray>
<bondArray>
<bond atomRefs2="a1 a2" order="2"/>
<bond atomRefs2="a1 a3" order="1" id="b1"/>
<bond atomRefs2="a3 a4" order="1"/>
</bondArray>
<propertyList>
<property dictRef="me:ZPE">
<scalar units="kJ/mol">-81.6609265</scalar>
</property>
<property dictRef="me:vibFreqs">
<array units="cm-1"></array>
</property>
<property dictRef="me:spinMultiplicity">
<scalar units="cm-1">2</scalar>
</property>
<property dictRef="me:hessian">
<matrix matrixType="squareSymmetricLT" rows="12" units="Hartree/Bohr2">
0.82660911 -0.30227171 0.49418563 0. 0. 0.01457088 -0.56571664 0.37330495 0.
0.66880116 0.37475280 -0.36179056 0. -0.39436274 0.31681490 0. 0. -0.01120230
0. 0. 0.00860310 -0.23089482 -0.11070475 0. -0.09868595 0.02678745 0.
0.52442604 -0.05798377 -0.14197119 0. 0.02748986 0.04281186 0. -0.16745690
0.46122585 0. 0. 0.00808875 0. 0. -0.00620308 0. 0. 0.00451231 -0.02999766
0.03967151 0. -0.00439857 -0.00717752 0. -0.19484527 0.19795081 0. 0.22924150
-0.01449732 0.00957612 0. -0.00643207 0.00216381 0. 0.25137420 -0.36206652 0.
-0.23044480 0.35032659 0. 0. -0.01145733 0. 0. 0.00880228 0. 0. -0.00639798 0. 0.
0.00905303
</matrix>
</property>
<property dictRef="me:sigma" default="true">
<scalar>5.0</scalar>
</property>

```
      <property dictRef="me:epsilon" default="true">
        <scalar>50.0</scalar>
      </property>
      <property dictRef="me:frequenciesScaleFactor" default="true">
        <scalar>0.9522</scalar>
      </property>
      <property dictRef="me:symmetryNumber" default="true">
        <scalar>1</scalar>
      </property>
    </propertyList>
    <me:DOSCMethod default="true" name="QMRotors" />
    <me:ExtraDOSCMethod xsi:type="me:HinderedRotorQM1D">
      <bondRef>b1</bondRef>
      <me:HinderedRotorPotential format="numerical" units="kJ/mol"
expansionSize="10" UseSineTerms="yes">
                <me:PotentialPoint angle=" 0 "potential=" 0 "/>
                <me:PotentialPoint angle=" 10 "potential=" 1.076455 "/>
                <me:PotentialPoint angle=" 20 "potential=" 4.227055 "/>
                <me:PotentialPoint angle=" 30 "potential=" 9.110485 "/>
                <me:PotentialPoint angle=" 40 "potential=" 15.28041 "/>
                <me:PotentialPoint angle=" 50 "potential=" 22.10671 "/>
                <me:PotentialPoint angle=" 60 "potential=" 28.82799 "/>
                <me:PotentialPoint angle=" 70 "potential=" 34.6566 "/>
                <me:PotentialPoint angle=" 80 "potential=" 38.8574 "/>
                <me:PotentialPoint angle=" 90 "potential=" 40.9578 "/>
                <me:PotentialPoint angle=" 100 "potential=" 40.64274 "/>
                <me:PotentialPoint angle=" 110 "potential=" 38.06975 "/>
                <me:PotentialPoint angle=" 120 "potential=" 33.65891 "/>
                <me:PotentialPoint angle=" 130 "potential=" 28.14536 "/>
                <me:PotentialPoint angle=" 140 "potential=" 22.290495 "/>
                <me:PotentialPoint angle=" 150 "potential=" 16.85571 "/>
                <me:PotentialPoint angle=" 160 "potential=" 12.49738 "/>
                <me:PotentialPoint angle=" 170 "potential=" 9.66184 "/>
                <me:PotentialPoint angle=" 180 "potential=" 8.690405 "/>
                <me:PotentialPoint angle=" 190 "potential=" 9.66184 "/>
                <me:PotentialPoint angle=" 200 "potential=" 12.49738 "/>
                <me:PotentialPoint angle=" 210 "potential=" 16.85571 "/>
                <me:PotentialPoint angle=" 220 "potential=" 22.290495 "/>
                <me:PotentialPoint angle=" 230 "potential=" 28.14536 "/>
                <me:PotentialPoint angle=" 240 "potential=" 33.65891 "/>
                <me:PotentialPoint angle=" 250 "potential=" 38.06975 "/>
                <me:PotentialPoint angle=" 260 "potential=" 40.64274 "/>
                <me:PotentialPoint angle=" 270 "potential=" 40.9578 "/>
                <me:PotentialPoint angle=" 280 "potential=" 38.8574 "/>
                <me:PotentialPoint angle=" 290 "potential=" 34.6566 "/>
                <me:PotentialPoint angle=" 300 "potential=" 28.82799 "/>
                <me:PotentialPoint angle=" 310 "potential=" 22.10671 "/>
                <me:PotentialPoint angle=" 320 "potential=" 15.28041 "/>
                <me:PotentialPoint angle=" 330 "potential=" 9.110485 "/>
                <me:PotentialPoint angle=" 340 "potential=" 4.227055 "/>
```

```
                    <me:PotentialPoint  angle=" 350  "potential="  1.076455      "/>
                    <me:PotentialPoint  angle=" 360  "potential="  0        "/>
     </me:HinderedRotorPotential>
   </me:ExtraDOSCMethod>
  </molecule>
  <molecule spinMultiplicity="2" id="water">
            <atomArray>
             <atom id="a1" elementType="H" x3="0.759508" y3="-0.469893"
z3="0.000000"/>
             <atom id="a2" elementType="O" x3="0.000000" y3="0.117473"
z3="0.000000"/>
             <atom id="a3" elementType="H" x3="-0.759508" y3="-0.469893"
z3="0.000000"/>
            </atomArray>
            <bondArray>
             <bond atomRefs2="a1 a2" order="1"/>
             <bond atomRefs2="a2 a3" order="1"/>
            </bondArray>
    <propertyList>
      <property dictRef="me:ZPE">
        <scalar units="kJ/mol">0</scalar>
      </property>
      <property dictRef="me:vibFreqs">
        <array units="cm-1"></array>
      </property>
      <property dictRef="me:spinMultiplicity">
        <scalar units="cm-1">1</scalar>
      </property>
      <property dictRef="me:hessian">
        <matrix matrixType="squareSymmetricLT" rows="9" units="Hartree/Bohr2">
0.10503663 -0.21273897 0.43143138 -0.03130607 0.06982192 0.05843716 -
0.10005101 0.20551299 0.05203861 0.13647704 0.20197176 -0.41553118 -
0.11137358 -0.26201210 0.51083023 0.02504278 -0.05778144 -0.06146036
0.06901347 -0.07051948 0.50677778 -0.00498562 0.00722599 -0.02073254 -
0.03642602 0.06004033 -0.09405625 0.04141164 0.01076721 -0.01590020
0.04155165 0.05649911 -0.09529905 0.12830093 -0.06726632 0.11119925
0.00626328 -0.01204048 0.00302320 -0.12105208 0.18189306 -0.44531742
0.11478879 -0.16985258 0.44229422
                    </matrix>
      </property>
      <property dictRef="me:sigma" default="true">
        <scalar>5.0</scalar>
      </property>
      <property dictRef="me:epsilon" default="true">
        <scalar>50.0</scalar>
      </property>
      <property dictRef="me:frequenciesScaleFactor" default="true">
        <scalar>1</scalar>
      </property>
```

<property dictRef="me:symmetryNumber" default="true">
<scalar>1</scalar>
</property>
</propertyList>
<me:DOSCMethod default="true" name="QMRotors" />
</molecule>
<molecule spinMultiplicity="2" id="HOCO_OH_TS">
<atomArray>
<atom id="a1" elementType="C" x3="-0.766406" y3="-0.083114" z3="-
0.356823"/>
<atom id="a2" elementType="O" x3="-1.603898" y3="-0.579884"
z3="0.222677"/>
<atom id="a3" elementType="O" x3="1.191373" y3="0.312290"
z3="0.442063"/>
<atom id="a4" elementType="H" x3="1.536981" y3="0.720419" z3="-
0.368238"/>
</atomArray>
<bondArray>
<bond atomRefs2="a1 a2" order="2"/>
<bond atomRefs2="a3 a4" order="1"/>
<bond atomRefs2="a3 a1" order="1" id="b1"/>
</bondArray>
<propertyList>
<property dictRef="me:ZPE">
<scalar units="kJ/mol">25.5560735</scalar>
</property>
<property dictRef="me:vibFreqs">
<array units="cm-1"></array>
</property>
<property dictRef="me:spinMultiplicity">
<scalar units="cm-1">2</scalar>
</property>
<property dictRef="me:hessian">
<matrix matrixType="squareSymmetricLT" rows="12" units="Hartree/Bohr2">
0.64111025 0.38311313 0.23287652 -0.45368143 -0.27483783 0.33219897 -
0.65546051 -0.38719857 0.45075185 0.66247553 -0.38628160 -0.23226796
0.27122152 0.38988957 0.23258837 0.44817346 0.27032660 -0.32446733 -
0.45113111 -0.26958410 0.31914344 0.01811574 0.00758629 -0.00485720 -
0.00719725 -0.00423808 0.00582558 0.06043106 0.00314318 0.00154659
0.00219273 -0.00277612 -0.00187725 -0.00011844 0.07417948 0.09141401
0.00763621 0.00652903 -0.00967065 0.00171471 -0.00154527 0.00506274 -
0.15028468 -0.18268469 0.36770902 -0.00376548 -0.00350085 0.00778677
0.00018224 0.00063011 -0.00286793 -0.07134955 -0.07454654 0.14093376
0.07493279 0.00002529 -0.00215515 0.00142358 0.00008512 0.00155684 -
0.00062406 -0.07752769 -0.09108335 0.17770093 0.07741727 0.09168166 -
0.00212825 -0.00201780 0.00193900 -0.00133545 -0.00009215 0.00026115
0.14931630 0.18061040 -0.36310111 -0.14585261 -0.17850045 0.36090096
</matrix>
</property>
<property dictRef="me:sigma" default="true">

```
        <scalar>5.0</scalar>
      </property>
      <property dictRef="me:epsilon" default="true">
        <scalar>50.0</scalar>
      </property>
      <property dictRef="me:frequenciesScaleFactor" default="true">
        <scalar>1</scalar>
      </property>

      <property dictRef="me:symmetryNumber" default="true">
        <scalar>1</scalar>
      </property>
    </propertyList>
    <me:DOSCMethod default="true" name="QMRotors" />
    <me:ExtraDOSCMethod xsi:type="me:HinderedRotorQM1D">
      <bondRef>b1</bondRef>
      <me:HinderedRotorPotential format="numerical" units="kJ/mol"
expansionSize="10" UseSineTerms="yes">
                  <me:PotentialPoint  angle=" 0    "potential="  0        "/>
                  <me:PotentialPoint  angle=" 10   "potential="  0.183785    "/>
                  <me:PotentialPoint  angle=" 20   "potential="  0.68263     "/>
                  <me:PotentialPoint  angle=" 30   "potential="  1.52279     "/>
                  <me:PotentialPoint  angle=" 40   "potential="  2.651755    "/>
                  <me:PotentialPoint  angle=" 50   "potential="  4.04327     "/>
                  <me:PotentialPoint  angle=" 60   "potential="  5.644825    "/>
                  <me:PotentialPoint  angle=" 70   "potential="  7.40391     "/>
                  <me:PotentialPoint  angle=" 80   "potential="  9.18925     "/>
                  <me:PotentialPoint  angle=" 90   "potential="  10.92208    "/>
                  <me:PotentialPoint  angle=" 100 "potential="  12.54989    "/>
                  <me:PotentialPoint  angle=" 110 "potential="  14.02017    "/>
                  <me:PotentialPoint  angle=" 120 "potential="  15.254155   "/>
                  <me:PotentialPoint  angle=" 130 "potential="  16.251845   "/>
                  <me:PotentialPoint  angle=" 140 "potential="  17.039495   "/>
                  <me:PotentialPoint  angle=" 150 "potential="  17.64336    "/>
                  <me:PotentialPoint  angle=" 160 "potential="  18.06344    "/>
                  <me:PotentialPoint  angle=" 170 "potential="  18.299735   "/>
                  <me:PotentialPoint  angle=" 180 "potential="  18.3785     "/>
                  <me:PotentialPoint  angle=" 190 "potential="  18.299735   "/>
                  <me:PotentialPoint  angle=" 200 "potential="  18.06344    "/>
                  <me:PotentialPoint  angle=" 210 "potential="  17.64336    "/>
                  <me:PotentialPoint  angle=" 220 "potential="  17.039495   "/>
                  <me:PotentialPoint  angle=" 230 "potential="  16.251845   "/>
                  <me:PotentialPoint  angle=" 240 "potential="  15.254155   "/>
                  <me:PotentialPoint  angle=" 250 "potential="  14.02017    "/>
                  <me:PotentialPoint  angle=" 260 "potential="  12.54989    "/>
                  <me:PotentialPoint  angle=" 270 "potential="  10.92208    "/>
                  <me:PotentialPoint  angle=" 280 "potential="  9.18925     "/>
                  <me:PotentialPoint  angle=" 290 "potential="  7.40391     "/>
                  <me:PotentialPoint  angle=" 300 "potential="  5.644825    "/>
                  <me:PotentialPoint  angle=" 310 "potential="  4.04327     "/>
```

<me:PotentialPoint angle=" 320 "potential=" 2.651755 "/>
<me:PotentialPoint angle=" 330 "potential=" 1.52279 "/>
<me:PotentialPoint angle=" 340 "potential=" 0.68263 "/>
<me:PotentialPoint angle=" 350 "potential=" 0.183785 "/>
<me:PotentialPoint angle=" 360 "potential=" 0 "/>
</me:HinderedRotorPotential>
<me:CalculateInternalRotorInertia phaseDifference ="180.0"/>
</me:ExtraDOSCMethod>
</molecule>
<molecule spinMultiplicity="2" id="HOCO_H_TS">
<atomArray>
<atom id="a1" elementType="C" x3="-0.358592" y3="-2.026237" z3="-0.104704"/>
<atom id="a2" elementType="O" x3="-1.138954" y3="-1.981970" z3="-0.974920"/>
<atom id="a3" elementType="O" x3="0.721902" y3="-1.936766" z3="0.434781"/>
<atom id="a4" elementType="H" x3="1.749054" y3="-1.565997" z3="-0.313406"/>
</atomArray>
<bondArray>
<bond atomRefs2="a1 a2" order="1"/>
<bond atomRefs2="a1 a3" order="1" id="b1"/>
<bond atomRefs2="a3 a4" order="1"/>
</bondArray>
<propertyList>
<property dictRef="me:ZPE">
<scalar units="kJ/mol">29.5688735</scalar>
</property>
<property dictRef="me:vibFreqs">
<array units="cm-1"></array>
</property>
<property dictRef="me:spinMultiplicity">
<scalar units="cm-1">2</scalar>
</property>
<property dictRef="me:hessian">
<matrix matrixType="squareSymmetricLT" rows="12" units="Hartree/Bohr2">
0.87279189 -0.00946403 0.09697223 0.71169600 -0.00317237 0.72756420 -
0.42311607 0.01954997 -0.41150046 0.47347466 0.03782563 -0.04536651
0.02529194 -0.02027179 0.02275772 -0.49216279 0.00948767 -0.51453124
0.47941782 -0.01843802 0.51851819 -0.29983308 0.03536056 -0.35568142 -
0.07450445 -0.03571634 0.06458844 0.20841650 0.02455795 -0.05496599 -
0.04073165 -0.00785161 0.02491855 0.02930760 -0.06141464 0.02836405 -
0.30800215 -0.03138976 -0.19681444 -0.05839665 0.00521094 -0.04040180
0.40933658 0.03584733 0.24428437 -0.14984274 -0.04544650 0.05548588
0.02414586 0.01816250 -0.05184347 0.16592103 0.04470830 -0.04293779 -
0.04022415 -0.05291955 0.00336026 0.01861207 0.00857344 -0.00230976 -
0.02035725 0.06177042 0.00168338 -0.00966850 -0.01742431 -0.00273388
0.08846893 0.02507446 -0.01621852 -0.00952071 -0.01206486 0.03641485 -
0.11824361 -0.02442328 -0.00706813 0.03929538 0.01141368 -0.01312819

```
            </matrix>
      </property>
      <property dictRef="me:sigma" default="true">
        <scalar>5.0</scalar>
      </property>
      <property dictRef="me:epsilon" default="true">
        <scalar>50.0</scalar>
      </property>
      <property dictRef="me:frequenciesScaleFactor" default="true">
        <scalar>1</scalar>
      </property>
      <property dictRef="me:symmetryNumber" default="true">
        <scalar>1</scalar>
      </property>
    </propertyList>
    <me:DOSCMethod default="true" name="QMRotors" />
    <!--><me:ExtraDOSCMethod xsi:type="me:HinderedRotorQM1D">
      <bondRef>b1</bondRef>
      <me:HinderedRotorPotential format="numerical" units="kJ/mol"
expansionSize="10" UseSineTerms="yes">
            <me:PotentialPoint  angle=" 0    "potential="  0        "/>
            <me:PotentialPoint  angle=" 10   "potential="  0.183785    "/>
            <me:PotentialPoint  angle=" 20   "potential="  0.68263     "/>
            <me:PotentialPoint  angle=" 30   "potential="  1.52279     "/>
            <me:PotentialPoint  angle=" 40   "potential="  2.651755    "/>
            <me:PotentialPoint  angle=" 50   "potential="  4.04327     "/>
            <me:PotentialPoint  angle=" 60   "potential="  5.644825    "/>
            <me:PotentialPoint  angle=" 70   "potential="  7.40391     "/>
            <me:PotentialPoint  angle=" 80   "potential="  9.18925     "/>
            <me:PotentialPoint  angle=" 90   "potential="  10.92208    "/>
            <me:PotentialPoint  angle=" 100 "potential="  12.54989    "/>
            <me:PotentialPoint  angle=" 110 "potential="  14.02017    "/>
            <me:PotentialPoint  angle=" 120 "potential="  15.254155   "/>
            <me:PotentialPoint  angle=" 130 "potential="  16.251845   "/>
            <me:PotentialPoint  angle=" 140 "potential="  17.039495   "/>
            <me:PotentialPoint  angle=" 150 "potential="  17.64336    "/>
            <me:PotentialPoint  angle=" 160 "potential="  18.06344    "/>
            <me:PotentialPoint  angle=" 170 "potential="  18.299735   "/>
            <me:PotentialPoint  angle=" 180 "potential="  18.3785     "/>
            <me:PotentialPoint  angle=" 190 "potential="  18.299735   "/>
            <me:PotentialPoint  angle=" 200 "potential="  18.06344    "/>
            <me:PotentialPoint  angle=" 210 "potential="  17.64336    "/>
            <me:PotentialPoint  angle=" 220 "potential="  17.039495   "/>
            <me:PotentialPoint  angle=" 230 "potential="  16.251845   "/>
            <me:PotentialPoint  angle=" 240 "potential="  15.254155   "/>
            <me:PotentialPoint  angle=" 250 "potential="  14.02017    "/>
            <me:PotentialPoint  angle=" 260 "potential="  12.54989    "/>
            <me:PotentialPoint  angle=" 270 "potential="  10.92208    "/>
            <me:PotentialPoint  angle=" 280 "potential="  9.18925     "/>
            <me:PotentialPoint  angle=" 290 "potential="  7.40391     "/>
```

```
            <me:PotentialPoint  angle=" 300 "potential="  5.644825      "/>
            <me:PotentialPoint  angle=" 310 "potential="  4.04327       "/>
            <me:PotentialPoint  angle=" 320 "potential="  2.651755      "/>
            <me:PotentialPoint  angle=" 330 "potential="  1.52279       "/>
            <me:PotentialPoint  angle=" 340 "potential="  0.68263       "/>
            <me:PotentialPoint  angle=" 350 "potential="  0.183785      "/>
            <me:PotentialPoint  angle=" 360 "potential="  0       "/>
    </me:HinderedRotorPotential>
        <me:CalculateInternalRotorInertia phaseDifference ="0.0"/>
   </me:ExtraDOSCMethod>-->
  </molecule>
  <molecule spinMultiplicity="2" id="sink_OH">
   <propertyList>
    <property dictRef="me:ZPE">
     <scalar units="kJ/mol">-30</scalar>
    </property>
   </propertyList>
  </molecule>
  <molecule spinMultiplicity="2" id="sink_H">
   <propertyList>
    <property dictRef="me:ZPE">
     <scalar units="kJ/mol">-70</scalar>
    </property>
   </propertyList>
  </molecule>
  <molecule spinMultiplicity="2" id="TSadd">
        <atomArray>
            <atom id="a1" elementType="C" x3="-0.251177" y3="0.182110"
z3="0.402837"/>
            <atom id="a2" elementType="O" x3="0.192911" y3="1.201359" z3="-
0.218438"/>
            <atom id="a3" elementType="O" x3="-1.288075" y3="-0.552454"
z3="-0.070515"/>
            <atom id="a4" elementType="H" x3="-0.160810" y3="0.064022"
z3="1.493334"/>
            <atom id="a5" elementType="H" x3="-1.359710" y3="-0.348929"
z3="-1.019851"/>
            <atom id="a6" elementType="O" x3="1.225842" y3="-0.720475" z3="-
0.151514"/>
            <atom id="a7" elementType="H" x3="1.970708" y3="-0.141403"
z3="0.106147"/>
        </atomArray>
        <bondArray>
         <bond atomRefs2="a5 a3" order="1"/>
         <bond atomRefs2="a2 a1" order="1"/>
         <bond atomRefs2="a6 a7" order="1"/>
         <bond atomRefs2="a6 a1" order="1" id="b2"/>
         <bond atomRefs2="a3 a1" order="1" id="b1"/>
         <bond atomRefs2="a1 a4" order="1"/>
        </bondArray>
```

<propertyList>
<property dictRef="me:ZPE">
<scalar units="kJ/mol" >37.44</scalar>
</property>
<property dictRef="me:vibFreqs">
<array units="cm-1"></array>
</property>
<property dictRef="me:spinMultiplicity">
<scalar units="cm-1">2</scalar>
</property>
<property dictRef="me:hessian">
<matrix matrixType="squareSymmetricLT" rows="21" units="Hartree/Bohr2">
0.0258854

0.0275039 0.0244377

0.0033857 -0.0103371 0.0622304

-0.0096416 -0.0061561 0.0043075 0.0084046

-0.0139154 -0.0154045 0.0125931 0.0085224
0.0223638

0.0069051 0.0076918 -0.0151315 -0.0044104 -
0.0111251 0.0105804

-0.012317 -0.0079622 -0.0040578 -0.0013643 -
0.0020139 0.0000181 0.0172648

-0.0064873 -0.0076678 -0.0016697 -0.003922 -
0.0030858 0.0003127 0.0098609 0.0127978

-0.0055188 -0.003121 -0.0114483 0.0007629
0.0006394 0.0019291 0.0065745 -0.004205 0.0369462

-0.0121259 -0.00285 -0.005724 0.0026596 -
0.0004357 0.0020153 -0.0018039 -0.0008533 -0.0081419 0.0550853

-0.0023895 -0.0103498 0.0073457 -0.0018043 -
0.0037723 0.0096713 -0.0016403 0.0008707 -0.0047919 0.0107685
0.0529692
-0.0096041 0.0066385 -0.0914178 0.0006849
0.0025084 -0.0022569 -0.0008515 0.0002591 -0.0031912
0.0315613 -0.0315181 0.338157

-0.0018628 0.0021129 -0.0137615 0.0000882
0.0005457 0.0014363 -0.008822 -0.0041529 -0.0017462 -

0.0016521 -0.0045039 -0.00172 0.0416993

-0.0005276 0.000763 -0.0089559 0.0011033
0.0005193 0.0002927 -0.001569 -0.0123679 0.0294336 -
0.0052918 0.0003688 -0.0007966 0.0123932 0.0430987

-0.0025972 0.0002549 -0.0075225 0.0015092
0.0008575 0.0004211 -0.0116387 0.0212126 -0.1139264 -0.002933
-0.0012461 0.001222 0.0502132 -0.0881839 0.4769838

0.003493 -0.0082301 0.001946 0.0004611
0.0053107 -0.0022805 -0.0022843 0.0007593 -0.0001382 -0.003699
0.0036997 0.0009461 0.0003433 -0.0007541 0.0003835
0.0177565
-0.0029146 0.0040309 -0.001592 0.0006913 -
0.0049773 0.0015798 -0.0004635 -0.0002413 0.000011 0.002312 -
0.0014533 -0.000406 -0.0002966 0.0002563 0.0001947 0.0141406
0.0136809
-0.0011599 0.0021824 -0.0026586 -0.0005138 -
0.0012695 0.001077 -0.0001896 -0.0000718 0.0003817 0.003488 -
0.0028806 -0.0003004 -0.0000357 0.0004177 0.0000452 0.0063632
0.0052645 0.0034847
-0.0017985 -0.0051677 -0.0009503 0.0006487
0.0008365 -0.0007027 -0.0011039 0.0007138 0.0002563 -
0.0002464 0.0009678 0.0002065 -0.0001879 -0.0004208 0.0005147 -
0.0721827 -0.0492 -0.0219979 0.2960425
0.0008728 0.001091 0.0001163 0.0008689 -
0.0005463 0.0002675 0.0012774 0.0002339 0.0002942 0.0002857 -
0.0002602 -0.0000102 0.0003703 0.0000837 -0.0001561 -0.0550571 -
0.0464325 -0.0207017 0.207135 0.1826483
-0.000388 0.0018846 0.0006102 -0.0004847 -
0.0000031 -0.0000608 0.0009872 0.0000852 0.0002318 0.0016437 -
0.0005571 -0.0009011 0.0003885 -0.0002017 -0.0001969 -0.023763 -
0.0216056 -0.0102622 0.0919805 0.0800055 0.0391964
</matrix>
</property>
<property dictRef="me:sigma" default="true">
<scalar>5.0</scalar>
</property>
<property dictRef="me:epsilon" default="true">
<scalar>50.0</scalar>
</property>
<property dictRef="me:frequenciesScaleFactor" default="true">
<scalar>1</scalar>
</property>

<property dictRef="me:symmetryNumber" default="true">
<scalar>0.5</scalar>
</property>
</propertyList>

<me:DOSCMethod xsi:type="me:ClassicalCoupledRotors">
<me:MCPoints>500000</me:MCPoints>
<me:RotorArray>
<me:Rotor bondRef="b1" periodicity="1">
</me:Rotor>
<me:Rotor bondRef="b2" periodicity="1">
</me:Rotor>
</me:RotorArray>
<me:ExpansionSize>
<property dictRef="size">
<array >5 6</array>
</property>
</me:ExpansionSize>
<me:FourierSeries>
<property dictRef="coeficients">
<array units="kJ/mol">2833.2439 836.8656 -76.4979 199.6138
27.9165 -35.9128 -640.7847 -73.2517 -72.7849 -8.1487
33.3622 60.2951 -1054.8877 -37.8081 -130.1968 -35.3735
-50.9609 -73.7616 42.9075 43.8356 40.5185 6.288 -
1.5579 5.2 4.4682 13.4492 6.9793 4.89 -4.0161 -
26.3849</array>
</property>
</me:FourierSeries>
<me:FourierSeries>
<property dictRef="coeficients">
<array units="kJ/mol">0 -1267.6895 -408.3293 31.282 -52.3691
-8.2197 0 294.769 150.3585 96.6977 58.3441
13.7986 0 -246.1411 -154.0667 -58.2076 -50.8218 -
16.8861 0 -4.2228 -4.6144 -8.5627 2.8722 1.1922
0 4.7111 9.0335 -10.8638 -19.9555 -6.0344</array>
</property>
</me:FourierSeries>
<me:FourierSeries>
<property dictRef="coeficients">
<array units="kJ/mol">0 0 0 0 0 0 -391.7702
569.6217 250.6129 52.1593 33.17 31.6986 -457.1275
120.1039 -95.7667 -115.0256 -60.7627 -32.9034 -46.0601
41.2527 -0.4482 -26.5983 -43.4924 -47.5912 3.5934
12.838 24.1938 0.1416 -0.3844 8.8673</array>
</property>
</me:FourierSeries>
<me:FourierSeries>
<property dictRef="coeficients">
<array units="kJ/mol">0 0 0 0 0 0 0 161.9015
133.2907 7.3573 22.75 7.2674 0 -59.6722 -72.3884
-34.2263 22.2748 -7.5311 0 -95.8017 -113.2678 -
66.484 -34.1037 -10.8925 0 7.0851 -13.2559 -7.2041
0.8388 2.024</array>
</property>
</me:FourierSeries>

```
</me:DOSCMethod>
</molecule>
<molecule spinMultiplicity="2" id="Post3">
<atomArray>
<atom id="a1" elementType="C" x3="1.092810" y3="0.175496"
z3="0.020738"/>
<atom id="a2" elementType="O" x3="0.355551" y3="1.136637"
z3="0.001187"/>
<atom id="a3" elementType="O" x3="0.706523" y3="-1.096977" z3="-
0.028274"/>
<atom id="a4" elementType="H" x3="2.183878" y3="0.246463"
z3="0.080632"/>
<atom id="a5" elementType="H" x3="-0.272242" y3="-1.101711" z3="-
0.079840"/>
<atom id="a6" elementType="O" x3="-1.886176" y3="-0.147812"
z3="0.068940"/>
<atom id="a7" elementType="H" x3="-1.875688" y3="0.667496" z3="-
0.460040"/>
</atomArray>
<bondArray>
<bond atomRefs2="a7 a6" order="1"/>
<bond atomRefs2="a5 a3" order="1"/>
<bond atomRefs2="a3 a1" order="1"/>
<bond atomRefs2="a2 a1" order="2"/>
<bond atomRefs2="a1 a4" order="1"/>
<bond atomRefs2="a5 a6" order="1"/>
</bondArray>
<propertyList>
<property dictRef="me:ZPE">
<scalar units="kJ/mol">-110</scalar>
</property>
<property dictRef="me:vibFreqs">
<array units="cm-1"></array>
</property>
<property dictRef="me:spinMultiplicity">
<scalar units="cm-1">2</scalar>
</property>
<property dictRef="me:hessian">
<matrix matrixType="squareSymmetricLT" rows="21" units="Hartree/Bohr2">
0.63514756 0.10206277 0.23447383 0.04510834 -0.10133634 0.97740156 -
0.16081559 0.00148969 -0.16051799 0.17495275 -0.00041655 -0.07913543
0.04508925 0.00389904 0.03766745 -0.14145932 0.04957171 -0.65896285
0.22525464 -0.04540028 0.73063533 -0.29714226 -0.05208773 -0.04218471 -
0.03191326 -0.00517189 -0.07241505 0.51577471 -0.05413598 -0.07592192
0.00047244 -0.00430353 0.02892632 -0.01049748 0.12545890 0.08720530 -
0.02489535 0.00448542 -0.13771969 -0.07432692 -0.01008382 -0.02443379 -
0.06904104 -0.07846524 0.48154481 -0.14226409 -0.03535148 0.11393944
0.01602912 0.00065607 -0.01392798 -0.03303739 -0.01434041 0.02461310
0.16409166 -0.03690310 -0.08036158 0.04663281 -0.00286312 0.01927359
0.00523490 -0.00933155 0.00756675 0.00636115 0.05135378 0.04735158
```

0.12307486 0.04891254 -0.19474548 0.01058227 0.01131854 -0.04004271 -
0.00855807 -0.00178971 0.00864870 -0.12168543 -0.05836134 0.22477289 -
0.04243875 -0.01693190 0.03807336 0.00727822 0.00233318 0.00338614 -
0.14967110 -0.05097564 0.14226151 -0.00442861 -0.00234169 -0.00265199
0.19137752 -0.01220287 0.00065041 0.00644694 0.00373617 -0.00563628
0.00149519 -0.05651514 -0.04918124 0.07617208 -0.00219729 0.00608387
0.00025169 0.06647960 0.05102427 0.01014964 -0.00048985 0.02711509 -
0.00422366 -0.00111961 -0.01391783 0.17700230 0.08424789 -0.31414507 -
0.00418019 0.00015065 0.00031988 -0.17656786 -0.08129444 0.29501081
0.00684603 0.00055575 0.00404039 0.00008550 0.00012236 -0.00208535 -
0.00240098 -0.00059254 0.00080241 -0.00049930 0.00005751 -0.00068052 -
0.00332531 -0.00093895 -0.00022551 0.31584635 0.00208526 -0.00017155
0.00210907 -0.00236907 0.00105049 -0.00041386 -0.00290692 0.00213780
0.00370128 -0.00022328 -0.00041683 -0.00021030 0.00154634 -0.00471021 -
0.00427695 -0.17880551 0.10719325 -0.01217041 -0.00196317 -0.00925235
0.00230440 0.00068946 0.00588572 0.01356969 0.00608598 -0.01277459
0.00124222 -0.00012562 0.00065223 -0.00181222 -0.00443964 0.00462458
0.16542732 -0.09307392 0.09992261 0.00066709 0.00026291 0.00154117 -
0.00561674 -0.00142221 0.00124692 -0.00160972 -0.00111080 0.00058630
0.00010861 0.00002816 -0.00008111 0.00120802 0.00163849 -0.00195471 -
0.31655228 0.18067319 -0.16856100 0.32179502 -0.00048953 0.00046624
0.00058583 0.00041082 -0.00214614 0.00000982 0.00055434 -0.00073302 -
0.00217088 0.00010261 0.00050261 -0.00012142 -0.00010990 0.00176918
0.00278231 0.17960139 -0.10508296 0.09282691 -0.18006973 0.10522409
0.00019225 0.00081970 -0.00383629 0.00092726 -0.00049355 0.00083611
0.00162688 -0.00005388 -0.00112037 -0.00000115 0.00010744 0.00039449 -
0.00268894 0.00136818 0.00099254 -0.16727873 0.09216468 -0.08905820
0.16722243 -0.09391257 0.09179172
</matrix>
</property>
<property dictRef="me:sigma" default="true">
<scalar>5.0</scalar>
</property>
<property dictRef="me:epsilon" default="true">
<scalar>50.0</scalar>
</property>
<property dictRef="me:frequenciesScaleFactor" default="true">
<scalar>1</scalar>
</property>

<property dictRef="me:symmetryNumber" default="true">
<scalar>1</scalar>
</property>
</propertyList>
<me:DOSCMethod default="true" name="QMRotors" />
</molecule>
</moleculeList>
<reactionList>
<reaction active="true" id="R1">
<reactant>

<molecule role="modelled" ref="FormicAcid"/>
</reactant>
<reactant>
<molecule role="excessReactant" ref="OH"/>
</reactant>
<product>
<molecule role="modelled" ref="Pre1"/>
</product>
<me:MCRCMethod xsi:type="me:MesmerILT">
<me:preExponential units="cm3molecule-1s-1">3.e-10</me:preExponential>
<me:activationEnergy units="cm-1">0.0</me:activationEnergy>
<me:nInfinity>0</me:nInfinity>
</me:MCRCMethod>
<me:excessReactantConc>1E15</me:excessReactantConc>
</reaction>
<reaction active="true" id="R12">
<reactant>
<molecule role="modelled" ref="FormicAcid"/>
</reactant>
<reactant>
<molecule role="excessReactant" ref="OH"/>
</reactant>
<product>
<molecule role="modelled" ref="Pre2"/>
</product>
<me:MCRCMethod xsi:type="me:MesmerILT">
<me:preExponential units="cm3molecule-1s-1">3.e-10</me:preExponential>
<me:activationEnergy units="cm-1">0.0</me:activationEnergy>
<me:nInfinity>0</me:nInfinity>
</me:MCRCMethod>
<me:excessReactantConc>1E15</me:excessReactantConc>
</reaction>
<reaction active="true" id="R2">
<reactant>
<molecule role="modelled" ref="Pre1"/>
</reactant>
<product>
<molecule role="sink" ref="Post1"/>
</product>
<me:MCRCMethod xsi:type="me:RRKM"/>
<me:tunneling xsi:type="me:WKB">
<me:IRCPotential units="kJ/mol" ReducedMass="1">
<me:PotentialPoint ReacCoord="-1.68E-10 " potential="0"/>
<me:PotentialPoint ReacCoord="-1.63E-10 " potential="0.255830828"/>
<me:PotentialPoint ReacCoord="-1.57E-10 " potential="0.503260816"/>
<me:PotentialPoint ReacCoord="-1.51E-10 " potential="0.707186457"/>
<me:PotentialPoint ReacCoord="-1.46E-10 " potential="0.928134852"/>
<me:PotentialPoint ReacCoord="-1.40E-10 " potential="1.183239291"/>
<me:PotentialPoint ReacCoord="-1.35E-10 " potential="1.469973207"/>
<me:PotentialPoint ReacCoord="-1.29E-10 " potential="1.761097035"/>

<me:PotentialPoint ReacCoord="-1.23E-10 " potential="2.073980933"/>
<me:PotentialPoint ReacCoord="-1.18E-10 " potential="2.473020808"/>
<me:PotentialPoint ReacCoord="-1.12E-10 " potential="2.976897475"/>
<me:PotentialPoint ReacCoord="-1.06E-10 " potential="3.524499564"/>
<me:PotentialPoint ReacCoord="-1.01E-10 " potential="4.083376464"/>
<me:PotentialPoint ReacCoord="-9.53E-11 " potential="4.662134298"/>
<me:PotentialPoint ReacCoord="-8.97E-11 " potential="5.242897596"/>
<me:PotentialPoint ReacCoord="-8.40E-11 " potential="5.827924477"/>
<me:PotentialPoint ReacCoord="-7.84E-11 " potential="6.490485422"/>
<me:PotentialPoint ReacCoord="-7.28E-11 " potential="7.304308851"/>
<me:PotentialPoint ReacCoord="-6.72E-11 " potential="8.189776281"/>
<me:PotentialPoint ReacCoord="-6.16E-11 " potential="9.076017473"/>
<me:PotentialPoint ReacCoord="-5.60E-11 " potential="9.991756349"/>
<me:PotentialPoint ReacCoord="-5.04E-11 " potential="10.98690842"/>
<me:PotentialPoint ReacCoord="-4.48E-11 " potential="12.06352653"/>
<me:PotentialPoint ReacCoord="-3.92E-11 " potential="13.22524262"/>
<me:PotentialPoint ReacCoord="-3.36E-11 " potential="14.44476975"/>
<me:PotentialPoint ReacCoord="-2.79E-11 " potential="15.646074"/>
<me:PotentialPoint ReacCoord="-2.24E-11 " potential="16.85480005"/>
<me:PotentialPoint ReacCoord="-1.68E-11 " potential="18.04932995"/>
<me:PotentialPoint ReacCoord="-1.12E-11 " potential="19.50382792"/>
<me:PotentialPoint ReacCoord="-5.61E-12 " potential="21.66257522"/>
<me:PotentialPoint ReacCoord="0.00E+00 " potential="24.07"/>
<me:PotentialPoint ReacCoord="1.12E-11 " potential="22.20238604"/>
<me:PotentialPoint ReacCoord="1.68E-11 " potential="20.7174764"/>
<me:PotentialPoint ReacCoord="2.24E-11 " potential="17.16718029"/>
<me:PotentialPoint ReacCoord="2.80E-11 " potential="14.50372614"/>
<me:PotentialPoint ReacCoord="3.36E-11 " potential="12.12509946"/>
<me:PotentialPoint ReacCoord="3.92E-11 " potential="9.85870199"/>
<me:PotentialPoint ReacCoord="4.48E-11 " potential="7.7029847"/>
<me:PotentialPoint ReacCoord="5.04E-11 " potential="5.7553869"/>
<me:PotentialPoint ReacCoord="5.60E-11 " potential="4.047249585"/>
<me:PotentialPoint ReacCoord="6.16E-11 " potential="2.513217124"/>
<me:PotentialPoint ReacCoord="6.72E-11 " potential="1.096294315"/>
<me:PotentialPoint ReacCoord="7.28E-11 " potential="-0.209820993"/>
<me:PotentialPoint ReacCoord="7.84E-11 " potential="-1.409287794"/>
<me:PotentialPoint ReacCoord="8.40E-11 " potential="-2.507453367"/>
<me:PotentialPoint ReacCoord="8.97E-11 " potential="-3.530353867"/>
<me:PotentialPoint ReacCoord="9.53E-11 " potential="-4.44961901"/>
<me:PotentialPoint ReacCoord="1.01E-10 " potential="-5.246660636"/>
<me:PotentialPoint ReacCoord="1.06E-10 " potential="-5.944756383"/>
<me:PotentialPoint ReacCoord="1.12E-10 " potential="-6.592243955"/>
<me:PotentialPoint ReacCoord="1.18E-10 " potential="-7.185961666"/>
<me:PotentialPoint ReacCoord="1.23E-10 " potential="-7.686717359"/>
<me:PotentialPoint ReacCoord="1.29E-10 " potential="-8.097991008"/>
<me:PotentialPoint ReacCoord="1.35E-10 " potential="-8.452099699"/>
<me:PotentialPoint ReacCoord="1.40E-10 " potential="-8.791609469"/>
<me:PotentialPoint ReacCoord="1.46E-10 " potential="-9.173303329"/>
</me:IRCPotential>
</me:tunneling>

<me:transitionState>
<molecule role="transitionState" ref="TS1"/>
</me:transitionState>
</reaction>
<reaction active="true" id="R3">
<reactant>
<molecule role="modelled" ref="Pre2"/>
</reactant>
<product>
<molecule role="sink" ref="Post2"/>
</product>
<me:MCRCMethod xsi:type="me:RRKM"/>
<me:tunneling xsi:type="me:WKB">
<me:IRCPotential units="kJ/mol" ReducedMass="1">
<me:PotentialPoint ReacCoord="-2.40897E-10 " potential="0"/>
<me:PotentialPoint ReacCoord="-2.3336E-10 " potential="0.19857457"/>
<me:PotentialPoint ReacCoord="-2.25824E-10 " potential="0.494512622"/>
<me:PotentialPoint ReacCoord="-2.18287E-10 " potential="0.774007753"/>
<me:PotentialPoint ReacCoord="-2.1075E-10 " potential="1.027604854"/>
<me:PotentialPoint ReacCoord="-2.03213E-10 " potential="1.267571413"/>
<me:PotentialPoint ReacCoord="-1.95675E-10 " potential="1.491923236"/>
<me:PotentialPoint ReacCoord="-1.88137E-10 " potential="1.705232595"/>
<me:PotentialPoint ReacCoord="-1.806E-10 " potential="1.926323441"/>
<me:PotentialPoint ReacCoord="-1.73061E-10 " potential="2.18685659"/>
<me:PotentialPoint ReacCoord="-1.65523E-10 " potential="2.585213501"/>
<me:PotentialPoint ReacCoord="-1.57985E-10 " potential="2.996562561"/>
<me:PotentialPoint ReacCoord="-1.50447E-10 " potential="3.429064842"/>
<me:PotentialPoint ReacCoord="-1.42908E-10 " potential="3.882064698"/>
<me:PotentialPoint ReacCoord="-1.3537E-10 " potential="4.337324809"/>
<me:PotentialPoint ReacCoord="-1.27831E-10 " potential="4.806612301"/>
<me:PotentialPoint ReacCoord="-1.20292E-10 " potential="5.283715787"/>
<me:PotentialPoint ReacCoord="-1.12754E-10 " potential="5.750018362"/>
<me:PotentialPoint ReacCoord="-1.05215E-10 " potential="6.203363295"/>
<me:PotentialPoint ReacCoord="-9.76761E-11 " potential="6.661798114"/>
<me:PotentialPoint ReacCoord="-9.01374E-11 " potential="7.159450937"/>
<me:PotentialPoint ReacCoord="-8.25986E-11 " potential="7.635156861"/>
<me:PotentialPoint ReacCoord="-7.50603E-11 " potential="8.081997092"/>
<me:PotentialPoint ReacCoord="-6.75216E-11 " potential="8.500523754"/>
<me:PotentialPoint ReacCoord="-5.99828E-11 " potential="8.994277206"/>
<me:PotentialPoint ReacCoord="-5.2444E-11 " potential="9.524367269"/>
<me:PotentialPoint ReacCoord="-4.49052E-11 " potential="10.05689012"/>
<me:PotentialPoint ReacCoord="-3.7367E-11 " potential="10.57310809"/>
<me:PotentialPoint ReacCoord="-2.98282E-11 " potential="11.07257257"/>
<me:PotentialPoint ReacCoord="-2.22905E-11 " potential="11.61541322"/>
<me:PotentialPoint ReacCoord="-1.47565E-11 " potential="12.5514864"/>
<me:PotentialPoint ReacCoord="-7.22297E-12 " potential="14.01330167"/>
<me:PotentialPoint ReacCoord="0 " potential="15.18345784"/>
<me:PotentialPoint ReacCoord="1.4876E-11 " potential="8.208378558"/>
<me:PotentialPoint ReacCoord="2.24121E-11 " potential="-3.02982026"/>
<me:PotentialPoint ReacCoord="2.99488E-11 " potential="-21.07955626"/>

<me:PotentialPoint ReacCoord="3.74759E-11 " potential="-38.25932693"/>
<me:PotentialPoint ReacCoord="4.43228E-11 " potential="-41.54502534"/>
<me:PotentialPoint ReacCoord="5.18129E-11 " potential="-44.63688204"/>
<me:PotentialPoint ReacCoord="5.93385E-11 " potential="-48.03133537"/>
<me:PotentialPoint ReacCoord="6.68688E-11 " potential="-51.39107705"/>
<me:PotentialPoint ReacCoord="7.44001E-11 " potential="-54.25116865"/>
<me:PotentialPoint ReacCoord="8.19336E-11 " potential="-56.51696223"/>
<me:PotentialPoint ReacCoord="8.94692E-11 " potential="-58.60395995"/>
<me:PotentialPoint ReacCoord="9.70059E-11 " potential="-60.47759838"/>
<me:PotentialPoint ReacCoord="1.04544E-10 " potential="-62.118607"/>
<me:PotentialPoint ReacCoord="1.12082E-10 " potential="-63.49313886"/>
<me:PotentialPoint ReacCoord="1.19621E-10 " potential="-64.44851213"/>
<me:PotentialPoint ReacCoord="1.2716E-10 " potential="-65.53912495"/>
<me:PotentialPoint ReacCoord="1.34699E-10 " potential="-66.62934247"/>
<me:PotentialPoint ReacCoord="1.42238E-10 " potential="-67.67575661"/>
<me:PotentialPoint ReacCoord="1.49777E-10 " potential="-68.64679336"/>
<me:PotentialPoint ReacCoord="1.57316E-10 " potential="-69.5283457"/>
<me:PotentialPoint ReacCoord="1.64854E-10 " potential="-70.32634303"/>
<me:PotentialPoint ReacCoord="1.72393E-10 " potential="-70.84746246"/>
<me:PotentialPoint ReacCoord="1.79932E-10 " potential="-71.36541954"/>
<me:PotentialPoint ReacCoord="1.87471E-10 " potential="-71.90931772"/>
<me:PotentialPoint ReacCoord="2.02549E-10 " potential="-72.94308249"/>
<me:PotentialPoint ReacCoord="2.32705E-10 " potential="-74.80837036"/>
<me:PotentialPoint ReacCoord="2.40244E-10 " potential="-75.20689962"/>
<me:PotentialPoint ReacCoord="2.47783E-10 " potential="-75.56538077"/>
<me:PotentialPoint ReacCoord="2.55321E-10 " potential="-75.8888291"/>
<me:PotentialPoint ReacCoord="2.70399E-10 " potential="-76.44226371"/>
<me:PotentialPoint ReacCoord="2.77937E-10 " potential="-76.67874763"/>
<me:PotentialPoint ReacCoord="2.85475E-10 " potential="-76.89005633"/>
<me:PotentialPoint ReacCoord="2.93012E-10 " potential="-77.0777957"/>
<me:PotentialPoint ReacCoord="3.00549E-10 " potential="-77.22879755"/>
</me:IRCPotential>

</me:tunneling>
<me:transitionState>
<molecule role="transitionState" ref="TS2"/>
</me:transitionState>
</reaction>
<reaction active="true" id="R32">
<reactant>
<molecule role="modelled" ref="Pre1"/>
</reactant>
<product>
<molecule role="sink" ref="Post3"/>
</product>
<me:MCRCMethod name="RRKM"/>
<me:transitionState>
<molecule role="transitionState" ref="TSadd"/>
</me:transitionState>
<me:tunneling me:useZPE = "True" xsi:type="Eckart"/>

</reaction>

</reactionList>
<me:conditions>
<me:bathGas>He</me:bathGas>
<me:PTs>

<me:PTpair units="Torr" P=" 15 " T=" 298 "
precision="d"><me:experimentalEigenvalue EigenvalueID="1"
error="35">350</me:experimentalEigenvalue></me:PTpair>
<me:PTpair units="Torr" P=" 15 " T=" 423 "
precision="d"><me:experimentalEigenvalue EigenvalueID="1"
error="32">322</me:experimentalEigenvalue></me:PTpair>
<me:PTpair units="Torr" P=" 15 " T=" 521 "
precision="d"><me:experimentalEigenvalue EigenvalueID="1"
error="41">418</me:experimentalEigenvalue></me:PTpair>
<me:PTpair units="Torr" P=" 15 " T=" 621 "
precision="d"><me:experimentalEigenvalue EigenvalueID="1"
error="58">585</me:experimentalEigenvalue></me:PTpair>
<me:PTpair units="Torr" P=" 15 " T=" 694 "
precision="d"><me:experimentalEigenvalue EigenvalueID="1"
error="71">714</me:experimentalEigenvalue></me:PTpair>
<me:PTpair units="Torr" P=" 15 " T=" 773 "
precision="d"><me:experimentalEigenvalue EigenvalueID="1"
error="85">852</me:experimentalEigenvalue></me:PTpair>
<me:PTpair units="Torr" P=" 15 " T=" 860 "
precision="d"><me:experimentalEigenvalue EigenvalueID="1"
error="129">1290</me:experimentalEigenvalue></me:PTpair>

</me:PTs>
<me:InitialPopulation>
<me:molecule grain="1.0" population="1.0" ref="FormicAcid"/>
</me:InitialPopulation>
</me:conditions>
<me:modelParameters>
<me:grainSize units="cm-1">25</me:grainSize>
<me:energyAboveTheTopHill>30.0</me:energyAboveTheTopHill>
</me:modelParameters>
<me:control>
<!--><me:calcMethod xsi:type="me:marquardt">
<me:MarquardtIterations>14</me:MarquardtIterations>
<me:MarquardtTolerance>0.0001</me:MarquardtTolerance>
<me:MarquardtDerivDelta>5.e-02</me:MarquardtDerivDelta>
</me:calcMethod>-->
<me:printTunnelingCoefficients/>
<me:eigenvalues>6</me:eigenvalues>
</me:control>
</me:mesmer>
